# A Citizen Science Facemask Experiment and Educational Modules to Improve Coronavirus Safety in Communities and Schools

**DOI:** 10.3389/fmed.2020.00486

**Published:** 2020-09-03

**Authors:** Sarah E. Eichler, Austin P. Hopperton, Juan José Alava, Antonio Pereira, Rukhsana Ahmed, Zisis Kozlakidis, Sanja Ilic, Alexander Rodriguez-Palacios

**Affiliations:** ^1^Department of Biological Sciences, Kent State University at Salem, Salem, OH, United States; ^2^Division of Gastroenterology and Liver Disease, School of Medicine, Case Western Reserve University, Cleveland, OH, United States; ^3^Institute for Oceans and Fisheries, University of British Columbia, Vancouver, BC, Canada; ^4^Institute of Technology, Federal University of Pará, Belém, Brazil; ^5^Department of Communication, University at Albany, SUNY, Albany, NY, United States; ^6^International Agency for Research on Cancer, World Health Organization, Lyon, France; ^7^Department of Human Sciences, The Ohio State University, Columbus, OH, United States; ^8^University Hospitals Research and Education Institute, University Hospitals Cleveland Medical Center, Cleveland, OH, United States

**Keywords:** COVID-19, mass media, health communication, prevention, intervention, social behavioral changes, facemask, school education

## Introduction

According to the UNICEF, children between 0–14 years represent ~26% of the total global population (~45% in Africa; 22% in USA, of which 90% attend school; ranging from 85–100% across countries) (1). With high case-fatality ratios between 4.5–7.5% (Germany/Iran/USA/Brazil/Canada) and 11.9–16.4% (Spain/Italy/UK/France/Belgium) (2), there is a critical need to empower citizens, especially children (often asymptomatic carriers), with education strategies to control COVID-19. Especially, there is need to support facemask citizen science and experiential education among children and families as the globe exits from the current lockdown, and teachers and students desire and seek for safe strategies to return to densely-attended schools. COVID-19 is a pandemic respiratory disease that disseminates as infectious respiratory or saliva droplets are released into the environment as people talk, sneeze, and cough (3–5). Currently the most publicized methods to prevent local transmission of COVID-19 and promote “droplet safety” in hospitals and communities include hand washing, social distancing, and stay-at-home strategies. In contrast to established benefits for medical masks in hospitals, the benefits of wearing masks or face covers/coverings (hereafter, “facemask”) in the community have been inconsistently debated by the media, creating confusion, and misinformation (6). Furthermore, high-profile political leaders in countries heavily affected by the pandemic have given misleading signs regarding containment measures associated with COVID-19 (7–11) increasingly polarizing local communities around arguments on the value of facemasks in promoting public health, which is critically important to incentivize during the emergence of citizens from their lockdowns and during the phase of reopening local economies.

## Droplet and Facemask Science to Empower Community Action

Two of the most important measures recommended for public areas are social distancing and wearing facemasks to prevent COVID-19. Of these, facemasks predetermines the minimum person-person distance required in social distancing. That is, facemask utilization allows for safer interactions at closer distances because droplets are contained within the mask. However, such concept is not well publicized and facemasks value have received contradictory attention. Inconsistent compliance with droplet control strategies now threatens COVID-19 containment. Governments that led initiatives against COVID-19 have reversed orders on facemasks as “mandatory” due to protests, highlighting that cultural acceptability is necessary for the success of any measure intended to control COVID-19. The science of facemasks is straightforward (12), facemasks stop (97.2–99.7%) droplets dispersion (12–15). Thus, the reversal of mask-wearing mandates leaves education and self-awareness as our ultimate strategies to ensure equitable and sustained public droplet safety.

Adapting a recent spray-bottle “sneeze-simulation” technique (14), we propose an educational module to promote citizen science for greater understanding of facemask effectiveness to protect the environment and the general public. Completion of simple home or school-based activities followed by online, volunteered data submission, with graphical feedback summaries encourages understanding, and quantitative learning about facemasks. Our module will help improve the knowledge about germ dissemination by droplets, which is less confusing than teaching the abstract concept of prevention of disease for the wearer. This citizen science educational campaign could inspire the sustained routine wearing of facemasks and solidarity in the community, leading to COVID-19 reduction. Ubiquitous use of facemasks combined with international and multi-disciplinary cooperation at a global scale are necessary to overcome the pandemic (16) and also to prevent further infections and overburdened healthcare systems as recently forecasted (17).

## Covid-19 Communication and Future Education

Promoting established and new approaches to protect human health, effective public messaging, and health education programs are paramount during infectious disease outbreaks (18). This is especially true in the era of COVID-19 so that a second global wave of coronavirus infections does not surpass the first wave. COVID-19 has created many challenges and fears for society because of its high infection rate, rapid community transmission, and high mortality rate, in particular for the elderly and those with underlying health conditions. There are various health challenges across urban/rural gradients that may impact the future local rates of associated mortality. These include sanitation infrastructure, hygiene and health education, implementation of prevention measures, and variable access to health care relevant to COVID-19 diagnosis, monitoring, and treatment.

Moving forward, public health approaches need to remain nimble to allow improvement in interventions and research tools quickly enough to stay ahead of the pandemic trajectory. New collaborative efforts are vital to ensure that the health needs in various communities can be met (19, 20). One such approach is appropriate access to health education programs (21), and evidence-based solutions such as home-made, double-layered facemasks (22). Thus, communities are empowered equitably and sustainably to improve health outcomes through proactive citizen science and education (23). Increasing community understanding of personal and community prevention measures and widespread signage promoting droplet education and facemask use (24) could alleviate personal conflicts surrounding citizen-citizen requests to wear facemasks in public. Well-communicated and unified educational campaigns are important in primary/high-school and higher education institutions, where the majority of individuals are eager to return (to often highly-populated classrooms), and because facemask compliance and social distancing within most young communities are suboptimal for various reasons. Returning to schools without clarifying with students the science of droplets and facemasks will facilitate the spread or misinformation and conceptual polarization. With ~26% of the population attending schools, education leaders have the great opportunity and potential to authoritatively help improve our collective knowledge and understanding of how and why it is important to increase the coronavirus safety in their local communities.

## Contradicting Guidelines on Facemasks

Ongoing debates about the usefulness of facemasks for the public developed in response to contradicting global health directives widely-publicized early in the COVID-19 emergency (6, 25–30). International health and political leaders insisted that mask use by non-healthcare workers would not protect the public, but rather would exacerbate supply shortages needed for hospitals (31, 32). This logic was reiterated in numerous regions, including Australia, where similar “anti-masking” arguments featured prominently (33).

Reportedly, healthcare workers experienced high rates of infections and mortality, partly because insufficient access to personal protection equipment (PPE) increased droplet exposure risk (34, 35). As PPE shortages worsened, governments, and institutions resorted to public requests for PPE (e.g., using social media #GetMePPE) (36). Latin America has also experienced widespread PPE shortages, e.g., Ecuador was assisted by the Pan-American Health Organization with PPE in April 2020 (37).

Being increasingly aware of foreseen shortages, hospital systems made calls for donations of homemade facemasks for use by caregivers (38–42). As part of pandemic containment strategies, more than 50 countries outside the US have mandatory facemask policies (43). In those countries, there is apparent cultural acceptance and adoption of facemask guidelines; however, while the precautionary principle applies, more research on implementation measures is needed (44). As of May 11, 2020 several states removed their requirements for facemasks in public (45).

Further challenges recently emerged as local violence, conflict, and publicized protests attracted global public attention leading to some high-profile public officials reverting orders or declining to wear facemasks in public (46). Some of the reasons cited were political in origin (e.g., US civil rights movement legacy, and fears of increased racial profiling), and some reflected a lack of information regarding “what a facemask will or will not do” in reducing COVID-19. The lack of clear information, combined with politicization in certain regions, could lead to medical advice being discounted–even though most people consider the use of facemasks protective (47, 48), which is in agreement with data showing reduction of transmission. Clear guidance and the dissemination of factual information in the community is needed as part of citizen science and education campaigns. Contributing to such educational strategies to inform and incentivize the public, our laboratory recently determined that textiles, when used as 2-layer-covers, can reduce the contamination of the environment with bacteria-carrying droplets by 99.7%, which also fully protects germ-free mice when their cages are covered with 2-layer textiles (13, 14, 49). To share this important finding with the public, we created an educational module on the effectiveness of facemasks, described in brief below and freely accessible in English, French, Spanish, and Portuguese at https://sites.google.com/kent.edu/face-mask-challenge/face-mask-challenge-home, which address many regions facing severe rates of COVID19 infection.

## Droplet Science and Door Sign Education

Telling people that they did not need masks early in the pandemic created public distrust, because official messages assumed that the public was unable to process facts, specifically, that masks indeed protect health-workers (6). To regain public confidence, we propose that there is a need to provide detailed information to the public regarding the basics of droplet science. The physics of droplet dynamics in disease dispersion has been studied for decades in agriculture and plant sciences as a means to understand disease control (50, 51). In medicine, there is increasing interest in the dynamics of human droplet production and contamination of the environment (4, 5, 49, 52–56).

Except for health, food, and some industry workers, it is sensible to assume that most people never used facemasks before the pandemic. Thus, droplet education campaigns to prevent respiratory pandemics should acknowledge the novelty of facemask interventions so that knowledge discovery via experimentation translates into greater compliance. Communities need instruction that masks should be worn effectively, and basic droplet science recognized.

Clinical observations have shown that masks do not always prevent disease in people at high risk, especially, because subjects often (~50%) do not use masks properly, or all the time, and because ancillary measures like “handwashing” and “not touching their face” are often lax (57, 58). Prevention failures can be corrected with educational campaigns aimed at a broad range of public but especially school children provided that instructions emphasize practice. And practice can be achieved through hands-on activities.

**Figure 1:**
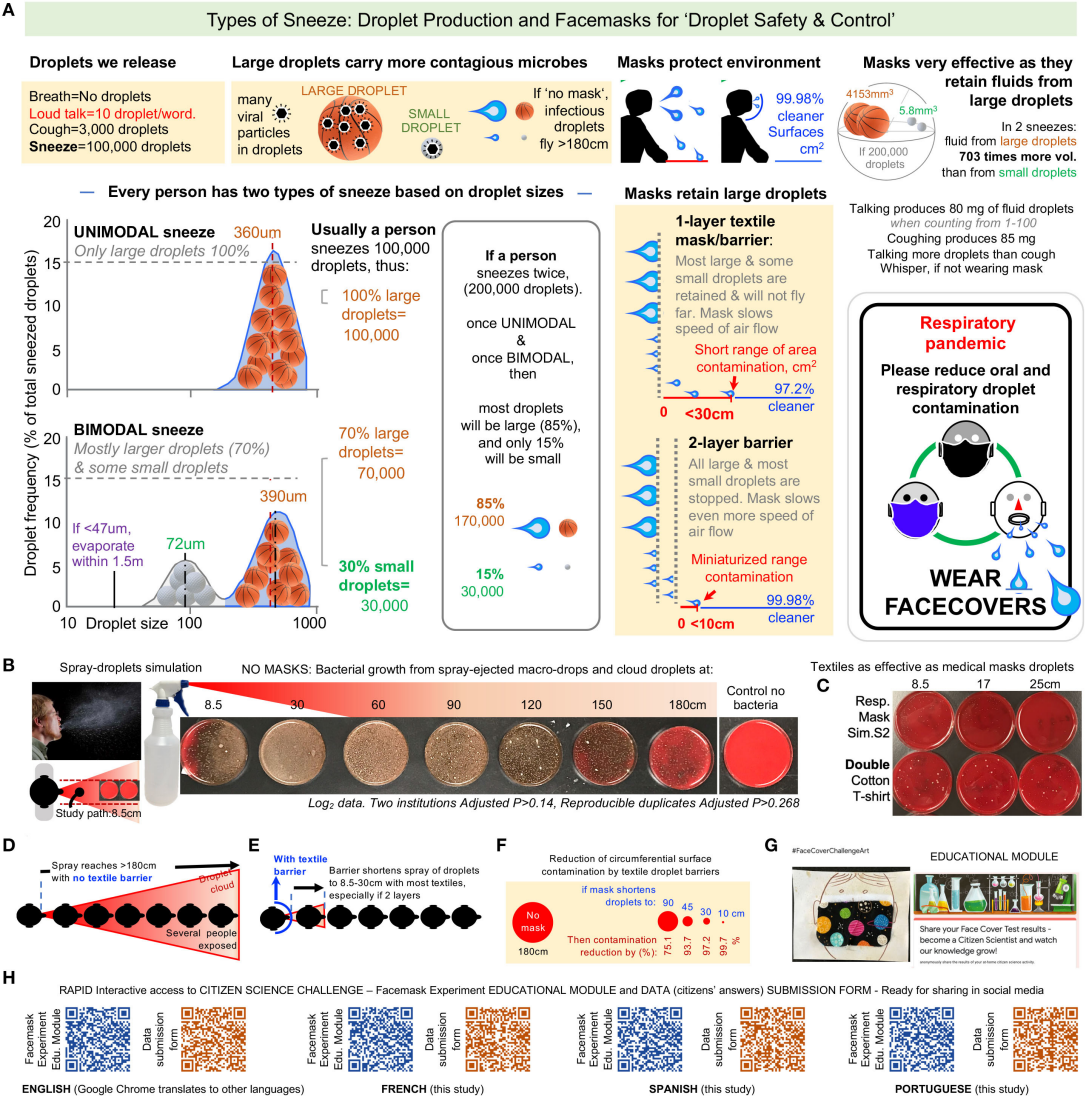
Detailed educational citizen science modules could be based on information on droplet biology and experiments to increase experiential knowledge, facemask compliance, and public droplet safety. **(A)** Human sneezes produce the most droplets that are spread farther than 6 ft (180 cm) in which most are large droplets that contain far more viral particles than small droplets. Sneezes result in large droplets followed by a mix of large and small droplets in sizes proportional to a basketball relative to a golf ball, respectively. Virtually all large droplets and most small droplets are contained by a two-layer textile barrier, which reduces the resulting area of contamination (14, 55, 59, 60). Communicating the importance of face covers via door signs can reduce person-to-person verbal interactions and lessen exposure risk (see https://figshare.com/articles/Door_Signs_to_Promote_Public_Droplet_Safety_Amidst_COVID-19/12202808). **(B)** Infectious droplets can easily travel 180 cm (the recommended social distancing space) without a barrier as evidenced by bacterial colony forming units on agar plates in the line of culture spray, whereas **(C)** a loose double-layered t-shirt barrier reduced transmission of infectious particles nearly as thoroughly as a medical mask (49) **(D–F)**. A face cover results in 99.98% cleaner environmental surface following two sneezes- protecting both the wearer and others who may contact surfaces. **(G)** An example of a citizen science facemask experiment project can be accessed at Supplementary Material and the data report form at https://docs.google.com/forms/d/e/1FAIpQLSd9cV7HQzxr49MsC-icHCzxIOnlhX2z7e7iza3cJ-NGzJaFRw/viewform. **(H)** To rapidly enable the visualization of the module on mobile smart phones, reader should activate their cameras, point the camera to the QR-code, and then wait a couple of seconds for the phone to direct them to the website where the module or the answer submission forms are hosted. Studies supporting the droplet size and types of sneeze concepts in this graphical summary were adapted from (55, 59, 60). **(C–E)**, Unmodified from Rodriguez-Palacios et al. (14) under open CC BY license http://creativecommons.org/licenses/by/4.0/.

Viruses live and replicate in liquid phases inside cells and as such can only be expelled in liquid droplets as mucus/saliva in company with other more abundant microorganisms that also live in the respiratory/oral system, including other viruses, bacteria, and fungi. To correct such source of confusion among citizens, herein we provide a graphical summary of the biology and physics of droplets in humans as they speak, cough, and sneeze (Figure 1A). Also we provide customizable door sign examples to promote public droplet safety amidst COVID-19 to promote widespread awareness while decreasing the need for citizen-citizen verbal interactions, reducing conflict risk [see resource in (24)]. By simulating sneeze droplet dynamics using recently validated rapid spray-bottle methods and inocuous bacterial suspensions (14), we also illustrate that mask wearing reduces the range of droplet dispersion, increasing the margin of droplet safety within person-person interaction distances (Figures 1B–F).

## Citizen Science: Droplet and Facemask Experiments For Communities and Schools

A community-based approach to enhance public health preparedness was fundamental during past emergencies with Hurricane Katrina (2005), and the H5N1 avian influenza (2004) and H1N1 pandemic (2009) (61). Local community preparedness during emergencies is recognized as an important goal but remains challenging to implement and monitor (62–64). During global emergencies, integrated approaches to delivering community health services can significantly improve the emergency management by creating supportive social contexts within which communities can withstand and recover from public health emergencies (65, 66). Appropriate communication of health response actions -or lack thereof- can impact both epidemiologic and economic trends (67). Integration of equity, health literacy (including information, scientific, digital, and numeracy literacies) (68), cultural tailoring (69), and educational efforts must all be considered to provide clear instruction and improved understanding of the benefits of facemask use in public. In our view, a major educational campaign is needed in order to build community cooperation toward wide scale use of face coverings to increase community resilience to both current and future infectious disease outbreaks such as COVID-19.

As a response to this call, an educational exercise and citizen science project is presented here to promote discussion and greater awareness of facemask effectiveness by providing an innovative hands-on activity. Based on a validated spray simulation method (13, 14), community members can observe first-hand how textile barriers stop the dispersal of colored droplets, made with coffee for instance, or stop the dispersal of germs using solutions containing microbes safely present in foods like yogurt or garden soil. The educational module contains four experiments of varying difficulty and is freely available as an archive of latest versions and community contributions (https://github.com/axr503/education), as a website in multiple languages (English, Spanish, French and Portuguese, specifically developed for this project, or amenable for automatic translation via online search engines such as Google Chrome, see also Figure 1G-H for direct smartphone access) at https://sites.google.com/kent.edu/face-mask-challenge/face-mask-challenge-home or as 'ready-to-print' PDF module files for direct public access in four languages (see Supplementary Materials).

The online data submission form where the citizen scientists (adults, supervised children, or teachers) can share the results of their experiments with the global community, and see the aggregate responses globally or for their respective languages, is available at https://bit.ly/facemaskchallengedata.

This project is inspired by recent spray-simulation research that shows the effectiveness of cloth covers in reducing the contamination of the environment by liquid droplets (13, 14). Facemasks drastically cut environmental contamination to <8 cm (>99.7% reduction, if two-layers are used) compared to a radial area of contamination of 2 m if we do not wear droplet barriers (13, 14). Our module also helps demonstrate how many droplets people release into the environment during normal speech.

## Educational Module Contains Four Experiments Available in Multiple Languages

To enable a more uniform implementation of such education activities, the experiments are provided translated in four languages. Additional translations will also be possible. The educational module contains an introduction for teachers or parents and simple instructions for each of four home experiments that adults and supervised children could choose to complete. The activities are suitable for “citizen scientists” with at least a third-grade reading comprehension. In the activity, participants record the distance of droplet travel without any covering and compare dispersal patterns to those of a simulated sneeze that has been covered with a cloth. These citizen scientists can further investigate the spread of germs with and without a face cover by using home-made gelatin microbial growth plates to intercept and visualize germs spread through simulated sneeze or speech. Participants can choose to share their observations through an anonymous online (IRB-approved) form. Volunteered responses from the “#FaceMaskChallenge” will build a citizen science database and display simple graphs that illustrate the reduction in droplet spread.

The citizen science activity module is presented without political motivation or emotional language and adheres to basic scientific methods approach to knowledge discovery. Through the completion of a few hands-on activities, participants will discover details related to the effectiveness of facemasks including affordable and accessible home-made face covers. Questions and talking points in the module will encourage participants to think about community spread germs and their own choices regarding masks. Engagement with the science of public droplet safety may help move beyond the sensationalized discourse currently dominating the media and contribute to pandemic preparedness actions. This citizen science project with experiential educational modules (entitled - Facemask Challenge - A COVID-19 Educational Campaign To Promote Public Droplet Safety) is critically important to promote coronavirus safety. The educational campaign presented here and other experiential learning will support plans that ensure a safe return of students to classrooms, to protect children, prevent the asymptomatic dispersion of the virus, and protect vulnerable teachers, many of whom in the US school system (~30%) are 50 years or older (70).

## Conclusion

Scientific discoveries are often communicated to the public with uncertainty, unintentionally creating confusion among non-scientists. To control COVID-19, social distancing alone, as widely promoted (e.g., in radio stations, without encouraging facemasks), will be impossible to sustain as communities exit lockdowns. In the communication process, it is therefore important to realize that empowering citizens with tools to numerically visualize the benefits of a healthy behavior could facilitate the sustained experiential-based adoption of facemasks. The use of citizen science modules, as herein proposed, could improve cultural acceptability, community resilience and facilitate the development of educational strategies and policies to promote public droplet safety to control COVID-19. A straightforward “Test-your-Facemask” challenge and droplet experiment modules are herein released as a paper-and-online open-access resource for the benefit of the community. These resources can promote coronavirus safety in schools -protecting vulnerable populations, including experienced teachers, and families in local school districts as institutions seek strategies for a safe return to classrooms this year.

## Author Contributions

SE and AR-P developed the opinion concept. SE provided an initial draft and edited all versions. AR-P provided figure and edited all drafts. SE, AH, and SI tested supplemental module procedure. All the authors contributed substantially to discussion, development of text, corrections, and edits to the supplemental educational module and implementation to necessary translations. SE, AH, and AR-P prepared Supplementary Material to which all authors contributed. Individual authors provided translation of the Facemask Challenge module for Spanish (JA), French (ZK), and Portuguese (AP).

## Conflict of Interest

The authors declare that the research was conducted in the absence of any commercial or financial relationships that could be construed as a potential conflict of interest.

## Supplementary Material

Supplementary Material A compiled PDF document of the facemask challenge educational activity modules amenable for schools in English, French, Spanish and Portuguese. The links to the websites can be accessed from **Figures 1G,H** using smartphone cameras and the QR-codes.
