# Supplementary material for: A Citizen Science Facemask Experiment and Educational Modules to Improve Coronavirus Safety in Communities and Schools
Source: Front Med (Lausanne). 2020 Sep 3;7:486. doi: 10.3389/fmed.2020.00486 (PMC7494729; doi:10.3389/fmed.2020.00486)
Supplement: Supplementary Material — A compiled PDF document of the facemask challenge educational activity modules amenable for schools in English, French, Spanish and Portuguese. The links to the websites can be accessed from Figures 1G,H using smartphone cameras and the QR-codes. [file Data_Sheet_1.pdf]

frontiers in Medicine

OPINION
published: xx July 2020
doi: 10.3389/fmed.2020.00486

Check for updates

A Citizen Science Facemask Experiment and Educational Modules to Improve Coronavirus Safety in Communities and Schools

Sarah E. Eichler[1], *Austin P. Hopperton*[2], *Juan José Alava*[3], *Antonio Pereira Jr.*[4], *Rukhsana Ahmed*[5], *Zisis Kozlakidis*[6], *Sanja Ilic*[7] *and Alexander Rodriguez-Palacios*[2*]

[1] Department of Biological Sciences, Kent State University at Salem, Salem, MA, United States, [2] Division of Gastroenterology and Liver Disease, School of Medicine, Case Western Reserve University, Cleveland, OH, United States, [3] Institute for Oceans and Fisheries, University of British Columbia, Vancouver, BC, Canada, [4] Institute of Technology, Federal University of Pará, Belém, Brazil, [5] Department of Communication, University at Albany, SUNY, Albany, NY, United States, [6] International Agency for Research on Cancer, World Health Organization (France), Lyon, France, [7] Department of Human Sciences, The Ohio State University, Columbus, OH, United States

Keywords: COVID-19, mass media, health communication, prevention, intervention, social behavioral changes, facemask, school education

OPEN ACCESS

SUPPLEMENTARY MATERIALS

Education Module #1 ENGLISH
Education Module #2 SPANISH
Education Module #3 FRENCH
Education Module #4 PORTUGUESE

Education Module

ENGLISH

A CITIZEN SCIENCE Facemask Experiment

Educational Campaign and Modules to Promote *Coronavirus Safety* in Communities and Schools

COVID-19 is a viral disease caused by coronavirus that primarily spread through oral and nasal fluids or droplets. COVID-19 most seriously affects the elderly and those already sick, causing shortness of breath, fever, coughing, muscle pains and systemic inflammation that can lead to hospitalization.

COVID spreads from person-to-person via saliva and nasal microdroplets. Because of this, health experts recommend keeping a 6-foot (1.8 m) distance from other people, limiting exposure to groups, washing hands and wearing face covers. Since medical masks are scarce in some places, many people use face coverings such as scarves and handkerchiefs. **The main function of face coverings** or generic masks is not to filter the air one breathes, but **to limit the spread of saliva and nasal droplets that we naturally produce and spread when talking, sneezing, and coughing**.

This eductional campaign has been prepared to help communities learn about droplets and test the usefulness of face coverings. In **four short home experiments** you can quantify and observe how wearing face masks can reduce the spread of droplets to help keep family and friends safe.

This module is based on laboratory studies conducted in a university medical research center. For more information, see:

https://doi.org/10.3389/fmed.2020.00260

Textile Masks and Surface Covers—A Spray Simulation Method and a "Universal Droplet Reduction Model" Against Respiratory Pandemics. Rodriguez-Palacios A, Cominelli F, Basson AR, Pizarro TT, and Ilic S. (2020) Front. Med. 7:260. doi: 10.3389/fmed.2020.00260

https://doi.org/10.3389/fmed.2020.00504

Germ-Free Mice Under Two-Layer Textiles are Fully Protected From Bacteria in Sprayed Microdorplets: A Functional in-vivo Test Strategy of Facemasks and Filtration Materials. Rodriguez-Palacios A, Conger M, and Cominelli F. (2020) Front. Med. 7:504. doi: 10.3389/fmed.2020.00504

When using this international collaboration module please mention, if possible those two studies, the module, & use this **Citation**:

A Citizen Science Facemask Experiment and Educational Modules to Increase Coronavirus Safety in Communities and Schools. Eichler SE, Hopperton AP, Alava JJ, Pereira A, Ahmed R, Kozlakidis Z, Ilic S and Rodriguez-Palacios A (2020) Front. Med. 7:486. doi: 10.3389/fmed.2020.00486

https://doi.org/10.3389/fmed.2020.00486

This Educational Module provides an introduction to the importance of droplets and the value of face covers in preventing the spread of respiratory germs.

With a series of four home experiments you will quantify how face covers help to control respiratory diseases that are transmitted by oral and nasal droplets we produce when we talk, sneeze, or cough.

Experiment 1-How far can sprayed liquid droplets travel?

Experiment 2- How well does a cloth barrier stop sprayed droplets?

Experiment 3- How many germ-carrying droplets can cross two layers of cloth?

Experiment 4- How many germs come out of my mouth as I speak?

...You may share your results and see results from othes here: https://bit.ly/facemaskchallengedata The shared data is anonymous! No personal information is collected!

This Module is also available in **other languages** (*e.g.*, **Spanish**, **French**, **Portuguese**) in the **Citation** and at https://bit.ly/facemaskchallenge (website) & **https://github.com/axr503/education**

If you are a teacher and would like to let us know you that you are implementing the module in your school/class: https://forms.gle/Sg36k3HceMos1Xpb8

Contact: Alex Rodrriguez-P. axr503@case.edu

International collaboration of scientists & educators from

School of Medicine, Case Western Reserve University; University Hospitals; Kent State University; The Ohio State University College of Education and Human Ecology; LMIC; BCNet; International Agency for Research on Cancer/WHO; University at Albany, State University of New York; Universidade Federal do Pará; The University of British Columbia

INTRODUCTION TO CITIZEN SCIENCE ACTIVITY

Citizen Scientists will make simulations with clouds of sprayed droplets using safe household liquids. The project consists of simple home experiments using a spray bottle. Four experiments are described – you can complete one or all of them, using basic food ingredients, kitchen supplies, and a few recycled objects as listed. These activities will consist of measuring how many drops of a sprayed liquid can pass through face cover material, and how far liquid droplets can travel from a simulated sneeze.

Citizen Scientists will learn first-hand about how microdroplets can cause contamination, how face covers work and learn the importance of using a face cover during a pandemic. If you choose to share your data – you will be part of a global science project to help understand diseases and disease prevention – a real Citizen Scientist! And share your results (https://bit.ly/facemaskchallengedata)!

Audience: Recommended for Teachers & Parents of students with 3rd grade reading or above. Suitable for all ages, with supervision as needed.

Basic materials

- 2 cups of dark colored liquid (cooled black coffee, sports drink, grape juice, cola).
- 1 spray bottle.
- 28 pieces letter size white or grid paper.
- Measuring tape or ruler.
- 1 empty cereal box.
- Cloth /textile (at least 10”x10”) (it may get stained!) such as a towel, pillowcase, t-shirt, scarf, bandana, handkerchief, cloth napkin, etc.
- Masking tape.
- Paper towels for cleanup.
- *Optional*: grid paper (see printable or draw your own*).

Materials for Gelatin Germ Growth plates
(advanced, prepare ahead)

- 1 empty cereal box.
- 6-10 shallow clean containers (such as jar lid, tuna can) or *foil* cupcake liners.
- 1 teaspoon sugar.
- 1 cube *beef* broth bouillon.
- 2 packs of ¼ oz. plain (red-colored) gelatin
- 1 cup of water.
- Microwave and 12 oz. glass container, or pot with lid to use on stovetop.
- Clear zip-lock bag.
- Clean face cover (must cover nose and mouth).

https://www.instructables.com/id/Homemade-Nutrient-Agar/

Watch this Nature Video: **The Physics of the Sneeze**, (May 2016, YouTube) to help you understand fluid dynamics.

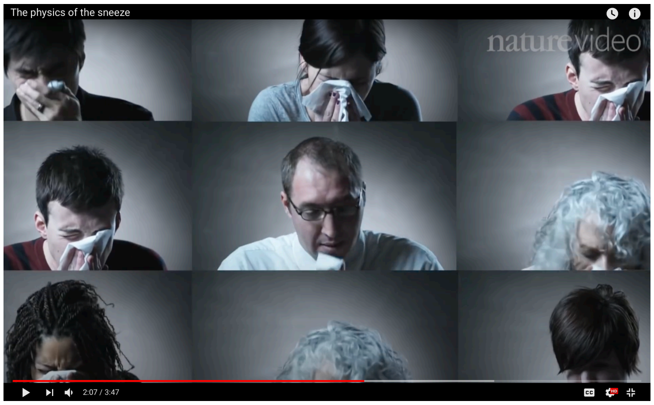

In preparation for experiments, observe a slow motion sneeze (minute 2:00): https://youtu.be/bFxgVkslD-k?t=107

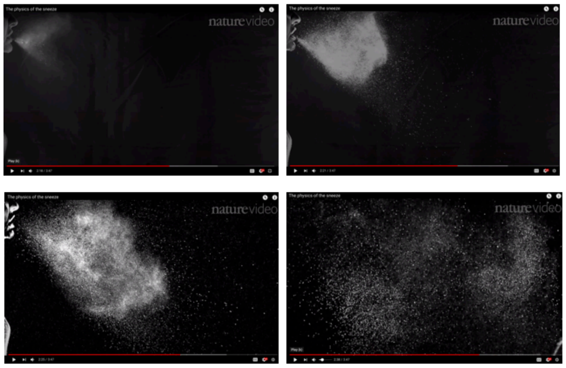

Try to **replicate this droplet cloud with your spray bottle** settings!

EXPERIMENT 1
How far can sprayed liquid droplets travel?

Core Concept: Clouds of sprayed droplets contain multiple droplet sizes: Macro (large, visible) and Micro (small, hard to see) droplets.

Degree of Difficulty: Easy.

New Discovery: Large droplets can travel farther than smaller droplets in a simulated "sneeze".

Learning Objective: Test how far droplets from a simulated infectious spray scenario can spread.

Duration: 20 minutes.

Materials

- 1 spray bottle.
- Enough dark soda, cold coffee, or sports drink to fill a spray bottle halfway.
- 21 pieces of letter sized white or grid paper.
- A large empty space on a table or washable floor
- Pen or pencil.

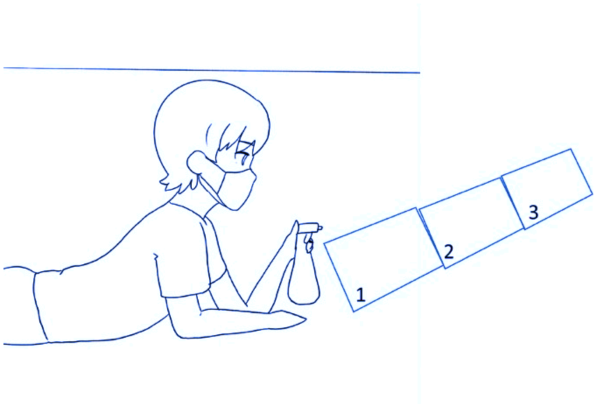

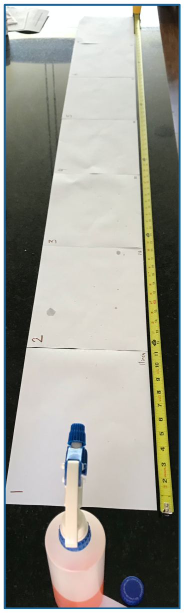

Procedure:

Part A - **Spray Bottle <u>Flat</u> on Floor**:

1. Place 7 pieces of paper aligned on the floor long end to end, number them 1-7 (1 is nearest the spray bottle, 7 is farthest, as shown in diagram/photo).
2. Fill up a spray bottle with any kind of dark, safe liquid such as coffee, grape juice and or cola. Test your spray bottle over a sink and set the spray bottle so that it produces a fine mist with medium sized droplets (see video on page 2 as guide). Once this spray pattern is set do not change it.
3. Position the spray bottle in front of the first paper and aim the spray bottle towards the center of the paper while the bottom of the spray bottle is sitting flat on the floor.
4. Give one complete spray. Wait 30 seconds for droplets to complete their trip, then observe stains on the paper.
5. Record your findings in **Table 1** below (answer questions for '**FLAT** Bottle' on next page, then proceed to **Part B**).

Part B - **Spray Bottle at Different Angles**

1. Start a new experiment by replacing and renumbering the papers just like in Part A.
2. Using the same spray bottle as in Part A, angle the spray bottle with the nozzle ***up*** (to about 10 degrees) using a pencil propped under the bottle. Record your findings in **Table 1** below (see questions for '**Angle UP**', then proceed to next step.
3. Start a new experiment by replacing and renumbering the papers just like in Part A.
4. Complete one full spray. Repeat this same procedure but with the spray bottle angled ***down***, again using a pencil to prop the bottle. Record your findings in **Table 1** below (see questions for '**Angle DOWN**').
5. **You finished Experiment 1!** Go to Table 1 and **share your results online**.

Answer the following Questions in Table 1 below, for each spray experiment

Q1.1 *How would you rate the number of droplets contaminating the third paper*? (see chart below)

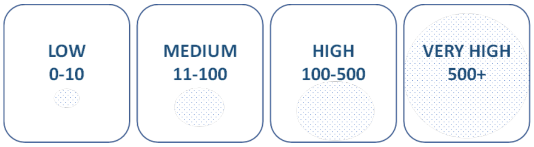

Q1.2 *What bottle position (flat, angled up, or angled down) had the farthest droplets? by how much?*__________

Q1.3 *Were the farthest away droplets large or small?*____________

Table 1. Enter below the Results of Experiment 1.

Bottle Position	***Paper farthest from spray bottle that show droplets (1-7)?***	***Droplet contamination of paper #3 (low, medium, high, very high)?***
FLAT **Bottle**		
**Angled** UP		
**Angled** DOWN		

Share your results online at: https://bit.ly/facemaskchallengedata or scan the QR code below

Take Home Message #FaceMaskChallenge Experiment 1:

- Oral and nasal microdroplets travel pretty far from our mouths!
- If we sneeze with our face upward, droplets spread even farther!
- **Try looking down when you sneeze or cough!**

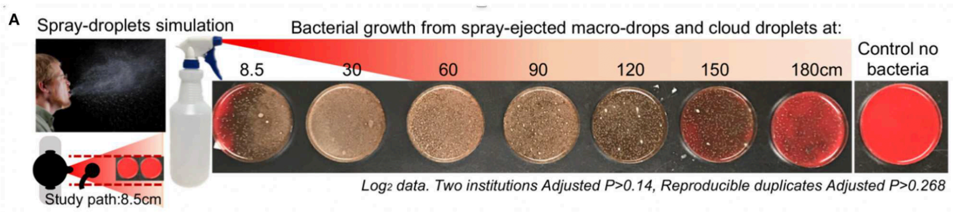

For teachers: This figure above and the scientific details of **Experiments 1 to 3** are described in https://doi.org/10.3389/fmed.2020.00260

Experiment 2
How well does a cloth cover stop sprayed droplets?

Core Concept: Cloth can stop the spread of sprayed liquid droplets).

Degree of Difficulty: Easy.

New Discovery: A single layer of household cloth (cotton t-shirt) reduces the distance of droplet travel by >90%.

Learning Objective: The goal of the experiment is to determine how many macroscopic (visible) droplets get past a face cover and reach a surface.

Duration: 30 minutes.

Materials

- 1 spray bottle.
- Enough dark soda, cold coffee, or sports drink to fill a spray bottle halfway.
- 14 pieces of letter-sized white paper.
- A large empty space on a table or washable floor.
- 1 empty cereal box.
- Scrap cloth/textile about 10" x 10".
- Scissors.
- Silverware, a soup can, or other objects to be used as weights.

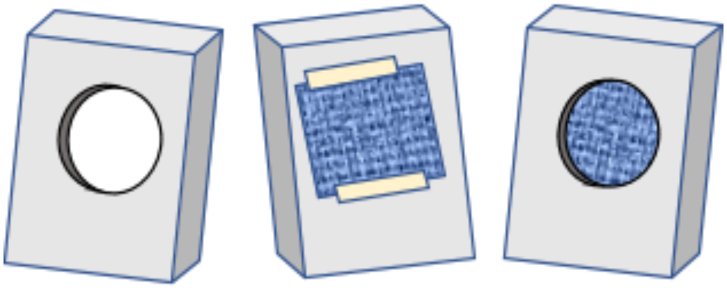

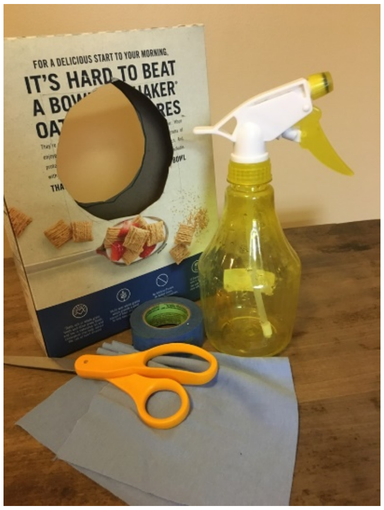

Procedure:

1. Cut 2 approximately 4" diameter windows (or 8X10cm) on both sides of the box, approximately 4 inches from table/floor surface as shown in the pictures above so that the spray bottle nozzle will be at the level of the holes when placed flat on the ground. If the bottle is too tall, slide the box and sheet to edge of table as shown.
2. Tape a single layer of cloth / textile over one of the windows. Or place the fabric you would like to test inside the box covering 'window' as shown in the photo.
3. Secure the box in upright position by placing heavy objects such as utensils, soup can, or other inside.
4. Place 7 pieces of paper on the floor or table long end to end, number them 1-7 as in **Experiment 1** (1 is nearest the spray bottle).
5. Place the box directly in front of paper 1 with no space in between and set the spray bottle flat with the nozzle pointed to spray through the box and through the cloth. If the bottle is too large just angle the bottle so the stream is as close to horizontal as possible.
6. Give one complete spray. Remember to wait 30 seconds to let droplets fall. Observe the spray pattern and answer the questions below. Save the cereal box for use in **Experiment 3**.

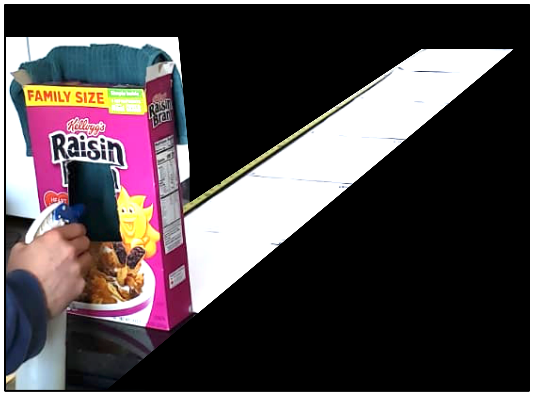

EXPERIMENT 2, continued

Answer the following Questions

Q2.1 What was the farthest paper from the spray bottle on which you could see droplet stains?__________
Q2.2 How would you rate the number of droplets on the third paper?__________

LOW	MEDIUM	HIGH	VERY HIGH
0-10	11-100	100-500	500+

Share your results online at: https://bit.ly/facemaskchallengedata

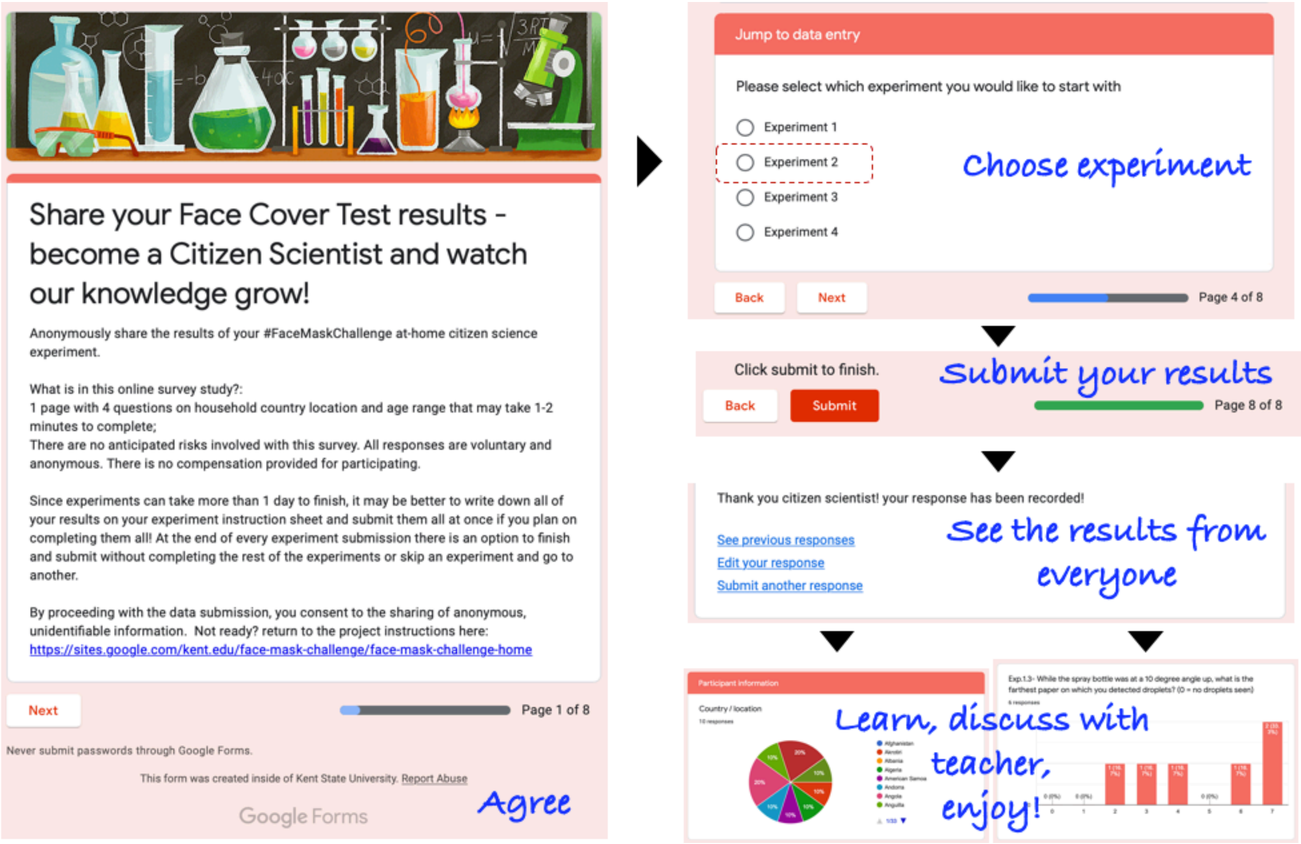

or scan the QR code below

Take Home Message #FaceMaskChallenge Experiment 2:

- Microdroplet spray is reduced by a cloth barrier
- Most macro-droplets are contained by the face cover
- Some droplets get through and land on surfaces so…. **make sure to wash your hands, face covers and avoid touching objects to prevent the spread of illness!**

EXPERIMENT 3
How many germ-carrying droplets can cross cloth?

Core Concept: Sprayed microdroplets may transport bacteria and viruses far from the source.

Degree of Difficulty: Moderate. Hot liquids.

New Discovery: A face mask decreases the transport of droplets carrying bacteria and viruses by >98%.

Learning Objective: By using a spray bottle filled with a "germ solution" (diluted yogurt, soil) and catching the germs on gelatin growth plates, the goal of the experiment is to determine how many macroscopic and microscopic droplets containing germs can cross a face cover (1-layer vs. 2-layers).

Duration: 48-72 hours.

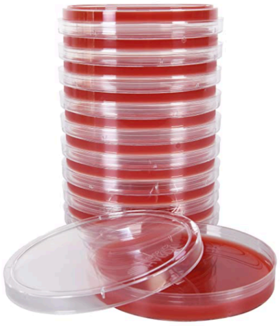

Germ growth plates and materials

(advanced, prepare 1 day ahead)

- *1* spray bottle.
- 1 oz. yogurt, soil, or other cultured food to make a germ-simulating solution.
- 2 empty cereal boxes (reuse box from **Experiment 2**).
- 6 shallow plastic containers at least 2-inch diameter, or *foil* cupcake liners.
- 1 tsp sugar.
- 1 cube beef bouillon (chicken is ok).
- 2 pack plain gelatin, ¼ ounce each.
- 1 cup water.
- Microwave and a 12 oz. glass container, or pot with lid to use on stovetop.
- Clear zip-lock bags.
- Clean face cover.

https://www.instructables.com/id/Homemade-Nutrient-Agar/

You can also purchase ready-to-use culture plates online, where available (e.g., Amazon, Columbia Blood Agar, 5% sheep blood, or tryptic soy agar)

Procedure:

Part A. *The day before* - Prepare the gelatin germ growth plates:

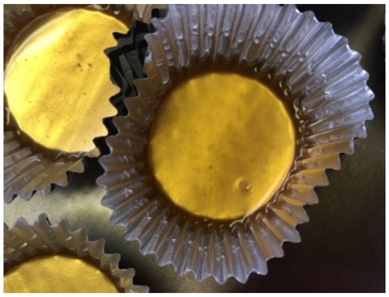

1. Mix 1 cup of water, 1 teaspoon sugar, and 1 beef broth bouillon cube in a microwaveable measuring container in the microwave for 2 minutes. Stir well when done, then heat for 2 more minutes. Leave the mixture inside the microwave to cool about 5 minutes.
2. Alternatively, over medium-low heat bring the water, sugar and bouillon to a low boil while stirring, boil for at least 2 minute. Cover with a tight-fitting lid and turn off heat. Allow the mixture to cool for several minutes.
3. **Put on your face cover and re-wash your hands** to avoid contaminating your plates!
4. Slowly add 2 packets of gelatin powder while stirring.
5. Then **carefully** pour the mixture into shallow round containers (for example, well-cleaned, recycled yogurt container lids) or *foil* cupcake tins about ½" (1cm) deep. You should get at least 6 growth plates. Immediately place poured gelatin plates in a covered container or plastic bag and leave unsealed to allow moisture to escape.
6. Place in a cool location to solidify overnight [a cool oven works well]. Plates must be cool prior to testing. **DO NOT touch the prepared gelatin with your fingers (this could contaminate them!).** Prepare at least 4 gelatin growth plates for Part D. You may want 2 extra plates for each additional type of cloth you want to test. You may wish to prepare 2 more plates for Experiment 4 below. Store in a sealed zip-top bag until use.

Part B. Prepare a germ solution:

1. Add approximately 1 oz. (about 2 tablespoons) of yogurt or soil to about ½ cup warm water and gently mix until dissolved.
2. Put this solution into a clean spray bottle labelled "GERMS." Your solution contains harmless germs that will serve as living indicators to show how a face cloth prevents microscopic drops from spreading after a sneeze.

Part C. Prepare the test boxes:

1. Use the cereal box with windows from Experiment 2. Remove the cloth from the previous experiment and set aside.
2. Prepare a covered container ready to put the gelatin growth plates in, after they are sprayed, such as an uncut cereal box. Alternatively, a large zip-top bag over a clean plastic storage container can be used. This will be your germ-growing container.

Part D. The Experiment (to compare 1-layer vs. 2-layers):

1. While conducting the experiment **wear a FACE COVER** so you do not accidentally contaminate the plates. **DO NOT touch the prepared gelatin with your fingers** (this could contaminate them!).
2. Prepare 4 **gelatin growth plates** to test the effectiveness of cloth face covers. Label the plates as #0: (no cover), #1: (1-layer), #2: (2-layers), and #3: (the face cover you have been using). You will test 2 layers of cloth first.
3. Fold a piece of cloth in half. Cover the box window with **2 layers of cloth** – use masking tape to help keep it in place.
4. Turn the box flat so that the cloth-covered window is facing up. Place plate #2 in the box, under the covered window.
5. From about 5 inches away from the cloth, at about a 45 degree angle, spray TWICE into the window. Wait 30 seconds for microdroplets to land, then carefully slide the plate out of the test box into the germ growing box- be sure not to touch the gelatin!
6. Remove the cloth from the box. Wipe the test box with tissue and dispose of the tissue but save the box for the rest of the experiment.
7. **Repeat steps 2 – 6** using
   a. 1 layer of cloth (plate #1).
   b. your own face cover (plate #3).
   c. no cover (plate #0).
8. Close the germ growing container. Leave it in a warm place where it can stay undisturbed but observed for a few days (on top of the refrigerator works well).

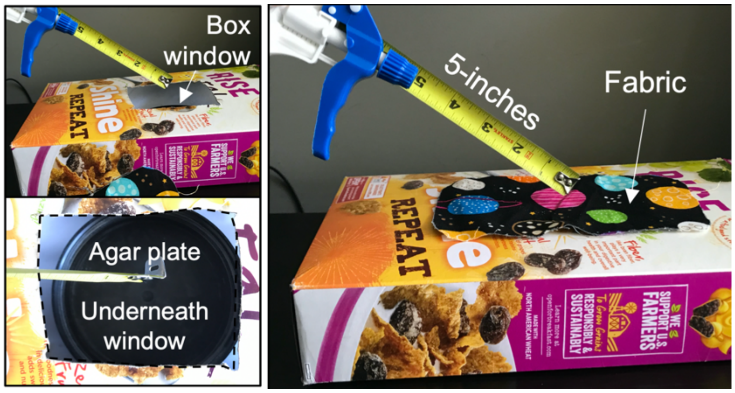

9. Dispose of the box, used cloth, and tissues carefully. Clean the working area and wash your hands thoroughly!
10. Check the gelatin plates after **24-48 hours**. Remember to **wear your cleaned face cover and wash your hands before checking the plates.** Count the number of spots (colony forming units or **CFUs**) that have formed on each plate. If possible, leave the plates in container during observation. Do NOT touch the surface of the plates! You can use the same box for **Experiment 4**.

EXPERIMENT 3, continued

Finish:

1. Record your data in the table below.
2. Wash your hands after observing the plates.
3. Repeat the observation after 48 hours. Record the number of Colony forming units (CFU).
4. Dispose of the germ plates in the trash, wash the area thoroughly and finally re-wash your hands.

Share your data: enter the results of Column G in the form here:
https://docs.google.com/forms/d/e/1FAIpQLSd9cV7HQzxr49MsC-icHCzxIOnlhX2z7e7iza3cJ-NGzJaFRw/viewform

Table 2. Enter below the results of Experiment 3.
CFU: number of colonies on the agar

	A	B	C	D	E	F	G
**Plate**	CFU at 0 hour	CFU at 24 hours	CFU at 48 hours	Diameter of plate in cm	Radius of plate (one half of column D) in cm	Area of plate = pi x r^2= (3.14 x radius x radius) in cm^2	**CFU/cm^2 (column C divided by column F)**
**0**	0						____=Baseline G
**1**	0						
**2**	0						
**3**	0						

Answer the following Questions

Q3.1 What did you observe?

Q3.2 Was one layer of cloth effective in preventing germ transfer?

Q3.3 Were two layers of cloth equally or more effective than one layer?

Q3.4 Can one or two layers of cloth contain all the germs produced by sneezing?

Q3.5 Was your face cover better than no cover at preventing germ transfer?

Share your results online at: https://bit.ly/facemaskchallengedata or scan the QR code below

Take Home Message #FaceMaskChallenge Experiment 3:

- One layer of cloth should help contain germs, but it is not the best
- Two layers of cloth are even better!
- Even with two layers of cloth not every single droplet is trapped in the face covering **so it is very important to wash your hands and practice good hygiene!**
- Since germs in microdroplets are trapped in your face **cover it is important you regularly wash your face covering!**

EXPERIMENT 4
How many germs come out of my mouth as I speak?

Core Concept: We all produce microdroplets containing germs while we speak.

Degree of Difficulty: Moderate.

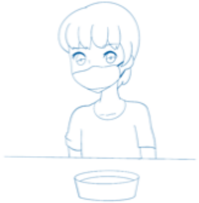

New Discovery: A face cover stops our saliva droplets from contaminating the environment.

Learning Objective: By speaking with or without a face mask and catching the germs on gelatin growth plates, the goal of the experiment is to determine how effectively cloth barriers prevent the spread of oral droplets.

Duration: 48-72 hours.

Materials (advanced, prepare 1 day ahead)

- 2 gelatin germ growth plates (See Experiment 3).
- 1 empty cereal box for incubating germ growth plates.
- Cloth face mask or covering with 2 layers of textile that cover both nose and mouth.
- Or piece of preferred fabric.

Procedure:

Part A. *<u>The day before</u>* - Prepare gelatin germ (or purchase agar) plates – see **Experiment 3** Part A.

Part B. The Experiment (to determine how many droplets we produce when we speak out loud)**:**

1. Download the free CDC Noise App to a smart phone, open it, and adjust laoudness of speech to 70 decibels.
2. Label 2 gelatin growth plates as "#4 openspeech" and "#5 coveredspeech".
3. Prepare a covered container ready to put the plates in after being sprayed. An uncut cereal box would be best. Alternatively, a large zip-top bag over a clean plastic storage container can be used. This will be your incubation container.
4. Sit at a table and place a gelatin growth platemarked "#4 openspeech" on the table about 8 inches away from your mouth.
5. Take a sip of water and read the following passage slowly and clearly as if you were speaking to someone across the room (~70 decibels at arm's length):

"I am a citizen scientist. I am going to read this out loud as an experiment to test the importance of face covers. I want to see how many droplets of saliva I produce that contaminate the environment as I count from 1 to 100. […count out loud to 100]. Thank you and best wishes for good health!"

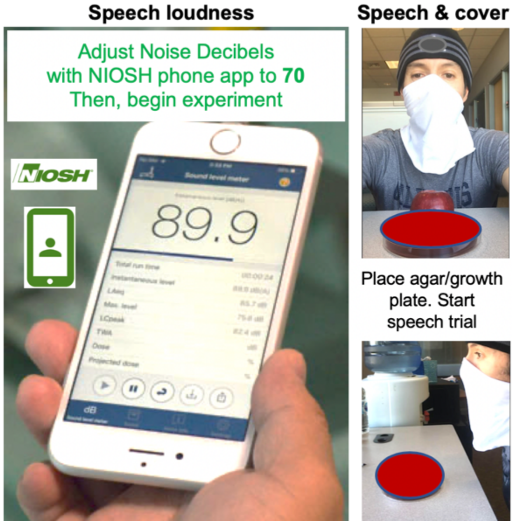

6. Carefully transfer the #openspeech plate to the incubation container.
7. **Cover your face and nose with double layer of cloth**.
8. Sit at a table and place a gelatin growth plate marked "#5 coveredspeech" on the table about 8" away from your mouth.
9. **Repeat** the speech at step 4, above and carefully transfer the #5 coveredspeech plate to the incubation container.
10. Close the incubation container. Leave it in a warm place where it can stay undisturbed but observed for about 2 days (on top of the refrigerator works well).
11. Check the germ plates after 24 hours. Remember to **wear your cleaned face cover and wash your hands before checking the plates**. Count the number of spots (colony forming units or CFUs) that have formed on each plate.

EXPERIMENT 4, continued

Finish:

1. Record your data in the table below.
2. Wash your hands after observing the plates.
3. Repeat the observation after 48 hours. Record the CFU counts.
4. Dispose of the germ plates and the growing box in the trash, wash the area thoroughly and finally re-wash your hands.
5. Share your data: enter the results of Column G in the form here: https://bit.ly/facemaskchallengedata

Table 3. Enter below the Results of Experiment 4

	A	B	C	D	E	F	G
**Plate**	CFU at 0 hour	CFU at 24 hours	CFU at 48 hours	Diameter of plate in cm	Radius of plate (one half of column D) in cm	Area of plate = pi x r^2= (3.14 x radius x radius) in cm^2	**CFU/cm^2 (column C divided by column F)**
**4**	0						____=Baseline G
**5**	0						

Answer the following Questions

*Q4.1 What did you observe?*______________________

*Q4.2 Was a two-layer face cover effective at preventing germ spread?*______________________

*Q4.3 Did the cloth contain all the germs?*______________________

*Q4.4 What other hygiene is necessary for effective germ spread?*______________________

Share results online here: https://bit.ly/facemaskchallengedata or scan the QR code below

Take Home Message #FaceMaskChallenge Experiment 4:

- We spread a lot of invisible germs simply by talking – keep this in mind when you are practicing social distancing
- A part of the solution to COVID or other infections spread by droplet is to wear a face cover and practice careful handwashing and hygiene. Spread the word! Print and post reminder signs:

https://figshare.com/articles/Door_Signs_to_Promote_Public_Droplet_Safety_Amidst_COVID-19/12202808/1

For teachers: The phone App shown in the figure in page 10 and the scientific details of **Experiment 4** are described in https://doi.org/10.3389/fmed.2020.00504

Additional resources

Glossary

Aerosol: a substance that can travel through the air with no liquid. A tiny portion of a human sneeze becomes aerosol.
Colony forming unit (CFU): an estimate of the number of viable (reproducing) bacteria in a sample; a cluster of reproducing bacteria that is large enough to see with an unaided eye.
Droplet: a substance carried in a tiny volume of liquid in which surface tension defines the shape. Most of a human sneeze is droplets.
Epidemic: widespread occurrence of an infectious disease in a community.
Face mask or face cover: any piece of cloth placed over the mouth and nose to prevent spread of respiratory infections.
Medical or surgical mask: personal protective equipment meeting specific manufacturing standards; reduces the spread of infections among healthcare workers and patients.
Pandemic: infectious disease that is prevalent across many communities.
Virus: a particle as small as 20 nanometers that can enter and infect cells. Viruses multiply inside their host's cells using the host's cellular material. Thousands of new virus particles erupt from the cell and escape to new hosts by way of cell fluid.

Links to other resources

Face Mask Challenge Citizen Science module (website)
https://sites.google.com/kent.edu/face-mask-challenge/face-mask-challenge-home
Encyclopedia Britannica: COVID-19 https://www.britannica.com/explore/savingearth/covid-19/
The Scale of Things – Nanoscales https://www.nano.gov/nanotech-101/what/nano-size
Face Mask Challenge Citizen Science Data sharing form
https://docs.google.com/forms/d/e/1FAIpQLSd9cV7HQzxr49MsC-icHCzxIOnlhX2z7e7iza3cJ-NGzJaFRw/viewform
Printable Door sign reminders to encourage wearing face masks
https://figshare.com/articles/Door_Signs_to_Promote_Public_Droplet_Safety_Amidst_COVID-19/12202808/1
NIOSH Sound Level Meter App: https://www.cdc.gov/niosh/topics/noise/app.html

Authors: Sarah E. Eichler[1], Austin P. Hopperton[2], Juan Jose Alava[3], Antonio Jr. Pereira[4], Rukhsana Ahmed[5], Zisis Kozlakidis[6], Sanja Ilic[7], Alex Rodriguez-Palacios[2,8]

Cartoonist: Maddie Rosemark

Affiliations: [1]Department of Biological Sciences, Kent State University, USA; [2]Division of Gastroenterology and Liver Disease, Case Western Reserve University School of Medicine, USA; [3]Institute for the Oceans and Fisheries, The University of British Columbia, Canada; [4]Institute of Technology, Federal University of Pará, Brazil; [5] Department of Communication, University at Albany, SUNY, USA; [6]International Agency for Research on Cancer, World Health Organization, Lyon, France; [7]Department of Human Sciences Human Nutrition, The Ohio State University, USA; [8]University Hospitals Research and Education Institute, University Hospitals Cleveland Medical Center, Cleveland, OH, USA.

Translations: Juan Jose Alava, Diana M. Rodriguez, Diego A. Pulido, Zisis Kozlakidis, Antonio Jr. Pereira, Alex Rodriguez-P & Maissa Zeghidi.

Open CC BY license http://creativecommons.org/licenses/by/4.0/

Versions in four additional languages (Spanish, English, French, Portuguese)are part of the initial submission and are linked via https://sites.google.com/kent.edu/face-mask-challenge/face-mask-challenge-home

Archive - Contact the authors if suggestions/questions. A repository has been created for the archive of the latest post-publication author-approved versions of the module, and for contributions made by the community: **https://github.com/axr503/education**. Alex Rodriguez-Palacios, Assistant Professor, School of Medicine, axr503@case.edu.

International collaboration of scientists & educators from

SCHOOL OF MEDICINE CASE WESTERN RESERVE UNIVERSITY
University Hospitals
Kent State University
UNIVERSIDADE FEDERAL DO PARÁ
THE OHIO STATE UNIVERSITY COLLEGE OF EDUCATION AND HUMAN ECOLOGY
LMIC BCNET
International Agency for Research on Cancer/WHO
UNIVERSITY AT ALBANY State University of New York
THE UNIVERSITY OF BRITISH COLUMBIA

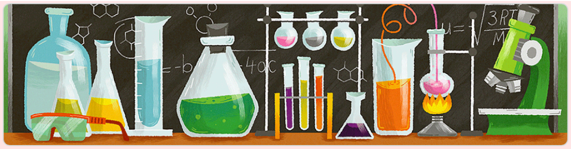

Share your Face Cover Test results - become a Citizen Scientist and watch our knowledge grow!

Anonymously share the results of your #FaceMaskChallenge at-home citizen science experiment.

What is in this online survey study?:
1 page with 4 questions on household country location and age range that may take 1-2 minutes to complete;
There are no anticipated risks involved with this survey. All responses are voluntary and anonymous. There is no compensation provided for participating.

Since experiments can take more than 1 day to finish, it may be better to write down all of your results on your experiment instruction sheet and submit them all at once if you plan on completing them all! At the end of every experiment submission there is an option to finish and submit without completing the rest of the experiments or skip an experiment and go to another.

By proceeding with the data submission, you consent to the sharing of anonymous, unidentifiable information. Not ready? return to the project instructions here:
https://sites.google.com/kent.edu/face-mask-challenge/face-mask-challenge-home

Next — Page 1 of 8

Share your Face Cover Test results - become a Citizen Scientist and watch our knowledge grow!

* Required

Participant information

#FaceCoverChallenge

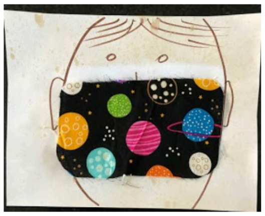

Clicking the 'YES, I Agree' option indicates that you have read the above information, voluntarily agree to participate, and that you are 18 years of age or older. If you do not wish to participate, please click, “NO, I do not wish to participate in the data submission”, or simply close this window. If you agree to participate you will be automatically directed to the data submission form. *

- ◯ Yes, I agree to participate and volunteer information. (click NEXT to proceed)
- ◯ No, I do not wish to participate. (click NEXT to exit)

Is this your first FaceCoverChallenge data submission? *

Choose ▾

Back — Next — Page 2 of 8

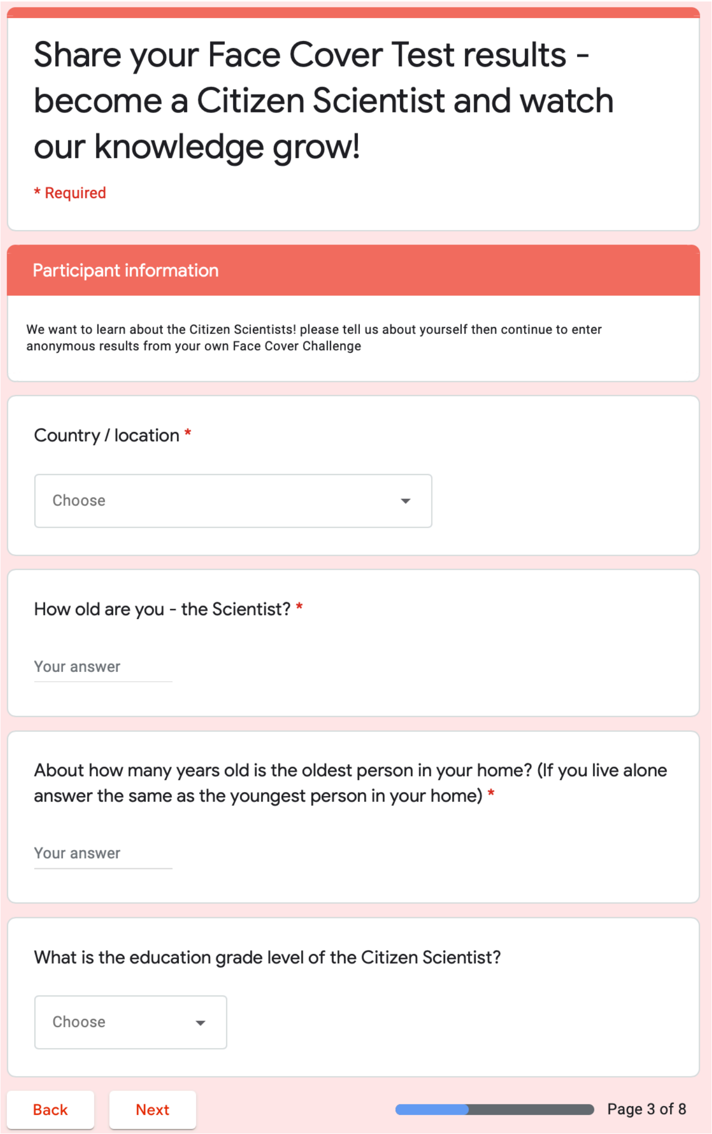

Share your Face Cover Test results - become a Citizen Scientist and watch our knowledge grow!

* Required

Participant information

We want to learn about the Citizen Scientists! please tell us about yourself then continue to enter anonymous results from your own Face Cover Challenge

Country / location *

Choose

How old are you - the Scientist? *

Your answer

About how many years old is the oldest person in your home? (If you live alone answer the same as the youngest person in your home) *

Your answer

What is the education grade level of the Citizen Scientist?

Choose

Back Next

Page 3 of 8

Share your Face Cover Test results - become a Citizen Scientist and watch our knowledge grow!

Jump to data entry

Please select which experiment you would like to start with

- (●) Experiment 1
- ( ) Experiment 2
- ( ) Experiment 3
- ( ) Experiment 4

Clear selection

Back   Next   Page 4 of 8

Share your Face Cover Test results - become a Citizen Scientist and watch our knowledge grow!

Experiment 1 -How far can droplets travel?

Exp.1.1 While the spray bottle was flat what is the farthest paper on which you detected droplets? (0 = no droplets seen)

	0	1	2	3	4	5	6	7	
Closest to Spray Bottle	○	○	○	○	○	○	○	○	Furthest from Spray Bottle

Exp.1.2 - While the spray bottle was flat how would you describe the amount of droplets contaminating the third paper?

- ( ) Low (0-10)
- ( ) Medium (11-100)
- ( ) High (100-500)
- ( ) Very High (500+)

Exp.1.3- While the spray bottle was at a 10 degree angle up, what is the farthest paper on which you detected droplets? (0 = no droplets seen)

	0	1	2	3	4	5	6	7	
Closest to Spray Bottle	○	○	○	○	○	○	○	○	Furthest From Spray Bottle

Exp.1.4 - While the spray bottle was angled 10 degrees angled up how would you describe the amount of droplets contaminating the third paper?

- Low (0-10)
- Medium (11-100)
- High (100-500)
- Very High (500+)

Exp.1.5 - While the spray bottle was at a 10 degree angle down what is the farthest paper on which you detected droplets? (0 = no droplets seen)

	0	1	2	3	4	5	6	7	
Closest to Spray Bottle	○	○	○	○	○	○	○	○	Furthest from spray bottle

Exp.1.6 - While the spray bottle was 10 degrees angled down how would you describe the amount of droplets contaminating the third paper?

- Low (0-10)
- Medium (11-100)
- High (100-500)
- Very High (500+)

Exp.1.7 - What bottle position had the furthest spray?

- Bottle flat
- Bottle angled 10 degrees up
- Bottle angled 10 degrees down
- Other: ____________

Would you like to go on to another experiment or finish and submit?

- (●) Experiment 2
- Experiment 3
- Experiment 4
- Finish and Submit

Clear selection

Back Next Page 5 of 8

Share your Face Cover Test results - become a Citizen Scientist and watch our knowledge grow!

Experiment 2 - How well does a cloth / textile barrier stop droplets?

Exp.2.1 - What type of cloth / textile barrier did you use? (you may select multiple options)

- [ ] Cotton
- [ ] Polyester
- [ ] Cotton / poly mix
- [ ] Silk
- [ ] wool
- [ ] Towel
- [ ] synthetic fiber
- [ ] T-shirt
- [ ] Scarf
- [ ] Other: ______

Exp.2.2 - WITH TEXTILE BARRIER: What is the farthest paper away from the spray source on which you detected droplets? (0 = no droplets seen)

	0	1	2	3	4	5	6	7	
closest to the spray bottle	○	○	○	○	○	○	○	○	furthest from the spray bottle

Exp.2.3 - WITH TEXTILE BARRIER: How would you describe the number of droplets contaminating the third paper?

- ○ Low (0-10)
- ○ Medium (11-100)
- ○ High (100-500)
- ○ Very High (500+)

Would you like to go on to another experiment or finish and submit?

- ○ Experiment 1
- ◉ Experiment 3
- ○ Experiment 4
- ○ Finish and Submit

Clear selection

Back Next

Page 6 of 8

Share your Face Cover Test results - become a Citizen Scientist and watch our knowledge grow!

Experiment 3 - How many germs cross a cloth / textile barrier?

Exp.3- PLATE 0 NO CLOTH / TEXTILE COVER (column G: colonies / cm2)

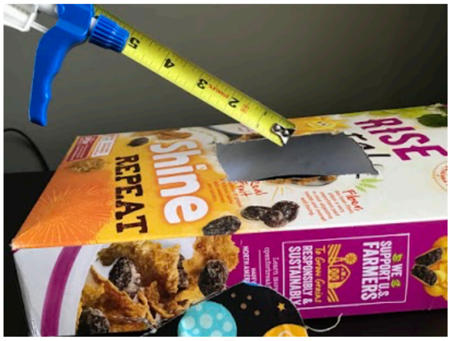

Your answer

Exp.3 - PLATE 1 WITH 1 LAYER OF TEXTILE COVER (column G: colonies / cm2)

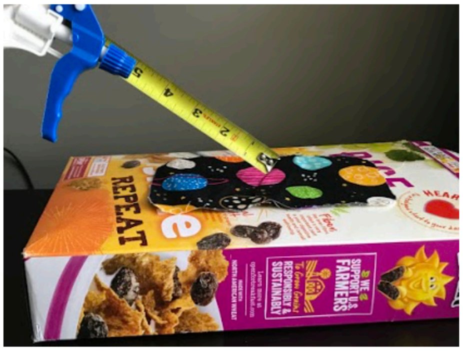

Your answer

Exp.3- PLATE 2 WITH 2 LAYERS OF TEXTILE COVER (column G: colonies / cm2)

Your answer

Exp.3 - PLATE 3 with your own TEXTILE FACE COVER (column G: colonies / cm2)

Your answer

Would you like to go on to another experiment or finish and submit?

- ○ Experiment 1
- ○ Experiment 2
- ◉ Experiment 4
- ○ Finish and Submit

Clear selection

Back Next Page 7 of 8

Share your Face Cover Test results - become a Citizen Scientist and watch our knowledge grow!

Experiment 4 - How many germs come out of my mouth as I speak?

Exp.4 - PLATE 4, NO TEXTILE COVER: Uncovered speech (column G: colonies / cm2)

Your answer

Exp.4 - PLATE 5, WITH TEXTILE FACE COVER: Covered speech (column G: colonies / cm2)

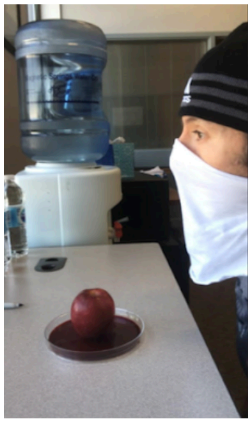

Your answer

Would you like to go back to another experiment or finish and submit?

- ○ Experiment 1
- ○ Experiment 2
- ○ Experiment 4
- ◉ Submit and Finish

Clear selection

Back   Next   Page 8 of 8

Share your Face Cover Test results - become a Citizen Scientist and watch our knowledge grow!

Click submit to finish.

Back   Submit   Page 8 of 8

Share your Face Cover Test results - become a Citizen Scientist and watch our knowledge grow!

Thank you citizen scientist! your response has been recorded!

See previous responses
Edit your response
Submit another response

COLLECTIVE RESULTS

(below is a mock series or random responses for illustration)
Please only enter real results so we all could see the real findings.
If some results do not resemble your data, discuss with a teacher.
And ask/read the real laboratory experiment results that were obtained in a Medical Research Center.

Original studies are published here:

https://doi.org/10.3389/fmed.2020.00260

Textile Masks and Surface Covers—A Spray Simulation Method and a "Universal Droplet Reduction Model" Against Respiratory Pandemics. Rodriguez-Palacios A, Cominelli F, Basson AR, Pizarro TT, and Ilic S. (2020) Front. Med. 7:260. doi: 10.3389/fmed.2020.00260

https://doi.org/10.3389/fmed.2020.00504

Germ-Free Mice Under Two-Layer Textiles are Fully Protected From Bacteria in Sprayed Microdorplets: A Functional in-vivo Test Strategy of Facemasks and Filtration Materials. Rodriguez-Palacios A, Conger M, and Cominelli F. (2020) Front. Med. 7:504. doi: 10.3389/fmed.2020.00504

A Citizen Science Facemask Experiment and Educational Modules to Increase Coronavirus Safety in Communities and Schools. Eichler SE, Hopperton AP, Alava JJ, Pereira A, Ahmed R, Kozlakidis Z, Ilic S and Rodriguez-Palacios A (2020) Front. Med. 7:486. doi: 10.3389/fmed.2020.00486
https://doi.org/10.3389/fmed.2020.00486

Share your Face Cover Test results - become a Citizen Scientist and watch our knowledge grow!

13 responses

Participant information

Clicking the 'YES, I Agree' option indicates that you have read the above information, voluntarily agree to participate, and that you are 18 years of age or older. If you do not wish to participate, please click, "NO, I do not wish to participate in the data submission", or simply close this window. If you agree to participate you will be automatically directed to the data submission form.

12 responses

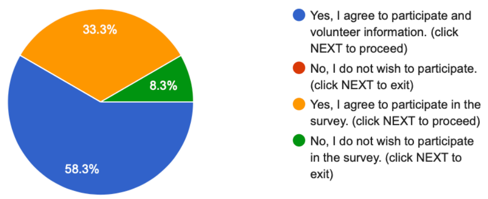

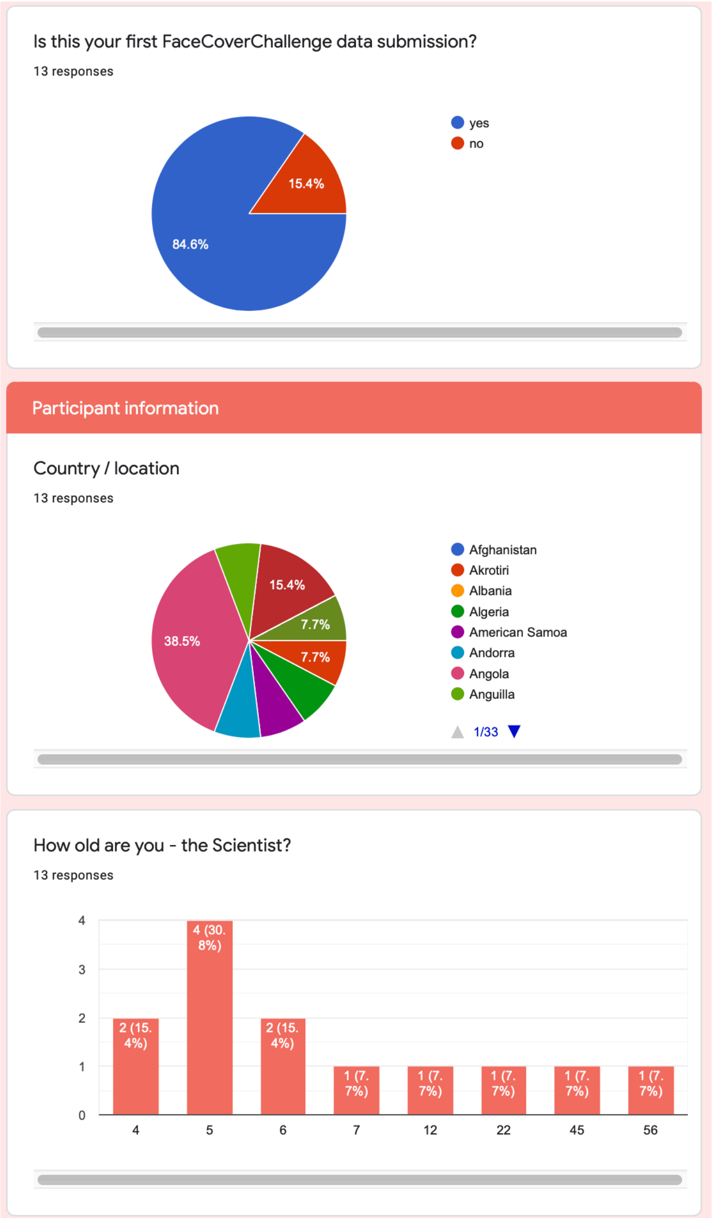
Is this your first FaceCoverChallenge data submission?
13 responses
yes
no
15.4%
84.6%
Participant information
Country / location
13 responses
15.4%
7.7%
38.5%
7.7%
Afghanistan
Akrotiri
Albania
Algeria
American Samoa
Andorra
Angola
Anguilla
1/33
How old are you - the Scientist?
13 responses
4
3
2
1
0
2 (15.4%)
4 (30.8%)
2 (15.4%)
1 (7.7%)
1 (7.7%)
1 (7.7%)
1 (7.7%)
1 (7.7%)
4
5
6
7
12
22
45
56

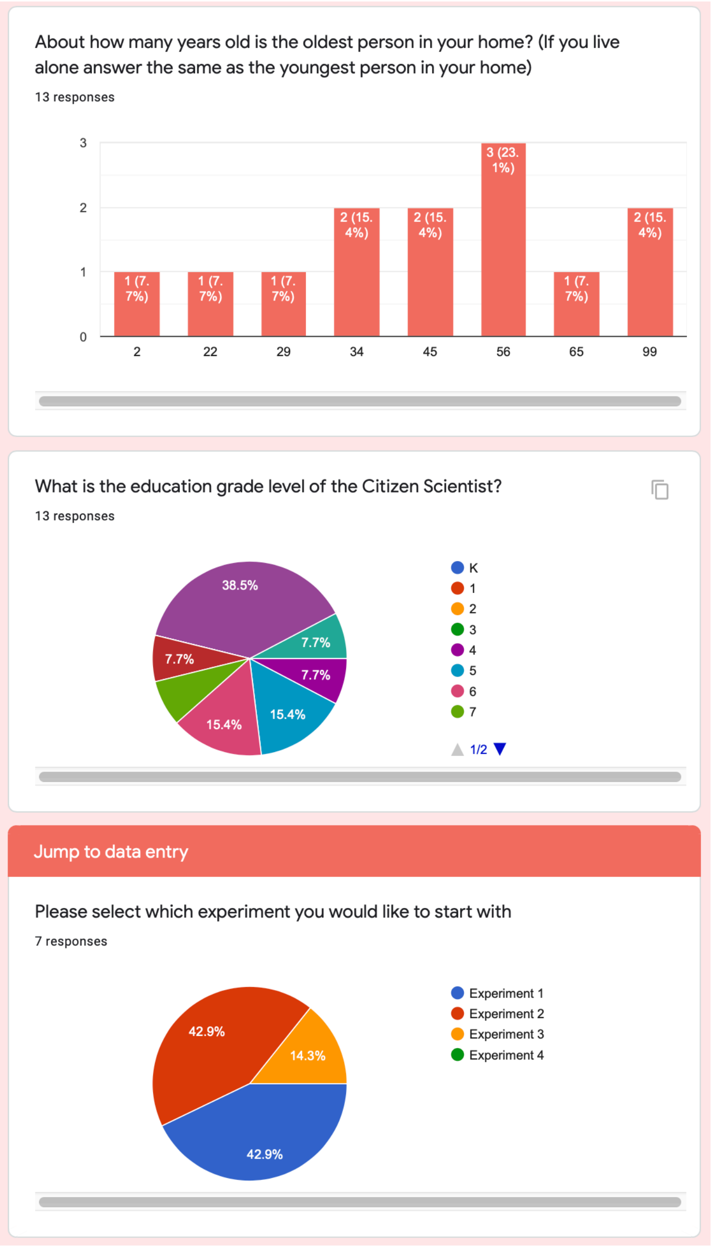

See remaining results in response form link

https://docs.google.com/forms/d/e/1FAIpQLSd9cV7HQzxr49MsC-icHCzxIOnlhX2z7e7iza3cJ-NGzJaFRw/viewform

A Citizen Science Facemask Experiment and Educational Modules to Increase Coronavirus Safety in Communities and Schools. Eichler SE, Hopperton AP, Alava JJ, Pereira A, Ahmed R, Kozlakidis Z, Ilic S and Rodriguez-Palacios A (2020) Front. Med. 7:486. doi: 10.3389/fmed.2020.00486

SUPPLEMENTARY MODULE - In English.

Provided by Alex Rodriguez-P. axr503@case.edu.

V1 (August 20, 2020) with the accepted publication.

Results submission form in English:

https://docs.google.com/forms/d/e/1FAIpQLSd9cV7HQzxr49MsC-icHCzxIOnlhX2z7e7iza3cJ-NGzJaFRw/viewform

DATA SUBMISSION FORMS in:

English: https://bit.ly/facemaskchallengedata

Français: https://forms.gle/5q7V44nbT8fu6Jvz6

Espanol: https://forms.gle/iWgE8JFeDvezxXTq6

Portugues: https://forms.gle/EJHauCWTdDbny66N8

frontiers in Medicine

OPINION
published: xx July 2020
doi: 10.3389/fmed.2020.00486

Check for updates

A Citizen Science Facemask Experiment and Educational Modules to Improve Coronavirus Safety in Communities and Schools

*Sarah E. Eichler[1], Austin P. Hopperton[2], Juan José Alava[3], Antonio Pereira Jr.[4], Rukhsana Ahmed[5], Zisis Kozlakidis[6], Sanja Ilic[7] and Alexander Rodriguez-Palacios[2]**

[1] Department of Biological Sciences, Kent State University at Salem, Salem, MA, United States, [2] Division of Gastroenterology and Liver Disease, School of Medicine, Case Western Reserve University, Cleveland, OH, United States, [3] Institute for Oceans and Fisheries, University of British Columbia, Vancouver, BC, Canada, [4] Institute of Technology, Federal University of Pará, Belém, Brazil, [5] Department of Communication, University at Albany, SUNY, Albany, NY, United States, [6] International Agency for Research on Cancer, World Health Organization (France), Lyon, France, [7] Department of Human Sciences, The Ohio State University, Columbus, OH, United States

Keywords: COVID-19, mass media, health communication, prevention, intervention, social behavioral changes, facemask, school education

OPEN ACCESS

SUPPLEMENTARY MATERIALS

Education Module #1 ENGLISH
Education Module #2 SPANISH
Education Module #3 FRENCH
Education Module #4 PORTUGUESE

Education Module

SPANISH

Experimento de Ciencia Ciudana y Mascarillas

Campaña Educativa y Módulos para Promover la Seguridad contra Coronavirus en Comunidades y Escuelas

COVID-19 es una enfermedad causada por un **coronavirus que se transmite principalmente a través de gotas** de fluidos orales y nasales. El COVID-19 afecta con mayor gravedad a los ancianos y a los que ya están enfermos, provocando dificultad para respirar, fiebre, tos, dolores musculares e inflamación sistémica que puede llevar a la hospitalización.

COVID se transmite de persona a persona a través de la saliva y microgotas nasales. Debido a esto, los expertos en salud recomiendan mantener una distancia de 6 pies (2 m) de otras personas, limitar la exposición en grupos o no congregarse en grandes grupos, lavarse las manos y usar macaras. Dado que las mascaras son escasas en algunos lugares,muchas personas han estado usando cubiertas para la cara, como bufandas y pañuelos. La función principal de las máscaras/mascarillas y las cubiertas faciales no es filtrar el aire que se respira, sino **limitar la propagación de la saliva y las microgotas nasales que producimos y propagamos de forma natural al hablar, estornudar y toser**.

Esta campaña educativa ha sido preparada para ayudar a las comunidades a probar y validar la utilidad de las cubiertas o revetsimientos faciales. **En cuatro experimentos cortos** en el hogar, usted puede cuantificar y observar cómo el uso de cubiertas para la cara puede reducir la propagación de gotitas (droplets) para mantenerse a salvo y proteger a familiares y amigos.

Este módulo se basa en estudios de laboratorio realizados en un centro universitario de investigación médica. Para más información:

https://doi.org/10.3389/fmed.2020.00260

Textile Masks and Surface Covers—A Spray Simulation Method and a "Universal Droplet Reduction Model" Against Respiratory Pandemics. Rodriguez-Palacios A, Cominelli F, Basson AR, Pizarro TT, and Ilic S. (2020) Front. Med. 7:260. doi: 10.3389/fmed.2020.00260

https://doi.org/10.3389/fmed.2020.00504

Germ-Free Mice Under Two-Layer Textiles are Fully Protected From Bacteria in Sprayed Microdorplets: A Functional in-vivo Test Strategy of Facemasks and Filtration Materials. Rodriguez-Palacios A, Conger M, and Cominelli F. (2020) Front. Med. 7:504. doi: 10.3389/fmed.2020.00504

Cuando utilice este módulo de colaboración internacional, mencione, si es posible, esos dos estudios, el módulo y utilice esta cita:

A Citizen Science Facemask Experiment and Educational Modules to Increase Coronavirus Safety in Communities and Schools. Eichler SE, Hopperton AP, Alava JJ, Pereira A, Ahmed R, Kozlakidis Z, Ilic S and Rodriguez-Palacios A (2020) Front. Med. 7:486. doi: 10.3389/fmed.2020.00486
https://doi.org/10.3389/fmed.2020.00486

Este módulo educativo proporciona una introducción a la importancia de las gotitas ("droplets") y el valor de las cubiertas faciales para prevenir la propagación de gérmenes respiratorios.

Con una serie de cuatro experimentos caseros cuantificarás cómo las fundas faciales ayudan a controlar las enfermedades respiratorias que se transmiten por las gotitas orales y nasales que producimos al hablar, estornudar o toser.

Experimento 1-¿Qué tan lejos pueden viajar las gotas/gotitas de líquido rociadas (droplets)?
Experimento 2- ¿Qué tan bien una barrera de tela detiene las gotas/gotitas rociadas?
Experimento 3- ¿Cuántas gotas con gérmenes pueden atravesar dos capas de tela?
Experimento 4- ¿Cuántos gérmenes salen de mi boca mientras hablo?

Usted puede compartir los resultados de sus experimentos, y ver los resultados de otros aquí, en **Ingles** https://bit.ly/facemaskchallengedata o en **Espanol**: https://forms.gle/iWgE8JFeDvezxXTq6

¡Los datos compartidos son anónimos! ¡No se recopila información personal!

Este módulo también está disponible en **otros idiomas** (i.e,, **Inglés**, **Francés**, **Portugués**) en el **Articulo Scientifico** y en https://bit.ly/facemaskchallenge (website) & **https://github.com/axr503/education**

Si usted es professor y le gustaria dedejarnos saber que existe interes o estan implimentando este modulo en su escuela o clase: https://forms.gle/Sg36k3HceMos1Xpb8

Contacto: Alex Rodriguez-P. axr503@case.edu

International collaboration of scientists & educators from

School of Medicine Case Western Reserve University; University Hospitals; Kent State University; The Ohio State University College of Education and Human Ecology; LMIC BCNet; International Agency for Research on Cancer/WHO; University at Albany State University of New York; Universidade Federal do Pará; The University of British Columbia

INTRODUCCIÓN A LA ACTIVIDAD DE CIENCIA CIUDADANA

Los ciudadanos cientificos harán simulaciones con nubes de gotitas rociadas utilizando líquidos domésticos seguros. El proyecto consiste en sencillos experimentos caseros con un atomizador. Se describen cuatro experimentos: puede completar uno o todos ellos, utilizando ingredientes alimentarios básicos, suministros de cocina y algunos objetos reciclados que se enumeran. Estas actividades consistirán en medir cuántas gotas de un líquido rociado pueden pasar a través del material de la cubierta facial y qué tan lejos pueden viajar las gotas de líquido desde un estornudo simulado.

Los científicos ciudadanos aprenderán de primera mano cómo las microgotas pueden causar contaminación, cómo funcionan las cubiertas faciales y aprenderán la importancia de usar una cubierta facial durante una pandemia. Si eliges compartir tus datos, serás parte de un proyecto científico global para ayudar a comprender las enfermedades y la prevención de enfermedades, ¡un verdadero científico ciudadano! Y comparte tus resultados en Ingles (https://bit.ly/facemaskchallengedata) o en Espanol https://forms.gle/iWgE8JFeDvezxXTq6!

Audiencia: Recomendado para maestros y padres de estudiantes con lectura de tercer grado o superior. Apto para todas las edades, con supervisión según sea necesario.

Materiales basicos

- 2 tazas de líquido de color oscuro (café negro, bebida deportiva, jugo de uva, cola).
- 1 botella de spray.
- 28 piezas de papel tamaño carta blanco o cuadriculado.
- Cinta métrica o regla.
- 1 caja de cereal vacía.
- Paño / tela (al menos 10 "x 10") (¡puede mancharse!) Como una toalla, funda de almohada, camiseta, bufanda, pañuelo, servilleta de tela, etc.
- Cinta adhesiva.
- Toallas de papel para limpieza.
- *Opcional*: papel cuadriculado (ver imprimible o dibujar el tuyo *).

Materiales para placas de crecimiento de gérmenes de gelatina
(avanzado, prepárate)

- 1 caja de cereal vacía.
- 6-10 recipientes limpios poco profundos (como tapa de frasco, lata de atún) o forros de aluminio para cupcakes.
- 1 cucharadita de azúcar.
- 1 cubo de caldo de res.
- 2 paquetes o ¼ oz. gelatina normal (de color rojo)
- 1 taza de agua.
- Microondas y 12 oz. recipiente de vidrio o una olla con tapa para usar en la estufa.
- Bolsa transparente con cierre de cremallera.
- Cubierta facial limpia (debe cubrir la nariz y la boca).

https://www.instructables.com/id/Homemade-Nutrient-Agar/

Mire este video de Nature: **La física del estornudo**, (mayo de 2016, YouTube) para ayudarlo a comprender la dinámica de fluidos.(En Ingles)

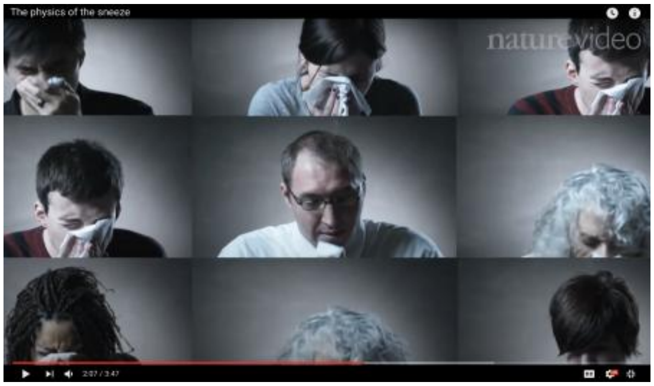

En preparación para los experimentos, observe un estornudo en cámara lenta (minuto 2:00): https://youtu.be/bFxgVkslD-k?t=107

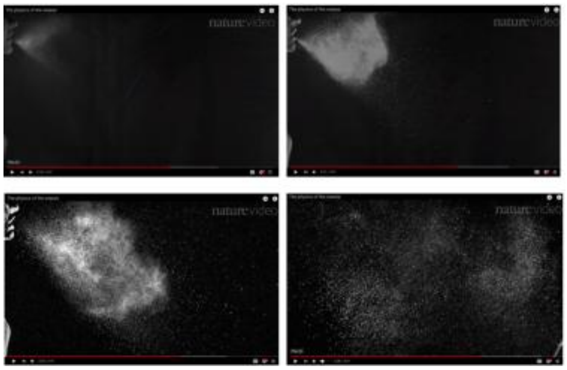

¡Intenta repetir esta nube de gotas con la configuración de su botella de spray!

EXPERIMENTO 1
¿Qué tan lejos pueden viajar las gotas de líquido rociadas?

Concepto principal: Las nubes de gotas rociadas contienen múltiples tamaños de gotas: gotas macro (grandes, visibles) y micro (pequeñas, difíciles de ver).

Grado de dificultad: Fácil.

Nuevo descubrimiento: Las gotas grandes pueden viajar más lejos que las gotas más pequeñas en un "estornudo" simulado.

Objetivo de aprendizaje: Pruebe qué tan lejos pueden propagarse las gotas de un escenario de rociado infeccioso simulado.

Duración: 20 minutos.

Materiales

- 1 botella de spray.
- Suficiente refresco oscuro, café frío o bebida deportiva para llenar una botella con atomizador hasta la mitad.
- 21 piezas de papel blanco o cuadriculado tamaño carta.
- Un gran espacio vacío en una mesa o piso lavable.
- Bolígrafo o lápiz.

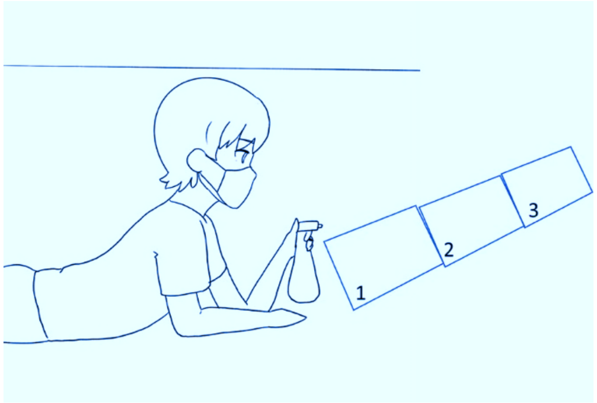

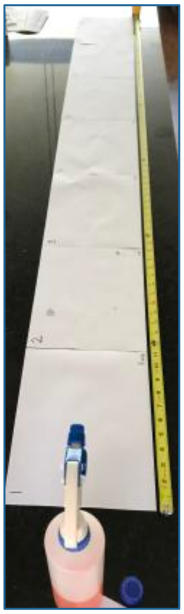

Procedimiento

Parte A - Botella de spray plana en el suelo:

1. Coloque 7 hojas de papel alineadas en el piso de un extremo a otro, numere del 1 al 7 (1 es el más cercano a la botella rociadora, 7 es el más alejado, como se muestra en el diagrama / foto).
2. Llene una botella con atomizador con cualquier tipo de líquido oscuro y seguro, como café, jugo de uva o cola. Pruebe su botella rociadora sobre un fregadero y coloque la botella rociadora de modo que produzca una fina niebla con gotas de tamaño mediano (vea el video en la página 2 como guía). Una vez establecido este patrón de pulverización, no lo cambie.
3. Coloque la botella rociadora frente al primer papel y apunte la botella rociadora hacia el centro del papel mientras la parte inferior de la botella rociadora está apoyada en el suelo.
4. Dar una pulverización completa. Espere 30 segundos para que las gotas completen su viaje, luego observe las manchas en el papel.
5. Registre sus hallazgos en la **Tabla 1** a continuación (responda las preguntas para 'Botella PLANA' en la página siguiente, luego continúe con la **Parte B**).

EXPERIMENTO 1, continuación

Parte B - Botella de spray en diferentes ángulos

1. Inicie un nuevo experimento reemplazando y volviendo a numerar los papeles como en la Parte A.
2. Usando la misma botella rociadora que en la Parte A, incline la botella rociadora con la boquilla hacia arriba (a unos 10 grados) usando un lápiz colocado debajo de la botella. Registre sus hallazgos en la Tabla 1 a continuación (vea las preguntas para 'Ángulo ARRIBA', luego continúe con el siguiente paso.
3. Inicie un nuevo experimento reemplazando y volviendo a numerar los papeles como en la Parte A.
4. Complete una pulverización completa. Repita este mismo procedimiento pero con la botella de spray en ángulo hacia abajo, nuevamente usando un lápiz para sostener la botella. Registre sus hallazgos en la Tabla 1 a continuación (vea las preguntas para 'Ángulo ABAJO').
5. **¡Terminaste el Experimento 1!**Vaya a la Tabla 1 y comparta sus resultados en línea.

Responda las siguientes preguntas en la Tabla 1 a continuación, para cada experimento de pulverización

Q1.1 *¿Cómo calificaría la cantidad de gotas que contaminan el tercer papel?*? (ver cuadro a continuación)

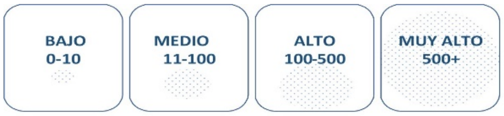

Q1.2 *¿Qué posición de la botella (plana, inclinada hacia arriba o hacia abajo) tenía las gotas más lejanas? ¿Por cuanto?*__________

Q1.3 *¿Las gotas más lejanas eran grandes o pequeñas?* _____________

Tabla 1. Ingrese a continuación los Resultados del Experimento 1.

Posición de la botella	***¿Papel más alejado de la botella de spray que muestra gotas (1-7)?***	***Contaminación del papel por gotitas # 3 (bajo, medio, alto, muy alto)?***
PLANO **Botella**		
**En ángulo hacia arriba**		
**Ángulo ABAJO**		

Comparta sus resultados Ingles (https://bit.ly/facemaskchallengedata) o Espanol https://forms.gle/iWgE8JFeDvezxXTq6!

O escanee el código QR a continuación

Mensaje para llevar a casa #FaceMaskChallenge Experiment 1:

- ¡Las microgotas orales y nasales viajan bastante lejos de nuestra boca!
- Si estornudamos con la cara hacia arriba, ¡las gotas se esparcen aún más!
- **¡Intente mirar hacia abajo cuando estornude o tosa!**

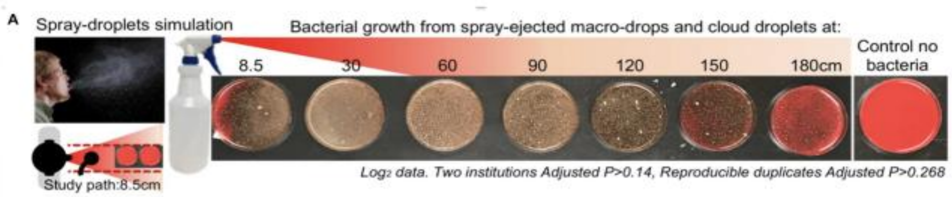

Para profesores: Esta figura anterior y los detalles científicos de los Experimentos 1 a 3 se describen en https://doi.org/10.3389/fmed.2020.00260

Experimento 2 ¿Qué tan bien una cubierta de tela detiene las gotas/gotitas rociadas?

Concepto principal: El paño puede detener la propagación de las gotas de líquido rociadas).

Grado de dificultad: Fácil.

Nuevo descubrimiento: Una sola capa de tela para el hogar (camiseta de algodón) reduce la distancia de recorrido de las gotas en> 90%.

Objetivo de aprendizaje: El objetivo del experimento es determinar cuántas gotas macroscópicas (visibles) atraviesan una cubierta facial y alcanzan una superficie.

Duración: 30 minutos.

Materiales

- 1 botella de spray.
- Suficiente refresco oscuro, café frío o bebida deportiva para llenar una botella con atomizador hasta la mitad.
- 14 hojas de papel blanco tamaño carta.
- Un gran espacio vacío sobre una mesa o suelo lavable.
- 1 caja de cereal vacía.
- Tela / tela de desecho de aproximadamente 10 "x 10".
- Tijeras.
- Cubiertos, una lata de sopa u otros objetos para usar como pesas.

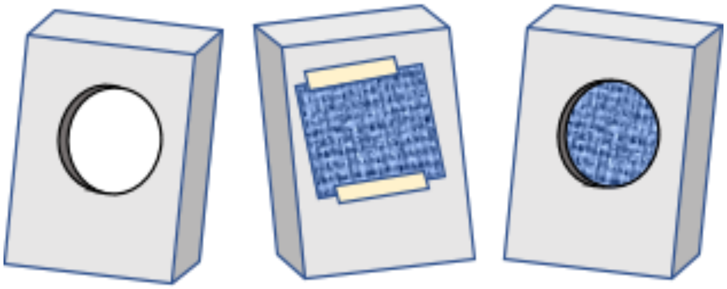

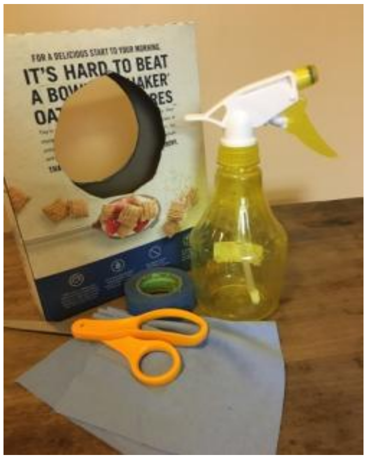

Procedimiento

1. Corte 2 ventanas de aproximadamente 4 ”de diámetro (o 8 x 10 cm) a ambos lados de la caja, aproximadamente a 4 pulgadas de la superficie de la mesa / piso como se muestra en las imágenes de arriba para que la boquilla de la botella rociadora esté al nivel de los orificios cuando se coloque plana el terreno. Si la botella es demasiado alta, deslice la caja y la hoja hasta el borde de la mesa como se muestra.

2. Pega una sola capa de tela / textil sobre una de las ventanas. O coloque la tela que le gustaría probar dentro de la caja que cubre la 'ventana' como se muestra en la foto.

3. Asegure la caja en posición vertical colocando objetos pesados como utensilios, latas de sopa u otros dentro.

4. Coloque 7 hojas de papel en el suelo o en la mesa de un extremo a otro, numere del 1 al 7 como en el Experimento 1 (1 es el más cercano a la botella de spray).

5. Coloque la caja directamente frente al papel 1 sin espacio entre ellos y coloque la botella de spray en posición plana con la boquilla apuntando para rociar a través de la caja y a través del paño. Si la botella es demasiado grande, simplemente incline la botella para que el chorro esté lo más cerca posible de la horizontal.

6. Dar una pulverización completa. Recuerde esperar 30 segundos para dejar caer las gotas. Observe el patrón de pulverización y responda las siguientes preguntas. Guarde la caja de cereal para usarla en el **Experimento 3.**

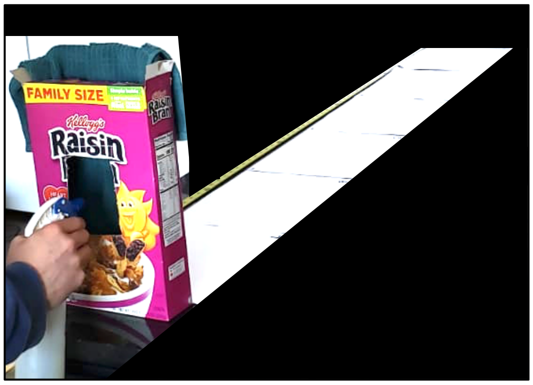

Responde las siguientes preguntas

Q2.1 ¿Cuál fue el papel más alejado de la botella de spray en el que pudo ver manchas de gotas? __________

Q2.2 ¿Cómo calificaría la cantidad de gotas en el tercer papel? __________

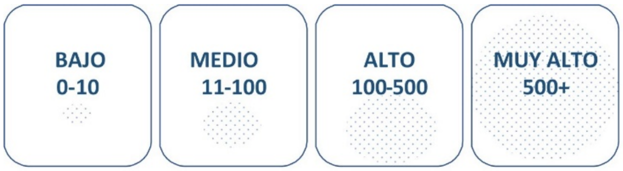

Comparta sus resultados Ingles (https://bit.ly/facemaskchallengedata) o Espanol https://forms.gle/iWgE8JFeDvezxXTq6!
o escanee el código QR abajo

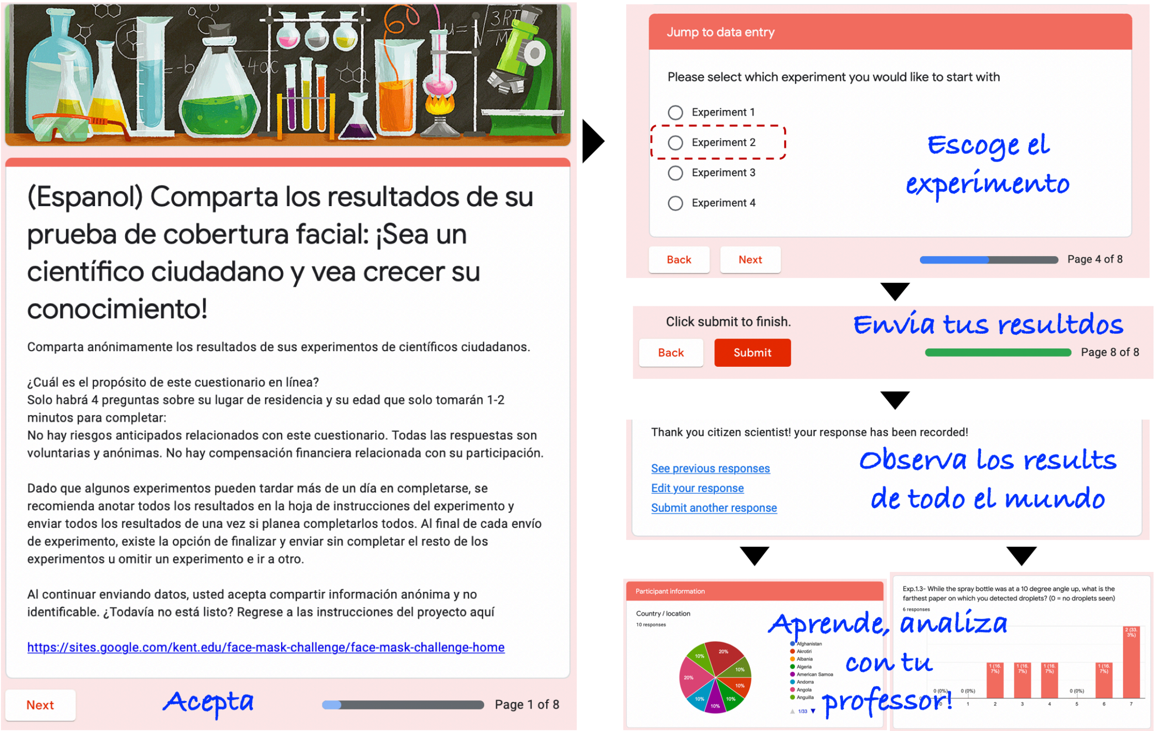

Mensaje para casa #FaceMaskChallenge Experimento 2:

- El spray de microgotas se reduce mediante una barrera de tela
  Las microgotas son portadoras de germenes.
- La mayoríade las macrogotas están contenidas en la cubierta facial
- Algunas gotas atraviesan y aterrizan en superficies así que…. ¡asegúrese de lavarse las manos, cubrirse la cara y evitar tocar objetos para evitar la propagación de enfermedades!

EXPERIMENTO 3 ¿Cuántas gotas/gotitas portadoras de gérmenes pueden atravesar la tela?

Concepto principal: Las microgotas rociadas pueden transportar bacterias y virus lejos de la fuente.

Grado de dificultad: Moderar. Líquidos calientes.

Nuevo descubrimiento: Una mascarilla reduce el transporte de gotitas portadoras de bacterias y virus en> 98%.

Objetivo de aprendizaje: Al usar una botella rociadora llena de una "solución de gérmenes" (yogur diluido, tierra) y atrapar los gérmenes en las placas de crecimiento de gelatina, el objetivo del experimento es determinar cuántas gotas macroscópicas y microscópicas que contienen gérmenes pueden atravesar una cubierta facial (1 -capa frente a 2 capas).

Duración: 48-72 horas.

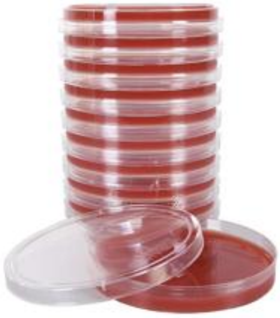

Placas y materiales de crecimiento de gérmenes (avanzado, preparar con 1 día de anticipación)

- 1 botella de spray.
- 1 onza. yogur, tierra u otros alimentos cultivados para hacer una solución de simulación de gérmenes.
- 2 cajas de cereal vacías (caja de reutilización del Experimento 2).
- 6 recipientes de plástico poco profundos de al menos 2 pulgadas de diámetro o forros de aluminio para cupcakes.
- 1 cucharadita de azúcar.
- 1 cubo de caldo de res (el pollo está bien).
- 2 paquetes de gelatina natural de ¼ de onza cada uno.
- 1 taza de agua.
- Microondas y 12 oz. recipiente de vidrio o una olla con tapa para usar en la estufa.
- Bolsas transparentes con cierre hermético.
- Limpiar la cubierta facial.

https://www.instructables.com/id/Homemade-Nutrient-Agar/

También puede comprar placas de cultivo listas para usar en línea, donde estén disponibles (por ejemplo, Amazon, Columbia Blood Agar, 5% sangre de ovejao agar de soja tríptico)

Procedimiento:

Parte A. El día anterior - Prepare las placas de gelatina de crecimiento de germen:

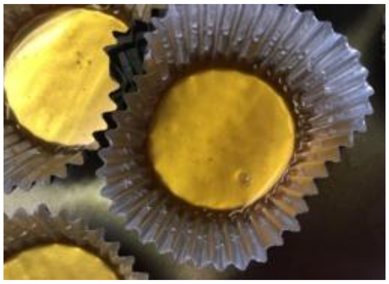

1. Mezcle 1 taza de agua, 1 cucharadita de azúcar y 1 cubo de caldo de res en un recipiente medidor apto para microondas en el microondas durante 2 minutos. Revuelva bien cuando esté listo, luego caliente por 2 minutos más. Deje que la mezcla dentro del microondas se enfríe unos 5 minutos.
2. Alternativamente, a fuego medio-bajo, lleve el agua, el azúcar y el caldo a ebullición baja mientras revuelve, hierva durante al menos 2 minutos. Cubra con una tapa hermética y apague el fuego. Deje que la mezcla se enfríe durante varios minutos.
3. **Póngase la cubierta facial y vuelva a lavarse las manos.** para evitar contaminar tus platos!
4. Agregue lentamente 2 paquetes de gelatina en polvo mientras revuelve.
5. Luego, vierta con cuidado la mezcla en recipientes redondos poco profundos (por ejemplo, tapas de recipientes de yogur reciclado bien limpias) o en latas de aluminio para cupcakes de aproximadamente ½ ”(1 cm) de profundidad. Debes obtener al menos 6 placas de crecimiento. Coloque inmediatamente las placas de gelatina vertidas en un recipiente cubierto o en una bolsa de plástico y déjelas sin sellar para permitir que escape la humedad.
6. Coloque en un lugar fresco para solidificar durante la noche [un horno frío funciona bien]. Las placas deben estar frías antes de la prueba. NO toque la gelatina preparada con los dedos (¡esto podría contaminarlos!). Prepare al menos 4 placas de crecimiento de gelatina para la Parte D. Es posible que desee 2 placas adicionales por cada tipo adicional de tela que desee probar. Es posible que desee preparar 2 platos más para el Experimento 4 a continuación. Almacene en una bolsa sellada con cierre hermético hasta su uso.

EXPERIMENTO 3, continuación

Parte B. Prepare una solución de gérmenes:

1. Agregue aproximadamente 1 oz. (aproximadamente 2 cucharadas) de yogur o tierra en aproximadamente ½ taza de agua tibia y mezcle suavemente hasta que se disuelva.

2. Ponga esta solución en una botella de spray limpia con la etiqueta "GÉRMENES". Su solución contiene gérmenes inofensivos que servirán como indicadores vivos para mostrar cómo un paño facial evita que las gotas microscópicas se propaguen después de un estornudo.

Parte C. Prepare las cajas de prueba:

1. Use la caja de cereal con ventanas del Experimento 2. Retire la tela del experimento anterior y déjela a un lado.

2. Prepare un recipiente tapado listo para colocar las placas de crecimiento de gelatina, después de rociarlas, como una caja de cereal sin cortar. Alternativamente, se puede usar una bolsa grande con cierre de cremallera sobre un recipiente de almacenamiento de plástico limpio. Este será su contenedor de cultivo de gérmenes.

Parte D. El experimento (para comparar 1 capa con 2 capas):

1. Mientras realiza el experimento, use una CUBIERTA FACIAL para no contaminar accidentalmente las placas. NO toque la gelatina preparada con los dedos (¡esto podría contaminarlos!).
2. Prepare 4 placas de crecimiento de gelatina para probar la eficacia de las cubiertas faciales de tela. Etiquete las placas como # 0: (sin cubierta), # 1: (1 capa), # 2: (2 capas) y # 3: (la cubierta facial que ha estado usando). Primero probará 2 capas de tela.
3. Dobla un trozo de tela por la mitad. Cubra la ventana de la caja con 2 capas de tela; use cinta adhesiva para ayudar a mantenerla en su lugar.
4. Gire la caja de modo que la ventana cubierta de tela quede hacia arriba. Coloque el plato n. ° 2 en la caja, debajo de la ventana cubierta.
5. Desde aproximadamente 5 pulgadas de distancia de la tela, en un ángulo de aproximadamente 45 grados, rocíe DOS VECES en la ventana. Espere 30 segundos para que caigan las microgotas, luego deslice con cuidado la placa fuera de la caja de prueba en la caja de cultivo de gérmenes, ¡asegúrese de no tocar la gelatina!
6. Retire el paño de la caja. Limpie la caja de prueba con un pañuelo y deseche el pañuelo, pero guarde la caja para el resto del experimento.
7. **Repita los pasos 2 a 6** utilizando
   a. 1 capa de tela (placa n. ° 1).
   b. su propia cubierta facial (placa n. ° 3).
   c. sin tapa (placa # 0).
8. Cierre el recipiente de cultivo de gérmenes. Déjelo en un lugar cálido donde pueda permanecer tranquilo pero observado durante unos días (en la parte superior del refrigerador funciona bien).

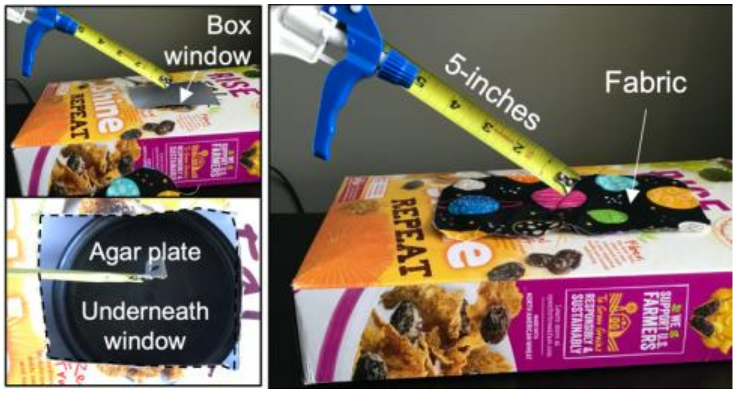

9. Deseche la caja, el paño usado y los pañuelos con cuidado. ¡Limpia la zona de trabajo y lávate bien las manos!

10. Revise las placas de gelatina después de 24-48 horas. Recuerde usar su cubierta facial limpia y lavarse las manos antes de revisar las placas. Cuente el número de manchas (unidades formadoras de colonias o UFC) que se han formado en cada placa. Si es posible, deje las placas en un recipiente durante la observación. ¡NO toque la superficie de las placas! Puede usar el mismo cuadro para el Experimento 4.

Terminar:

1. Registre sus datos en la siguiente tabla.
2. Lávese las manos después de observar las placas.
3. Repita la observación después de 48 horas. Registre el número de unidades formadoras de colonias (UFC).
4. Deseche las placas germinales en la basura, lave bien el área y finalmente vuelva a lavarse las manos.

Comparta sus datos: ingrese los resultados de la Columna G en el formulario o **Table 2** aquí:

Tabla 2. Ingrese a continuación los resultados del Experimento 3.

	A	B	C	D	E	F	G
**Plato**	Colonias / placa a las 0 horas	Colonias / placa a las 24 horas	Colonias / placa a las 48 horas	Diámetro de la placa en cm	Radio de la placa (la mitad de la columna D) en cm	Área de la placa = pi xr^2= (3,14 x radio x radio) en cm^2	**Colonias / cm^2 (columna C dividida por la columna F)**
**0**	0						____ = Línea de base G
**1**	0						
**2**	0						
**3**	0						

Responde las siguientes preguntas

Q3.1 ¿Qué observaste?

Q3.2 ¿Fue eficaz una capa de tela para prevenir la transferencia de gérmenes?

Q3.3 ¿Fueron dos capas de tela igual o más efectivas que una capa?

Q3.4 ¿Pueden una o dos capas de tela contener todos los gérmenes producidos por los estornudos?

Q3.5 ¿Tu cara cubrirse mejor que no cubrirse para prevenir la transferencia de gérmenes?

Comparta sus resultados en linea en Ingles (https://bit.ly/facemaskchallengedata) o Espanol https://forms.gle/iWgE8JFeDvezxXTq6!
Puede tambien compartir sus resultados escaneando el código QR a continuación

Mensaje para llevar a casa #FaceMaskChallenge Experimento 3:

- Una capa de tela debería ayudar a contener los gérmenes, pero no es la mejor.
- ¡Dos capas de tela son incluso mejores!
- Incluso con dos capas de tela, no todas las gotas quedan atrapadas en la cubierta facial, por lo que es muy importante lavarse las manos y practicar una buena higiene.
- Dado que los gérmenes de las microgotas quedan atrapados en la cubierta de la cara, es importante que lave la cubierta de la cara con regularidad.

EXPERIMENTO 4 ¿Cuántos gérmenes salen de mi boca mientras hablo?

Concepto principal: Todos producimos microgotas que contienen gérmenes mientras hablamos.

Grado de dificultad: Moderar.

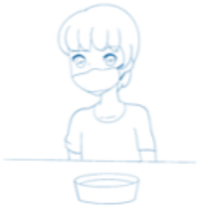

Nuevo descubrimiento: Una cubierta facial evita que nuestras gotas de saliva contaminen el medio ambiente.

Objetivo de aprendizaje: Al hablar con o sin mascarilla y atrapar los gérmenes en las placas de crecimiento de gelatina, el objetivo del experimento es determinar con qué eficacia las barreras de tela evitan la propagación de las gotas orales.

Duración: 48-72 horas.

Materiales (avanzado, preparar1 día antes)

- 2 placas de crecimiento de germen de gelatina (ver Experimento 3).
- 1 caja de cereal vacía para incubar placas de crecimiento de gérmenes.
- Mascarilla de tela o cubierta con 2 capas de textil que cubren tanto la nariz como la boca.
- O pieza de tela preferida.

Procedimiento:

Parte A. El día anterior: prepare placas de germen de gelatina (o compre agar); consulte el Experimento 3, Parte A.

Parte B. El experimento (para determinar cuántas gotas producimos cuando hablamos en voz alta):

1. Descarga gratis Aplicación CDC Noise a un teléfono inteligente, ábralo y ajuste el volumen del habla a 70 decibeles.
2. Etiquete 2 placas de crecimiento de gelatina como "# 4 discurso abierto" y "# 5 discurso cubierto".
3. Prepare un recipiente tapado listo para colocar los platos después de ser rociados. Una caja de cereal sin cortar sería lo mejor. Alternativamente, se puede usar una bolsa grande con cierre de cremallera sobre un recipiente de almacenamiento de plástico limpio. Este será su recipiente de incubación.
4. Siéntese en una mesa y coloque una placa de crecimiento de gelatina con la marca "# 4 openpeech" en la mesa a unas 8 pulgadas de su boca.
5. Tome un sorbo de agua y lea el siguiente pasaje lenta y claramente como si estuviera hablando con alguien al otro lado de la habitación (~ 70 decibeles con el brazo extendido):

"Soy un científico ciudadano. Voy a leer esto en voz alta como un experimento para probar la importancia de las cubiertas faciales. Quiero ver cuántas gotas de saliva produzco que contaminan el medio ambiente mientras cuento del 1 al 100. [… cuente en voz alta hasta 100]. ¡Gracias y mis mejores deseos de buena salud! "

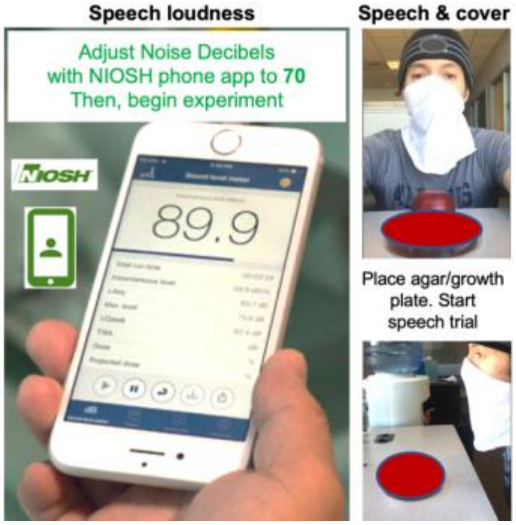

6. Transfiera con cuidado la placa #openspeech al recipiente de incubación.
7. **Cubra su cara y nariz con doble capa de tela.**.
8. Siéntese en una mesa y coloque una placa de crecimiento de gelatina marcada como "discurso cubierto # 5" en la mesa a unas 8 "de su boca.
9. **Repetir** el discurso en el paso 4, arriba y transfiera cuidadosamente la placa de discurso cubierta # 5 al recipiente de incubación.
10. Cierre el recipiente de incubación. Déjelo en un lugar cálido donde pueda permanecer tranquilo pero observado durante aproximadamente 2 días (en la parte superior del refrigerador funciona bien).
11. Revise las placas germinales después de 24 horas. Recuerde usar su cubierta facial limpia y lavarse las manos antes de revisar las placas. Cuente el número de manchas (unidades formadoras de colonias o UFC) que se han formado en cada placa.

EXPERIMENTO 4, continuación

Terminar:

1. Registre sus datos en la siguiente tabla.
2. Lávese las manos después de observar las placas.
3. Repita la observación después de 48 horas. Registre los recuentos de CFU.
4. Deseche las placas germinales y la caja de cultivo en la basura, lave bien el área y finalmente vuelva a lavarse las manos.
5. Comparta sus datos: ingrese los resultados de la Columna G en el formulario aquí: https://bit.ly/facemaskchallengedata

Tabla 3. Ingrese a continuación los resultados del experimento 4

	A	B	C	D	E	F	**GRAMO**
**Plato**	UFC a las 0 horas	UFC a las 24 horas	UFC a las 48 horas	Diámetro de la placa en cm	Radio de la placa (la mitad de la columna D) en cm	Área de la placa = pi xr^2= (3,14 x radio x radio) en cm^2	**UFC / cm^2 (columna C dividida por la columna F)**
**4**	0						____ = Línea de base G
**5**	0						

Responde las siguientes preguntas

Q4.1 ¿Qué observaste? ________________________

Q4.2 ¿Fue eficaz una cubierta facial de dos capas para prevenir la propagación de gérmenes? ________________________

Q4.3 ¿La tela contenía todos los gérmenes? ________________________

Q4.4 ¿Qué otra higiene es necesaria para la propagación eficaz de gérmenes? ________________________

Comparta los resultados en línea aquí: https://bit.ly/facemaskchallengedata o escanee el código QR a continuación

Mensaje para llevar a casa #FaceMaskChallenge Experiment 4:

- Transmitimos muchos gérmenes invisibles simplemente hablando; tenga esto en cuenta cuando practique el distanciamiento social
- Una parte de la solución para el COVID u otras infecciones que se propagan por gotitas es usar una cubierta facial y practicar la higiene y el lavado de manos cuidadosos. ¡Difundir la palabra! Imprima y publique carteles recordatorios:

https://figshare.com/articles/Door_Signs_to_Promote_Public_Droplet_Safety_Amidst_COVID-19/12202808/1

Para profesores: La aplicación del teléfono mostrado en la figura de la página 10 y los detalles científicos del Experimento 4 se describen en https://doi.org/10.3389/fmed.2020.00504

Glosario

Aerosol: una sustancia que puede viajar por el aire sin líquido. Una pequeña porción de un estornudo humano se convierte en aerosol.
Unidad formadora de colonias (CFU):una estimación del número de bacterias viables (reproductoras) en una muestra; un grupo de bacterias reproductoras que es lo suficientemente grande como para verlo a simple vista
Gotita: sustancia transportada en un diminuto volumen de líquido en el que la tensión superficial define la forma. La mayor parte de un estornudo humano son gotas.
Epidemia: ocurrencia generalizada de una enfermedad infecciosa en una comunidad.
Mascarilla o cubierta facial: cualquier trozo de tela que se coloque sobre la boca y la nariz para evitar la propagación de infecciones respiratorias.
Mascarilla médica o quirúrgica: equipo de protección personal que cumpla con estándares de fabricación específicos; reduce la propagación de infecciones entre los trabajadores sanitarios y los pacientes.
Pandemia: enfermedad infecciosa que prevalece en muchas comunidades.
Virus: una partícula tan pequeña como 20 nanómetros que puede entrar e infectar las células. Los virus se multiplican dentro de las células de su anfitrión utilizando el material celular del anfitrión. Miles de nuevas partículas de virus salen de la célula y escapan a nuevos huéspedes a través del fluido celular.

Otros recursos

Módulo de ciencia ciudadana de Face Mask Challenge (sitio web)
https://sites.google.com/kent.edu/face-mask-challenge/face-mask-challenge-home
Enciclopedia Británica: COVID-19 https://www.britannica.com/explore/savingearth/covid-19/
La escala de las cosas: nanoescalas https://www.nano.gov/nanotech-101/what/nano-size
Máscara facial Desafío Ciencia ciudadana Compartir datos
https://docs.google.com/forms/d/e/1FAIpQLSd9cV7HQzxr49MsC-icHCzxIOnlhX2z7e7iza3cJ-NGzJaFRw/viewform
Recordatorios de letreros de puerta imprimibles para fomentar el uso de máscaras faciales
https://figshare.com/articles/Door_Signs_to_Promote_Public_Droplet_Safety_Amidst_COVID-19/12202808/1
Aplicación de medidor de nivel de sonido NIOSH: https://www.cdc.gov/niosh/topics/noise/app.html

---

Authors: Sarah E. Eichler[1], Austin P. Hopperton[2], Juan Jose Alava[3], Antonio Jr. Pereira[4], Rukhsana Ahmed[5], Zisis Kozlakidis[6], Sanja Ilic[7], Alex Rodriguez-Palacios[2,8]

Cartoonist: Maddie Rosemark

Affiliations: [1]Department of Biological Sciences, Kent State University, USA; [2]Division of Gastroenterology and Liver Disease, Case Western Reserve University School of Medicine, USA; [3]Institute for the Oceans and Fisheries, The University of British Columbia, Canada; [4]Institute of Technology, Federal University of Pará, Brazil; [5] Department of Communication, University at Albany, SUNY, USA; [6]International Agency for Research on Cancer, World Health Organization, Lyon, France; [7]Department of Human Sciences Human Nutrition, The Ohio State University, USA; [8]University Hospitals Research and Education Institute, University Hospitals Cleveland Medical Center, Cleveland, OH, USA.

Translations: Juan Jose Alava, Diana M. Rodriguez, Diego A. Pulido, Zisis Kozlakidis, Antonio Jr. Pereira, Alex Rodriguez-P & Maissa Zeghidi.

Open CC BY license http://creativecommons.org/licenses/by/4.0/

Cuatro versions in idiomas adicionales (español, inglés, francés, portugués) forman parte de la presentación inicial y están vinculadas a través de https://sites.google.com/kent.edu/face-mask-challenge/face-mask-challenge-home

Archivo – Versiones Actualizadasd del Module & Contacta los authors si sugerencias / preguntas. Se ha creado un repositorio para el archivo de las últimas versiones del módulo aprobadas por los autores posteriormente a la publicación y para las contribuciones realizadas por la comunidad en: **https://github.com/axr503/education**. Alex Rodriguez-Palacios, Assistant Professor, School of Medicine, axr503@case.edu.

International collaboration of scientists & educators from

SCHOOL OF MEDICINE CASE WESTERN RESERVE UNIVERSITY
University Hospitals
Kent State University
UNIVERSIDADE FEDERAL DO PARÁ
THE OHIO STATE UNIVERSITY COLLEGE OF EDUCATION AND HUMAN ECOLOGY
LMIC BCNet
International Agency for Research on Cancer/WHO
UNIVERSITY AT ALBANY State University of New York
THE UNIVERSITY OF BRITISH COLUMBIA

Module version 1 (August 20/2020)

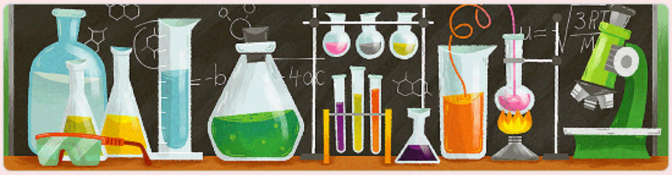

(Espanol) Comparta los resultados de su prueba de cobertura facial: ¡Sea un científico ciudadano y vea crecer su conocimiento!

Comparta anónimamente los resultados de sus experimentos de científicos ciudadanos.

¿Cuál es el propósito de este cuestionario en línea?
Solo habrá 4 preguntas sobre su lugar de residencia y su edad que solo tomarán 1-2 minutos para completar:
No hay riesgos anticipados relacionados con este cuestionario. Todas las respuestas son voluntarias y anónimas. No hay compensación financiera relacionada con su participación.

Dado que algunos experimentos pueden tardar más de un día en completarse, se recomienda anotar todos los resultados en la hoja de instrucciones del experimento y enviar todos los resultados de una vez si planea completarlos todos. Al final de cada envío de experimento, existe la opción de finalizar y enviar sin completar el resto de los experimentos u omitir un experimento e ir a otro.

Al continuar enviando datos, usted acepta compartir información anónima y no identificable. ¿Todavía no está listo? Regrese a las instrucciones del proyecto aquí

https://sites.google.com/kent.edu/face-mask-challenge/face-mask-challenge-home

Next | Page 1 of 8

Información personal

#FaceCoverChallenge

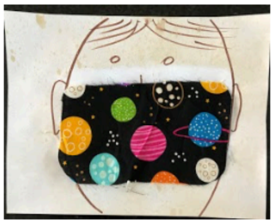

Al hacer clic en la opción << SÍ, acepto >>, indica que ha leído el texto anterior, acepta participar voluntariamente y que tiene 18 años de edad o más. Si no desea participar, haga clic en la opción << NO, no quiero participar en el envío de datos >> o simplemente cierre esta ventana. Si acepta participar, se lo dirigirá automáticamente al formulario de envío de datos. *

- ○ Sí, acepto participar y compartir mis resultados. (Haga clic en SEGUIR para continuar)
- ○ No, no quiero participar (haga clic en SEGUIR para continuar)

¿Es este su primer envío de datos a Ciencia Ciudadana: desafío de prueba de máscara facial? *

Choose ▾

Back | Next | Page 2 of 8

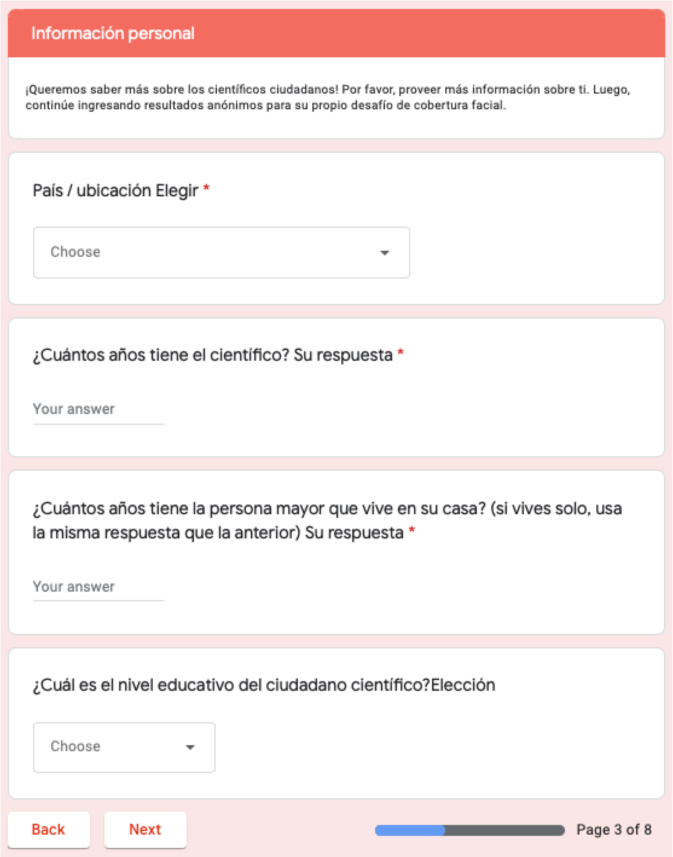

Información personal

¡Queremos saber más sobre los científicos ciudadanos! Por favor, proveer más información sobre ti. Luego, continúe ingresando resultados anónimos para su propio desafío de cobertura facial.

País / ubicación Elegir *

Choose

¿Cuántos años tiene el científico? Su respuesta *

Your answer

¿Cuántos años tiene la persona mayor que vive en su casa? (si vives solo, usa la misma respuesta que la anterior) Su respuesta *

Your answer

¿Cuál es el nivel educativo del ciudadano científico?Elección

Choose

Back Next Page 3 of 8

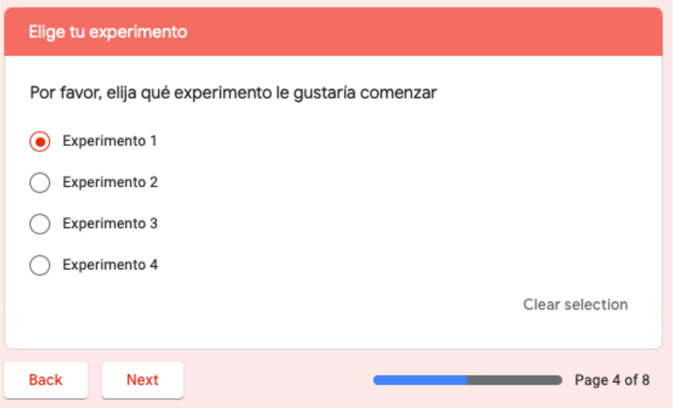

Elige tu experimento

Por favor, elija qué experimento le gustaría comenzar

- Experimento 1
- Experimento 2
- Experimento 3
- Experimento 4

Clear selection

Back Next Page 4 of 8

Experimento 1: ¿Qué tan lejos viajan las gotas?

Exp.1-P1 / 7. SIN PROTECCIÓN TEXTIL: Cuando el aspersor (botella de rociar/spray) estaba en posición plana, ¿cuál es el número de papel más distante en el que detectó las gotas? (0 = sin gotas)

	0	1	2	3	4	5	6	7	
más cercano al aspersor (botella de rociar/spray)	○	○	○	○	○	○	○	○	más alejado del aspersor (botella de rociar/spray)

Exp.1-P2 / 7. - SIN PROTECCIÓN TEXTIL: Cuando la botella de rociar (spray) estaba en posición plana, ¿cómo describiría la cantidad de gotas que contaminan el papel número 3?

- ○ Bajo (0-10)
- ○ Promedio (11-100)
- ○ Alto (100-500)
- ○ Muy alto (500)

Exp.1-P3 / 7. - SIN PROTECCIÓN TEXTIL: Cuando el aspersor (botella de rociar/spray) estaba inclinado a un ángulo de 10 grados hacia arriba, ¿cuál era el número de papel más distante en el que detectó gotas? (0- Sin gotas)

	0	1	2	3	4	5	6	7	
más cercano al aspersor (botella de rociar/spray)	○	○	○	○	○	○	○	○	más alejado del aspersor (botella de rociar/spray)

Exp.1-P4 / 7. - SIN PROTECCIÓN TEXTIL: Cuando el aspersor (botella de rociar/spray) estaba en un ángulo de 10 grados hacia arriba, ¿cómo describiría la cantidad de gotas que contaminan el papel número 3?

- ○ Bajo (0-10)
- ○ Promedio (11-100)
- ○ Alto (100-500)
- ○ Muy alto (500)

Exp.1-P5 / 7. - SIN PROTECCIÓN TEXTIL: Cuando el aspersor (botella de rociar/spray)spray estaba inclinado en un ángulo de 10 grados hacia abajo, ¿cuál era el número de papel más distante en el que detectó gotas? (0- Sin gotas)

	0	1	2	3	4	5	6	7	
más cercano al aspersor (botella de rociar/spray)	◯	◯	◯	◯	◯	◯	◯	◯	más alejado de la botella de rociar/spray

Exp.1-P6 / 7. - SIN PROTECCIÓN TEXTIL: Cuando el rociado estaba en un ángulo de 10 grados hacia abajo, ¿cómo describiría la cantidad de gotas que contaminan el papel número 3?

- ◯ Bajo (0-10)
- ◯ Promedio (11-100)
- ◯ Alto (100-500)
- ◯ Muy alto (500)

Exp.1-P7 / 7 - ¿Qué posición roció más lejos?

- ◯ Botella plana
- ◯ Botella inclinada 10 grados hacia arriba
- ◯ Botella inclinada 10 grados hacia abajo
- ◯ Otro

¿Le gustaría realizar otro experimento o finalizar y enviar sus datos?

- ◯ Experimento 2
- ◯ Experimento 3
- ◯ Experimento 4
- ◯ Finalizar y enviar datos

Back Next

Page 5 of 8

Experimento 2: ¿Qué tan efectiva es una barrera protectora de tejido contra las gotas?

Exp.2-P1 /3. ¿Qué tipo de tela / barrera textil usaste? (Puede elegir varias opciones).

- [ ] Algodón
- [ ] Poliéster
- [ ] Algodón-poliéster
- [ ] Seda
- [ ] Allí
- [ ] Toalla
- [ ] Licra
- [ ] camiseta
- [ ] Bufanda
- [ ] Otro

Exp.2-P2 / 3. - CON BARRERA TEXTIL: ¿cuál fue el número de papel más alejado de la botella de rociar/spray (aspersor) en la que detectó las gotas? (0- Sin gotas)

	0	1	2	3	4	5	6	7	
más cercano a la botella	○	○	○	○	○	○	○	○	más alejado de la botella

Exp.2-P3 / 3. - CON BARRERA TEXTIL: ¿Cómo describirías la cantidad de gotas que contaminan el papel número 3?

- ○ Bajo (0-10)
- ○ Promedio (11-100)
- ○ Alto (100-500)
- ○ Muy alto (500)

¿Le gustaría realizar otro experimento o finalizar y enviar sus datos?

- ○ Experimento 1
- ◉ Experimento 3
- ○ Experimento 4
- ○ Terminar y enviar

Clear selection

Back Next Page 6 of 8

Experimento 3: ¿cuántas gotas transportan gérmenes que pueden atravesar dos capas de tela o cubierta textil tejido?

Exp.3-P1 / 3.- Placa/Plato # 0 Sin tela/cubierta textil (columna G: colonias/cm2) Su respuesta

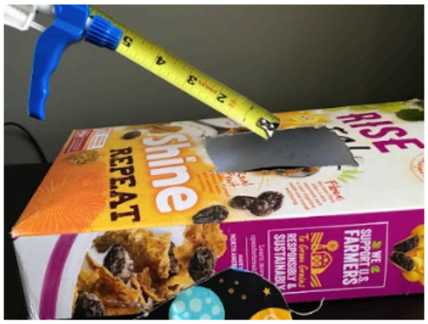

Your answer

Exp.3-P2 / 3.- Placa/Plato # 1 con una capa de tela/cubierta textil (columna G: colonias / cm2)Su respuesta

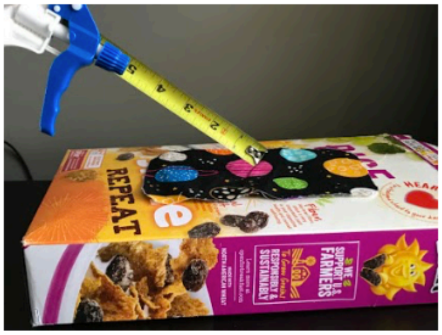

Your answer

Exp.3-P3 / 3.- Placa/Playo # 2 con dos capas de tela/cubierta textil (columna G: colonias / cm2) Su respuesta

Your answer

¿Le gustaría realizar otro experimento o finalizar y enviar sus datos?

- ○ Experimento 1
- ○ Experimento 2
- ◉ Experimento 4
- ○ Finalizar y enviar datos

Clear selection

Back | Next | Page 7 of 8

Experimento 4: ¿Cuántos gérmenes salen de mi boca cuando hablo?

Exp.4-P1 / 2.- SIN COBERTURA FACIAL: placa/plato de voz descubierta/ hablar no cubierto (columna G: colonias / cm2)

Your answer

Exp.4-Q2 / 2.- CON COBERTURA FACIAL: placa/palto de voz cubierto/ hablar cubierto (columna G: colonias / cm2)

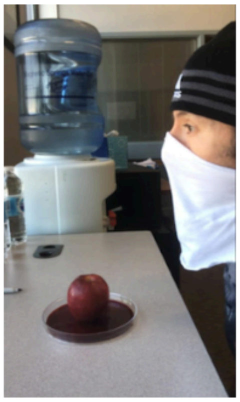

Your answer

¿Le gustaría realizar otro experimento o finalizar y enviar sus datos?

- ○ Experimento 1
- ○ Experimento 2
- ○ Experimento 3
- ◉ Submit and Finish

Clear selection

Back Next Page 8 of 8

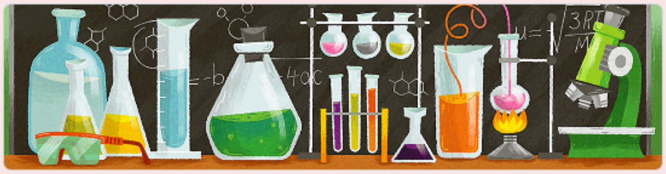

(Espanol) Comparta los resultados de su prueba de cobertura facial: ¡Sea un científico ciudadano y vea crecer su conocimiento!

Click submit to finish.

Back Submit Page 8 of 8

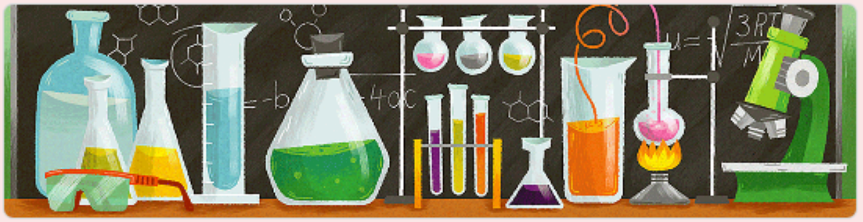

(Espanol) Comparta los resultados de su prueba de cobertura facial: ¡Sea un científico ciudadano y vea crecer su conocimiento!

Thank you citizen scientist! your response has been recorded!

See previous responses
Edit your response
Submit another response

COLLECTIVE RESULTS

(below is a mock series or random responses for illustration)
Please only enter real results so we all could see the real findings.
If some results do not resemble your data, discuss with a teacher.
And ask/read the real laboratory experiment results that were obtained in a Medical Research Center.

Original studies are published here:

https://doi.org/10.3389/fmed.2020.00260

Textile Masks and Surface Covers—A Spray Simulation Method and a "Universal Droplet Reduction Model" Against Respiratory Pandemics. Rodriguez-Palacios A, Cominelli F, Basson AR, Pizarro TT, and Ilic S. (2020) Front. Med. 7:260. doi: 10.3389/fmed.2020.00260

https://doi.org/10.3389/fmed.2020.00504

Germ-Free Mice Under Two-Layer Textiles are Fully Protected From Bacteria in Sprayed Microdorplets: A Functional in-vivo Test Strategy of Facemasks and Filtration Materials. Rodriguez-Palacios A, Conger M, and Cominelli F. (2020) Front. Med. 7:504. doi: 10.3389/fmed.2020.00504

A Citizen Science Facemask Experiment and Educational Modules to Increase Coronavirus Safety in Communities and Schools. Eichler SE, Hopperton AP, Alava JJ, Pereira A, Ahmed R, Kozlakidis Z, Ilic S and Rodriguez-Palacios A (2020) Front. Med. 7:486. doi: 10.3389/fmed.2020.00486

https://doi.org/10.3389/fmed.2020.00486

(Espanol) Comparta los resultados de su prueba de cobertura facial: ¡Sea un científico ciudadano y vea crecer su conocimiento!

1 response

Información personal

Al hacer clic en la opción << SÍ, acepto >>, indica que ha leído el texto anterior, acepta participar voluntariamente y que tiene 18 años de edad o más. Si no desea participar, haga clic en la opción << NO, no quiero participar en el envío de datos >> o simplemente cierre esta ventana. Si acepta participar, se lo dirigirá automáticamente al formulario de envío de datos.

1 response

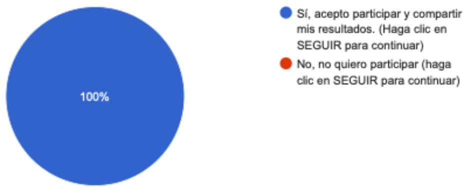

¿Es este su primer envío de datos a Ciencia Ciudadana: desafío de prueba de máscara facial?

1 response

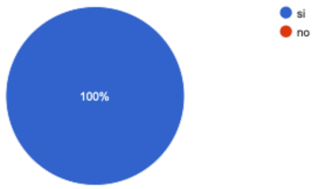

Share your Face Cover Test results - become a Citizen Scientist and watch our knowledge grow!

13 responses

Participant information

Clicking the 'YES, I Agree' option indicates that you have read the above information, voluntarily agree to participate, and that you are 18 years of age or older. If you do not wish to participate, please click, "NO, I do not wish to participate in the data submission", or simply close this window. If you agree to participate you will be automatically directed to the data submission form.

12 responses

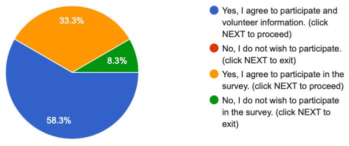

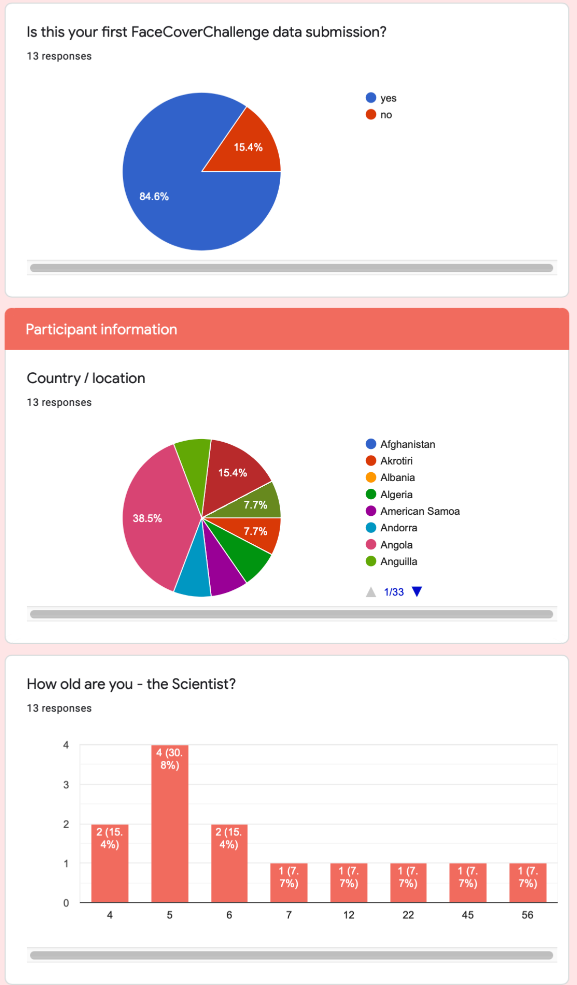

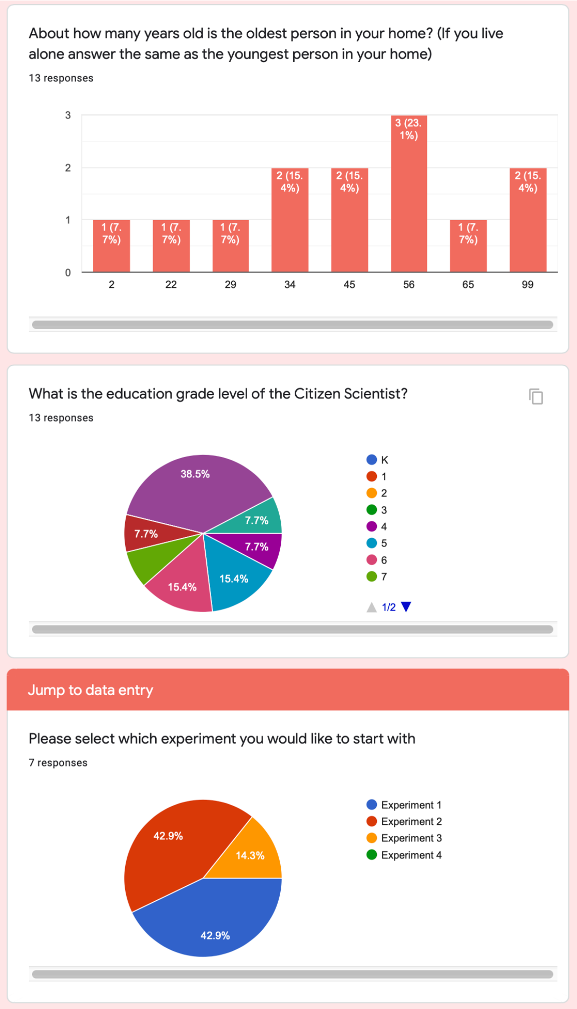

See remaining results in response form link
https://forms.gle/5q7V44nbT8fu6Jvz6

A Citizen Science Facemask Experiment and Educational Modules to Increase Coronavirus Safety in Communities and Schools. Eichler SE, Hopperton AP, Alava JJ, Pereira A, Ahmed R, Kozlakidis Z, Ilic S and Rodriguez-Palacios A (2020) Front. Med. 7:486. doi: 10.3389/fmed.2020.00486
SUPPLEMENTARY MODULE - In English.
Provided by Alex Rodriguez-P. axr503@case.edu.
V1 (August 20, 2020) with the accepted publication.
Results submission form in English:
https://docs.google.com/forms/d/e/1FAIpQLSd9cV7HQzxr49MsC-icHCzxIOnlhX2z7e7iza3cJ-NGzJaFRw/viewform

Comparte tus resultados en linea en:
Ingles: https://bit.ly/facemaskchallengedata, ou en
Frances: https://forms.gle/5q7V44nbT8fu6Jvz6
Espanol: https://forms.gle/iWgE8JFeDvezxXTq6
Portugues: https://forms.gle/EJHauCWTdDbny66N8

frontiers in Medicine

OPINION
published: xx July 2020
doi: 10.3389/fmed.2020.00486

Check for updates

A Citizen Science Facemask Experiment and Educational Modules to Improve Coronavirus Safety in Communities and Schools

Sarah E. Eichler[1], Austin P. Hopperton[2], Juan José Alava[3], Antonio Pereira Jr.[4], Rukhsana Ahmed[5], Zisis Kozlakidis[6], Sanja Ilic[7] and Alexander Rodriguez-Palacios[2]*

[1] Department of Biological Sciences, Kent State University at Salem, Salem, MA, United States, [2] Division of Gastroenterology and Liver Disease, School of Medicine, Case Western Reserve University, Cleveland, OH, United States, [3] Institute for Oceans and Fisheries, University of British Columbia, Vancouver, BC, Canada, [4] Institute of Technology, Federal University of Pará, Belém, Brazil, [5] Department of Communication, University at Albany, SUNY, Albany, NY, United States, [6] International Agency for Research on Cancer, World Health Organization (France), Lyon, France, [7] Department of Human Sciences, The Ohio State University, Columbus, OH, United States

Keywords: COVID-19, mass media, health communication, prevention, intervention, social behavioral changes, facemask, school education

OPEN ACCESS

SUPPLEMENTARY MATERIALS

Education Module #1 ENGLISH
Education Module #2 SPANISH
Education Module #3 FRENCH
Education Module #4 PORTUGUESE

Education Module

FRENCH

Science Citoyenne: Contrôler les Masques et Tissus de Protection

Campagne Pédagogique sur le Coronavirus afin de Promouvoir les Règles d'Hygiènes Respiratoires dans les Écoles

COVID- est une abréviation signifiant Coronavirus Infectious Disease – 2019 (Maladie Infectieuse du Coronavirus -2019) causé par le virus SARS-Cov2. **COVID-19 est une maladie virale qui se transmet principalement par des gouttelettes respiratoires expulsés par le nez et la bouche par la toux et la parole.**

Le COVID-19 atteint plus fortement les personnes âgées et ceux qui sont déjà malades, les symptômes étant essoufflement, une toux sèche, fièvre, des douleurs musculaires et une inflammation systémique qui peut mener à l'hospitalisation. Les enfants sont touchés mais les symptômes restent moins sévères, ils restent toutefois capables de transmettre la maladie.

COVID se transmet de personne à personne par la salive et des microgouttelettes nasales. C'est pourquoi, les représentants gouvernementaux recommandent la distance de deux mètres entre les individus, limiter l'exposition potentielle au virus à des groupes, se laver les mains régulièrement et porter des masques. Puisque les masques sont rares dans certaines régions, des alternatives sont utilisés telles que des écharpes, des mouchoirs ou des masques en tissus. **Le but de ces masques est de limiter la transmission de la salive et les gouttelettes nasales que nous produisons naturellement et propageons lorsque nous parlons, éternuons, toussons.**

Ces activités ont été préparés avec l'intention d'aider les communautés à tester l'utilité des différentes manières de se couvrir le visage. A travers **quatre courtes expériences** vous pouvez, chez vous, quantifier et observer comment les masques peuvent réduire la transmission de fluides et peuvent protéger vos proches.

Ce module est basé sur une recherche dirigée par une centre médical universitaire. Pour plus d'informations:

https://doi.org/10.3389/fmed.2020.00260

Textile Masks and Surface Covers—A Spray Simulation Method and a "Universal Droplet Reduction Model" Against Respiratory Pandemics. Rodriguez-Palacios A, Cominelli F, Basson AR, Pizarro TT, and Ilic S. (2020) Front. Med. 7:260. doi: 10.3389/fmed.2020.00260

https://doi.org/10.3389/fmed.2020.00504

Germ-Free Mice Under Two-Layer Textiles are Fully Protected From Bacteria in Sprayed Microdorplets: A Functional in-vivo Test Strategy of Facemasks and Filtration Materials. Rodriguez-Palacios A, Conger M, and Cominelli F. (2020) Front. Med. 7:504. doi: 10.3389/fmed.2020.00504

En utilisant ce module de collaboration international veuillez mentionner si possible, ce module et cet **Article**:

A Citizen Science Facemask Experiment and Educational Modules to Increase Coronavirus Safety in Communities and Schools. Eichler SE, Hopperton AP, Alava JJ, Pereira A, Ahmed R, Kozlakidis Z, Ilic S and Rodriguez-Palacios A (2020) Front. Med. 7:486. doi: 10.3389/fmed.2020.00486

https://doi.org/10.3389/fmed.2020.00486

Ce module éducatif propose une introduction au virus COVID -19 et met en valeur l'intérêt de masques pour la prévention de la transmission de germes respiratoire.

A travers, une série de quatre expériences fait maison, vous serez capable de quantifier à quel point les masques et alternatives peuvent aider à contrôler les maladies respiratoires transmise par voies aériennes.

Expérience n°1- Jusqu'où des gouttelettes but voyager?

Expérience n°2- Quel est l'efficacité d'une barrière protectrice en tissus contre les gouttelettes?

Expérience n°3- Quelle quantité de gouttelettes transportant des germes peuvent passer à travers deux-couches de tissus?

Expérience n°4- Quelle quantité de bactérie je transmets lorsque je parle?

A la fin des activités, les participants peuvent partager les résultats de leur expérience via un questionnaire internet et vous pouvez comparer vos résultats avec d'autres citoyens en anglais (page 2) ou en français!: https://forms.gle/5q7V44nbT8fu6Jvz6

Les données partagées sont toutes anonymes: Aucune données personnelle seront collectés.

Ce module est aussi disponible dans d'autres languages (e.g., **Espagnol**, **Anglais**, **Portuguais**) **Article scientifique** et sur:https://bit.ly/facemaskchallenge (website) & **https://github.com/axr503/education**

Si vous êtes enseignant et souhaitez nous faire savoir que vous implémentez le module dans votre école / classe: https://forms.gle/Sg36k3HceMos1Xpb8
Contact: Alex Rodriguez-P. axr503@case.edu

International collaboration of scientists & educators from

School of Medicine Case Western Reserve University; University Hospitals; Kent State University; The Ohio State University College of Education and Human Ecology; LMIC BCNet; International Agency for Research on Cancer/WHO; University at Albany State University of New York; Universidade Federal do Pará; The University of British Columbia

INTRODUCTION AUX ACTIVITES SCIENTIFIQUES ET CITOYENNES

En tant que scientifique, des simulations seront faites en créant des nuages de gouttelettes en utilisant des liquides ménagers. Le projet consiste en quatre expériences très simples qui nécessite seulement un flacon vaporisateur et des éléments présents à la maison Partager les données que vous collectez et vous allez observer comment les masques peuvent protéger votre famille, amis et limiter la transmission de germes.

Les quatre expériences sont décrites ci-dessous. Elles peuvent être complété en utilisant des ingrédients de base, des ustensiles de cuisine et quelques objets recyclés qui seront listés dans la page suivante. Ces activités auront pour objectif de mesurer la quantité de gouttelettes peuvent passer à travers le matériel d'un masque et jusqu'où elles peuvent traverser par un éternument simulés.

Les scientifiques Citoyen vont pouvoir apprendre directement comment les gouttelettes nasales peuvent causer une contamination, comment les masques en tissus fonctionnent et apprendre leur importance de son utilisation durant une pandémie. Si vous le souhaitez les données scientifiques que vous récolter peuvent être partagé et vous feriez part d'un projet scientifique mondial pour mieux comprendre les maladies et comment les prévenir tel un véritable scientifique. **Partager vos résultats ici en anglais: https://bit.ly/facemaskchallengedata, ou en français: https://forms.gle/5g7V44nbT8fu6Jvz6**

Public Cible: Recommandé pour les Enseignants & Parents d'élèves du CE2 et supérieur. Accessible pour tout âges avec supervision si nécessaire.

Matériel de base

- 2 verres d'un liquide coloré (boisson énergétique pour sportif, jus de fruit tel que le raisin, café ou boisson gazeuse).
- 1 flacon vaporisateur.
- 28 feuilles de papier de format A4.
- 1 Ruban à mesurer ou une règle.
- 1 boîte de céréale vides.
- Tissus de format 25cm x 25cm qui peut être salit tel qu'une couverture, une taie d'oreiller, un t-shirt, écharpe, bandana, nappe en tissus. Etc.
- Ruban adhésif.
- Papier essuie-tout.
- En option: papier quadrille (imprimé ou dessiné par vous-même).

Matériels pour l'incubation
(à préparer à l'avance)

- 1 boîte de céréale vides.
- 6 à 10 petits récipients (tel que les ramequins).
- 1 càc de sucre.
- 1 cube de bouillon de bœuf ou similaire.
- 2 sachets de gélatine; 7 grammes chacun.
- 1 verre d'eau.
- Micro-onde et récipient en verre ou une casserole avec couvercle.
- Des sacs transparents avec une fermeture à glissières
- un masque.

https://www.instructables.com/id/Homemade-Nutrient-Agar/

Regardez cette vidéo de la Nature : **The Physics of the Sneeze**, (Mai 2016, YouTube) pour vous aider à comprendre la dynamique des fluides.(en anglais)

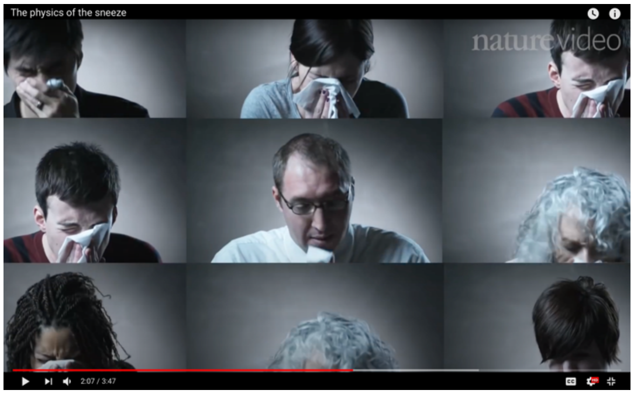

En preparation des expériences, vous pouvez regarder une vidéo d'un ralenti d'un éternuement (minute 2:00): https://youtu.be/bFxgVksID-k?t=107

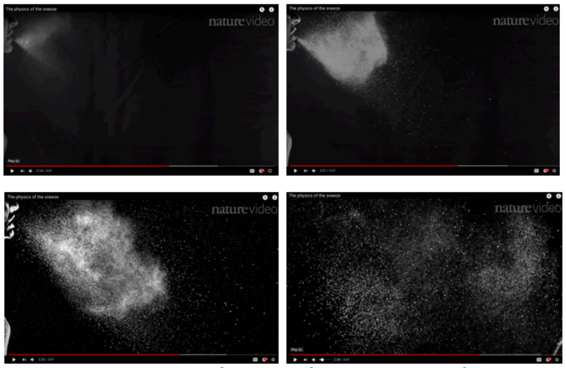

Vous pouvez essayer de reproduire, ce type de nuage de gouttelettes en changeant les réglages du flacon vaporisateur

1ère Expérience
Jusqu'où des gouttelettes peut-elles voyager ?

Notion fondamentale: Dans un nuage de gouttelettes il y a différentes tailles de gouttelettes : Macro (de grande taille et facile à voir) et Micro (plus petite et difficile à voir).

Difficulté: Facile.

Découverte : Les gouttes les plus larges voyagent plus loin que les gouttelettes plus petites.

Objectif du module : Observer à quelle distance les gouttelettes d'un spray peuvent voyager et donc se transmettre.

Durée: 30 minutes.

Matériels

- Assez de liquide alimentaire coloré pour remplir un flacon vaporiseur à moitié.
- 28 feuilles de papier blanche de format A4.
- 1 flacon vaporisateur.
- Beaucoup d'espace.
- Un stylo.
- 1 boîte de céréales vide.
- Des ciseaux.
- 7-8 couverts pour être utilisé en tant que poids.

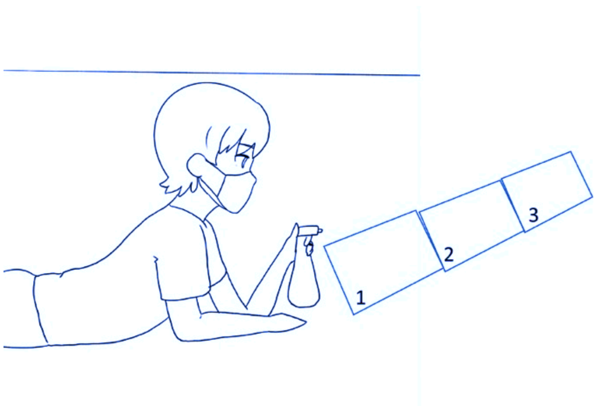

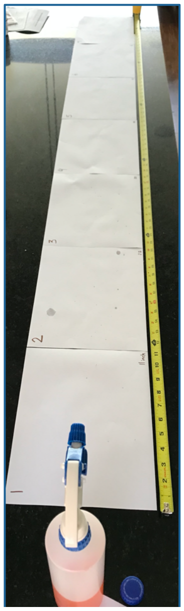

Protocole:

Partie A – Pulvérisez le contenu du flacon <u>à plat</u> sur le sol:

1. Posez 7 feuilles de papier sur le sol côte à côte en les numérotant de 1 à 7. (1 étant le plus proche et 7 le plus éloignée).
2. Remplissez le flacon avec le liquide de votre choix, Réglez le vaporisateur pour qu'il donne un jet similaire à de la brume (voir vidéo présentée plus tôt page …). Conservez les réglages tout au long de l'expérience 1.
3. A 15 cm du papier numéro 1, Placez-vous à plat ventre, déposez le flacon au sol pour qu'il soit stable et en visant le centre de la feuille.
4. Faites une vaporisation complète et attendez 30 seconds pour que les gouttes terminent leur voyage. Observez et notez ce que vous observez dans le tableau 1 ci-dessous.

1ère Expérience, suite

Partie B - Pulvérisez le contenu du flacon <u>avec différents angles</u> :

1. Remplacez les feuilles de papier et disposez les comme dans la première Partie.
2. En utilisant le même flacon, inclinez le vers le haut à environ 10° degré en utilisant un stylo qui sera placé sous le flacon.
3. Faites une vaporisation complète et relevez les résultats.
4. Faites de même pour l'incliner vers le bas en utilisant le même stylo. Relevez les résultats dans le Tableau 1

Répondez au question suivantes dans le Tableau 1 ci-dessous, pour chaque spray de l'expérience.

Q1.1 Comment évalueriez-vous la quantité de gouttelettes qui ont touchée et donc contaminée le troisième papier ?
Q1.2 Utilisez cette échelle pour qualifier approximativement le nombre de gouttelettes présente sur le papier numéro 3?
Q1.3 Avec quelle position le flacon projette les gouttelettes le plus loin ? A quelle mesure ?__________
Q1.4 Les gouttelettes les plus éloignés sont-elles des macro ou micro gouttelettes (Selon le schéma ci-dessous)

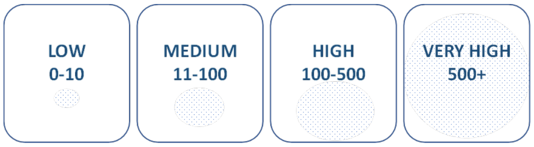

Tableau 1. Ecrivez les résultats récoltés de l'Expérience n°1.

Position du vaporisateur	***Numéro du papier le plus éloignés touchés par les gouttelettes (1-7)?***	***Evaluation de la contamination de gouttelettes sur le troisième papier. (Faible, Moyen, Fort ou Très Fort ?)***
Bouteille **à plat**		
Dirigée vers le **haut**		
Dirigée vers le **bas**		

Conclusion #FaceMaskChallenge:

- Les gouttelettes provenant de notre bouche ou nez, voyage très loin de nous s!
- Si on éternue avec notre visage dirigé vers le haut, cela va encore plus loin!
- **Regardez vers le bas quand vous éternuez ou toussez!**

Partager vos résultats en ligne: https://forms.gle/5q7V44nbT8fu6Jvz6 ou scanner le code QR ci-dessus.

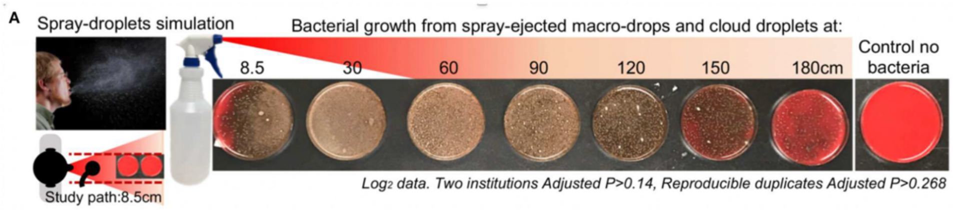

Pour les professeurs: Ce schéma ci-dessus et les détails scientifiques de **l'expérience 1 à 3** sont décrits dans cet article
https://doi.org/10.3389/fmed.2020.00260

2ème Expérience: Quel est l’efficacité d’une barrière protectrice en tissus contre les gouttelettes ?

Notion fondamentale: Le tissue peut stopper la transmission de gouttelettes liquides.

Difficulté: Facile.

Découverte: Une épaisseur de tissus utilisée comme barrière peut réduire la distance des gouttelettes par 90%.

Objectif du module: Le but de l’expérience est de déterminer quelle quantité de gouttelettes peuvent passer à travers un tissus.

Durée: 30 minutes.

Matériels

- 1 flacon vaporisateur.
- Liquide coloré (boisson énergétique, jus de fruit, café ou boison gazeuse).
- 14 feuilles de papier de format A4.
- Beaucoup d’espace sur une table.
- Boite de céréales vides.
- Tissus de format 25cm x 25cm.
- Ciseaux.
- 7-8 couverts pour être utilisé en tant que poids.

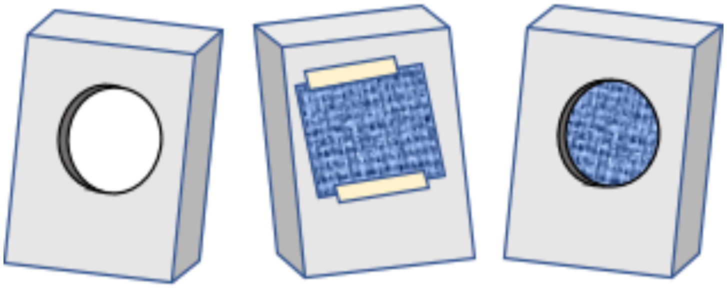

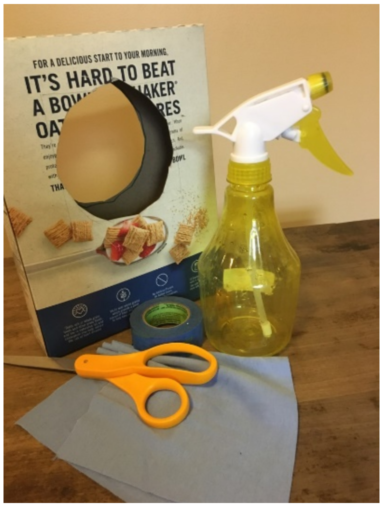

Procedure:

1. Découpez une fenêtre de 10cm de diamètre de chaque côté de la boîte en carton, comme illustré dans les images ci-après. A hauteur du spray quand le flacon est placé à plat sur une surface. Si ce n’est pas possible, couper les fenêtres le plus haut possible.
2. A l’aide d’un adhésif, scotchez une épaisseur de tissus sur l’une des fenêtre.
3. Déposez la boîte en position vertical en déposant des objets lourds tel que des couverts au fond de celle-ci.
4. Placez sept feuilles de papier sur la surface, côte à côte en les numérotant de 1 à 7 (1 étant le plus proche)
5. Placez la boîte directement en face de la feuilles numéro 1 sans laisser d’espaces. Faites en sorte que le flacon est à plat en face des fenêtres découpés ou faire en sorte que le spray soit le plus horizontal possible.

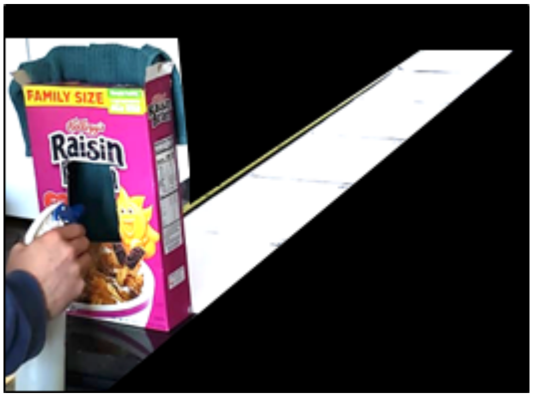

6. Faites une vaporisation complète. Attendez 30 secondes pour laisser les gouttelettes voyager. Observez les dépôts sur les feuilles et répondez aux questions ci-dessous. Conservez la boîte pour **l’expérience 3.**

Répondez aux questions suivantes.

Q2.1 Quel est le papier le plus éloigné ayant été touché par les gouttelettes?__________

Q2.2 En utilisant l'échelle de la 1er expérience comment qualifieriez-vous la quantité de gouttelettes sur la page 3?

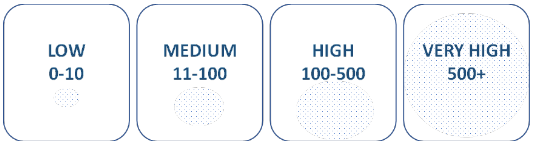

Partager vos résultats en ligne: https://forms.gle/5q7V44nbT8fu6Jvz6

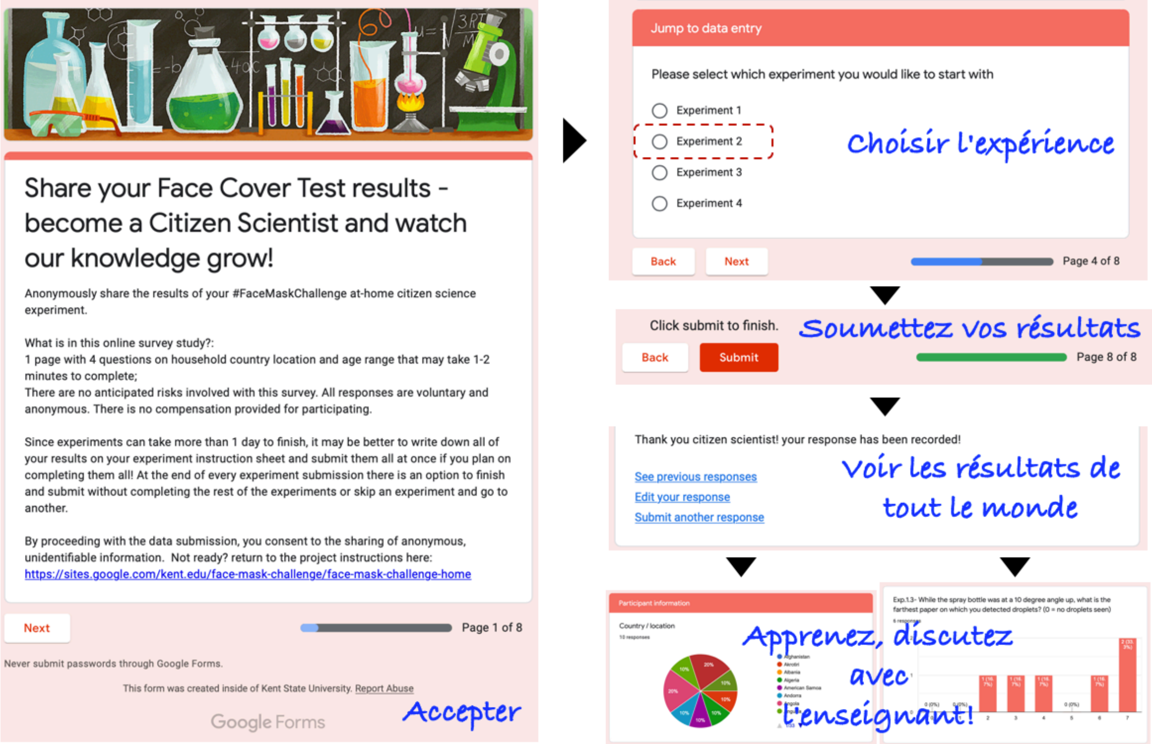

Ou scannez le code QR ci-dessous

Conclusion #FaceMaskChallenge:

- La transmission de gouttelettes sont réduites par une protection telle que du tissus.
- Les gouttelettes sont connus en tant que transporteurs de germes.
- Toutefois une partie d'entre elles passent à travers et atterrissent sur les surfaces donc **Ils faut donc s'assurer de bien se lavez les mains, de vous couvrir le visage avec un masque réutilisable et éviter de toucher des objets pour ne pas transmettre la maladie!**

3ème Expérience: Quelle quantité de gouttelettes transportant des germes peuvent passer à travers deux-couches de tissus?

Notion fondamentales: Les gouttelettes émanant de la bouche et le nez peuvent transporter des bactéries ou virus très loin du point d'origine.

Difficulté: Expert.

Découverte: Un masque réduit le nombre de gouttelettes transportant des virus et bactéries à 98% .

Objectif: En utilisant un flacon vaporisateur rempli de bactérie (yaourt dilué, de la terre ou des résidu d'une éponge) et en utilisant des gélatines permettant l'incubation des microorganisme, l'objectif est de déterminer la quantité de gouttelettes transportant ces germes peuvent traverser les masques réutilisables

Durée: 48-72 heures

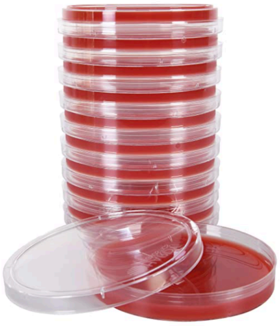

Matériels pour l'incubation
(à préparer à l'avance.)

- 6 à 10 petits récipients (tel que les ramequins).
- Une boîte de céréales vide.

Ingrédients pour le gel :
- 1 càc de sucre.
- 1 cube de bouillon de bœuf.
- 2 sachets de gélatine (7 grammes).
- 1 verre d'eau.
- Micro-onde et récipient en verre ou une casserole avec couvercle.
- Des sacs transparents avec une fermeture à glissières.

https://www.instructables.com/id/Homemade-Nutrient-Agar/

Vous pouvez aussi acheter prêt pour utilisation gel d'incubation (Amazon, Columbia Blood Agar, 5% sheep blood, or tryptic soy agar)

Protocole:

Partie A. Préparez la gélatine pour la pousse des germes:

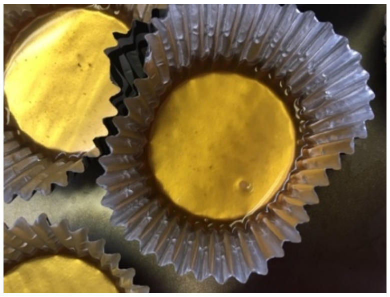

1. Mélanger dans un récipient 1 verre d'eau, une cuillère à café de sucre and un cube de bouillon et passer le mélange au microonde pendant 2 minutes.
2. Mélanger à nouveau et réchauffer le tout pendant 2 minutes. Lorsque cela fait, laisser la boîte refroidir pendant 5 minutes.
3. Vous pouvez faire la même chose avec un bain-marie. N'oubliez pas de vous laver les mains et de porter un masque pour éviter les contamination.
4. Ajouter doucement les deux paquets de gélatine en poudre tout en mélangeant. Elle permettera d'obtenir au moins 6 différents gels.
5. Soigneusement verser le mélange dans les différents récipients avec une épaisseur d'un cm minimum. Déposer immédiatement les gels dans un sac transparents laissez ouverts.
6. Placez-les dans une zone froide (un four fermé) pour laisser le gel se solidifier. Préparer au moins 4 gel pour la partie D. Si vous souhaitez testez différents types de tissus préparer 2 gel supplémentaire par tissus. 2 gel sont nécessaires pour l'expérience 4 qui à conserver dans un sac transparents avec une fermeture à glissière.

3ème Expérience, suite

Partie B. Préparer le bouillon de germes:

1. Dans un verre d'eau tiède, ajouter une cuillère à café de yaourt, terre et mélanger jusqu'à sa dissolution.
2. Déverser cette solution dans un flacon vaporisateur propre libellé Germes. Cette solution contient des microbes inoffensifs qui seront visibles après leur pousse sur le gel.

Quelle source de germes avez-vous utilisé pour votre solution ? ______________________

Partie C. Préparez les boites de test:

1. Utilisez la boîte de céréales de l'expérience 2 après avoir retiré le tissus utilisée précédemment. Celle-ci portera les tissus que vous souhaitez tester .
2. Préparez aussi une seconde boîte (non découper) qui contiendra les gels juste après avoir reçu une pulvérisation. Une alternative possible sera des sacs plastique.

Partie D. L'Expérience:

1. Le long de l'expérience portez un masque pour éviter que les gel utilisés pour éviter de les contaminé. Faites attention de ne toucher la surface avec vos doigts.
2. Préparez les quatre gels pour tester l'efficacité des masques en tissus. Légendez les gels #0 (sans tissus), #1 (une épaisseur de tissus), #2 (deux épaisseurs de tissus), #3 (le masque en tissus que vous utilisez). On testera les deux épaisseurs de tissus en premier (gel #2).
3. Pliez un tissu en deux (il ne faut pas qu'il soit utilisé précédemment) Couvrez la fenêtre avec ce tissus. En utilsant de l'adhésif.
4. Déposez le gel #2 sur une surface plate et déposer la boîte avec les fenêtre au-dessus de celui-ci à plat.
5. A 12 cm du tissus à 45 degrés, faite deux vaporisations complète par la fenêtre. Attendez 30 secondes pour les gouttelettes atterrissent sur le gel.
6. Doucement levez la boîte et ranger le gel dans la chambre d'incubation. Retirez le tissus de la boîte et nettoyez la boîte soigneusement. Et conservez la boite pour la suite de l'expérience.
7. **Répétez les étapes de 2 à 6 en utilisant**
8. Une couche de tissus (gel #1)
9. Votre masque réutilisable (gel #3)
10. ucune protection (gel #0)
11. Fermez la boite et laissez-le dans un endroit chaud où il peut rester quelques jours sans être bouger (le dessus d'un frigo par exemple).
12. Débarrasser vous de la boite, des tissus utilisé en faisant attention. Nettoyer la zone de travail soigneusement et lavez-vous bien les mains!

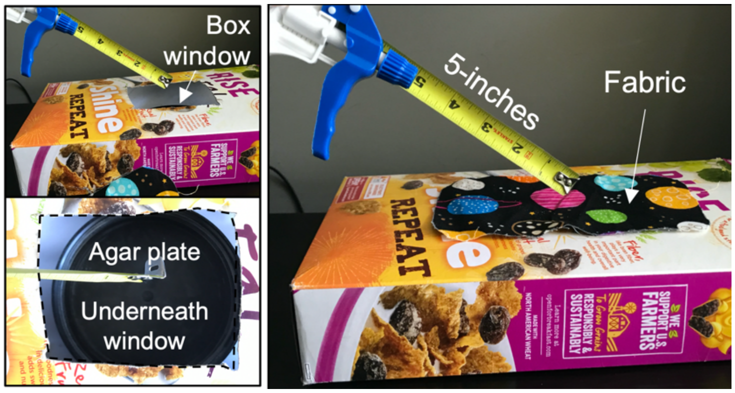

13. Après 24 heures, vérifiez les différents gel en portant votre masque. Comptez le nombre de colonies (de petits points de pousse) sur la surface du gel, sans les toucher

3ème Expérience, suite

Conclusion:

1. Notez vos résultats dans le tableau ci-dessous.
2. Lavez-vous les mains après avoir observé les gels.
3. Répétez les observations après 48 heures. Comptez le nombres de colonies.
4. Débarrassez-vous des gel en les jetant à la poubelle, nettoyer soigneusement la zone et lavez-vous les mains..

Partager vos résultats: Rentrez vos résultats de la colonne G ici: https://forms.gle/5q7V44nbT8fu6Jvz6
https://docs.google.com/forms/d/e/1FAIpQLSd9cV7HQzxr49MsC-icHCzxIOnlhX2z7e7iza3cJ-NGzJaFRw/viewform

Tableau 2. **Entrez ci-dessous les résultats de l'expérience** 3.
CFU: nombre de colonies sur la gél

	A	B	C	D	E	F	G
**Gel**	Nombres de Colonies à heure zéro	Nombres de Colonies après 24 heures	Nombres de Colonies après 48 heures	Diamètre du gel en cm	Rayon du gel en cm (diamètre divisé par deux)	Aire du gel = pi x r^2= (3.14 x rayon x rayon) en cm^2	**Colonies/aire^2 (colonne C divisé par colonne F)**
**0**	0						____=Baseline G
**1**	0						
**2**	0						
**3**	0						

Répondez aux questions suivantes.

Q3.1 Qu'avez vous observé?

Q3.2 Esrt-ce qu'une protection avec une épaisseur est efficace pour protéger les transmissions de microorganismes?

Q3.3 Est-ce une protection avec deux épaisseurs est-elle autant ou plus efficace qu'une protection avec une seule épaisseur?

Q3.4 Les deux types de masques peuvent-elles contenir tout les germes provenant d'un éternuement?

Q3.5 Qu'est ce qui est idéal pour éviter la transmission de germes?

Partager vos résultats en ligne à: https://forms.gle/5q7V44nbT8fu6Jvz6 ou scannez le code QR ci-dessous.

Conclusion #FaceMaskChallenge:

- Une seule épaisseur de tissu bloque la transmission de germe mais ce n'est pas l'idéal.
- Deux épaisseur à l'inverse est beaucoup mieux!
- Toutefois cela ne bloque pas toute les gouttelettes transportant les microbes il est donc très important de se laver les mains.
- **Puisque les microbes sont bloquer dans votre masques il est important de la nettoyer régulièrement!**

4ème Expérience: Quelle quantité de bactérie je transmets lorsque je parle ?

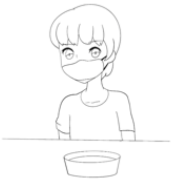

Notion fondamentale: On produit tous des gouttelettes de germes lorsque l'on parle.

Difficulté: Expert.

Découverte: Un masque stoppe la contamination de l'environnement par notre salive.

Objectif du module: En parlant avec ou sans un masque et en les récupérant sur un gel prévu pour la pousse de microorganismes il sera possible de déterminer l'efficacité des masques pour stopper la transmission de germes.
Durée: 36-48 heures.

Matériels pour l'incubation
(à préparer à l'avance)

- 2 gélatine pour la pousse des germes (l'expérience 3)
- Une boîte de céréales vide
- Un masque en tissus

Protocole:

Partie A. Préparez la gélatine pour la pousse des germes – voir **Expérience n° 3** Partie A.

Partie B. L'Experience (pour déterminer quels quantité de goutelettes ont produits lorsque l'on parle à voix haute):

1. Téléchargez l'application gratuite CDC Noise App sur smartphone, avec celui-ci ajuster le niveau de décibels à 70.
2. Légendez deux gels, une appelée gel sans masque, le seconde gel avec masque.
3. Préparez la boîte pour déposer les récipients avec les gels, juste après avoir reçu le spray. Ce sera la chambre d'incubation.
4. Déposer le gel sans masques à 20 cm de vous.
5. Buvez une gorgée d'eau et prononcez le texte suivant le plus clairement possible, comme si vous parliez à quelqu'un de l'autre côté de la salle.

« Je suis un scientifique. Je vais lire ce passage à voix haute en tant qu'expérience pour comprendre l'importance des masques et protection en tissus. Je veux voir la quantité de gouttelettes qui peuvent contaminer l'environnement sont produites lorsque je comptes jusqu'à 100 [compter jusqu'à 100.] Merci et tout mes vœux de santé !"

6. **Protéger votre visage avec un masques réutilisable à deux épaisseur**.
7. Déposé le gel avec masques dans la même position que l'étape 3.
8. **Répétez l'étape 4** .
9. Déposé soigneusement le gel avec masque dans la chambre d'incubation. Fermez la boite et laissez-le dans un endroit chaud où il peut rester quelques jours sans être bouger (le dessus d'un frigo par exemple).

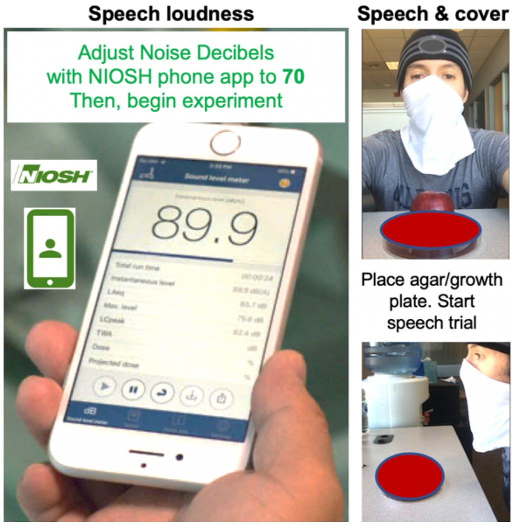

10. Après 24 heures, vérifiez les différents gel en portant votre masque. Comptez le nombre de colonies(de petits points de pousse) sur la surface du gel, sans les toucher.

4ème Expérience, suite

Conclusion:

1. Notez vos résultats dans le tableau ci-dessous.
2. Lavez-vous les mains après avoir observé les gels.
3. Répétez les observations après 48 heures. Comptez le nombres de colonies.
4. Débarrassez-vous des gel en les jetant à la poubelle, nettoyer soigneusement la zone et lavez-vous les mains..
   Partager vos résultats: Rentrez vos résultats de la colonne G ici: https://forms.gle/5q7V44nbT8fu6Jvz6

Table 3. Enter below the Results of Experiment 4

	A	B	C	D	E	F	G
**Gel**	Nombre de Colonies à l'heure zéro.	Nombre de Colonies après 24 heures	Nombre de Colonies après 48 heures	D Diamètre du gel en cm	Rayon du gel (La moitié du résultat de la colonne D) en cm	Aire du gel= pi x r^2= (3.14 x rayon x rayon) En cm^2	Colonies /cm^2 (colonne C divisé par la colonne F)
**4**	0						____=Baseline G
**5**	0						

Répondez aux questions suivantes.

Q4.1 *Qu'avez-vous observez?* ______________________

Q4.2 *Est-ce que les masques avec deux épaisseur est plus efficace pour prévenir la transmissions de germes??*_____________________

Q4.3 *Est-ce que le tissus contient tout les microbes?* _____________________

Q4.4 *Que peut-on faire d'autre pour maintenir une bonne hygiène ?* _____________________

Partager vos résultats ici: https://forms.gle/5q7V44nbT8fu6Jvz6 ou scannez le code QR ci-dessous.

Conclusion #FaceMaskChallenge:

- On transmet une grande partie de microbes simplement en parlant – Il faut toujours garder cela en tête pour l'application efficace des distances sociales
- La solution afin de prévenir le plus possible le COVID ou d'autres infection et de porter un masque et ce laver les mains régulière afin de maintenir une bonne hygiène. Faites passer le mot ! Imprimer et afficher ces rappels:

https://figshare.com/articles/Door_Signs_to_Promote_Public_Droplet_Safety_Amidst_COVID-19/12202808/1

Pour les enseignants: l'application téléphonique illustrée dans la figure de la page 10 et les détails scientifiques de l'expérience 4 sont décrits dans: https://doi.org/10.3389/fmed.2020.00504

Ressources complémentaires

Glossaire

Aérosol: Dispersion en particules très fines d'un liquide, d'une solution ou d'un solide dans un gaz.
Colonie: un cluster de bacterie assez large pour être visible à l'oeil nu.
Gouttelettes: petite goutte de liquide.
Epidémie: Apparition et propagation d'une maladie infectieuse contagieuse qui frappe en même temps et en un même endroit un grand nombre de personnes.
Masque en tissus: Toutes pièces en tissus placé sur la bouche et le nez pour prévenir de toute contamination respiratoires.
Masque chirurgicaux: équipement de protection personnel, qui réduit les infections dans le personel soignants et patiennts.
Pandémie: Epidémie qui atteint un grand nombre de personnes, dans une zone géographique très etendue.
Virus: une particule aussi petite que 20 nanomètre qui peut entrer et infecter les cellules. Elles sont capables de créer jusqu'à 1000 nouveau virus en détruisant la cellule-hôte et infecte d'autre hôte.

Ressources

Les questions fréquentes sur le COVID-19 par OMS. https://www.who.int/fr/emergencies/diseases/novel-coronavirus-2019
Face Mask Challenge Citizen Science module https://sites.google.com/kent.edu/face-mask-challenge-fr/face-mask-challenge-home-fr
Encyclopedia Britannica COVID-19 https://www.britannica.com/explore/savingearth/covid-19/
The Scale of Things https://www.nano.gov/nanotech-101/what/nano-size
Research on face covers and germ transmission https://doi.org/10.3389/fmed.2020.00260
Face Mask Challenge Citizen Science (fr) https://forms.gle/5q7V44nbT8fu6Jvz6
Printable Door sign reminders to encourage wearing face masks
https://figshare.com/articles/Door_Signs_to_Promote_Public_Droplet_Safety_Amidst_COVID-19/12202808/1
NIOSH Sound Level Meter App: https://www.cdc.gov/niosh/topics/noise/app.html

---

Authors: Sarah E. Eichler[1], Austin P. Hopperton[2], Juan Jose Alava[3], Antonio Jr. Pereira[4], Rukhsana Ahmed[5], Zisis Kozlakidis[6], Sanja Ilic[7] & Alex Rodriguez-Palacios[2,8]

Cartoonist: Maddie Rosemark

Affiliations: [1]Department of Biological Sciences, Kent State University, USA; [2]Division of Gastroenterology and Liver Disease, Case Western Reserve University School of Medicine, USA; [3]Institute for the Oceans and Fisheries, The University of British Columbia, Canada; [4]Institute of Technology, Federal University of Pará, Brazil; [5] Department of Communication, University at Albany, SUNY, USA; [6]International Agency for Research on Cancer, World Health Organization, Lyon, France; [7]Department of Human Sciences Human Nutrition, The Ohio State University, USA; [8]University Hospitals Research and Education Institute, University Hospitals Cleveland Medical Center, Cleveland, OH, USA.

Translations: Juan Jose Alava, Diana Maria Rodriguez, Diego A. Pulido, Zisis Kozlakidis, Antonio Jr. Pereira, Alex Rodriguez-P & Maissa Zeghidi.

Open CC BY license http://creativecommons.org/licenses/by/4.0/

Versions en quatre langues supplémentaires (Spanish, English, French, Portuguese) font partie de la soumission initiale et sont liés via https://sites.google.com/kent.edu/face-mask-challenge/face-mask-challenge-home

Archive - Contactez les auteurssi suggestions/questions. Un référentiel a été créé pour l'archive du dernier auteur post-publication approuvé versions de module et pour les contributions de la communauté: **https://github.com/axr503/education**. Alex Rodriguez-Palacios, Assistant Professor, School of Medicine, axr503@case.edu.

International collaboration of scientists & educators from

Module version 1.0 (August 20/2020)

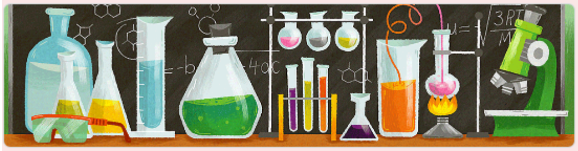

(French) Partager vos résultats sur les différents moyen de protection pour l'hygiène respiratoire: Devenez un scientifique citoyen!

Partagez de façon anonyme vos résultats de vos expériences fait maison.

Quel et l'objectif de ce questionnaire ?:
Il y aura 4 questions sur votre lieu d'habitation, votre âge qui ne prends que 1 à 2 minutes à compléter:
Il n'y a pas de risques due à ce questionnaire. Toutes réponses est à votre bon vouloir et anonyme. Il n'y aucune compensation financière liée à la participation.

Puisque certaines expériences prennent plus d'un jour pour être compéter il est recommandé de noter tous vos résultats sur la feuilles d'instruction et de soumettre tout vos résultats en une seule fois. A la fin de chaque questionnaire d'une expérience une option existe pour remplir les résultats d'une autre expérience ou terminer le questionnaire directement.

En continuant ce questionnaire, vous consentez au partage d'information anonyme et inidentifiable.
Vous pouvez revoir les instructions du projet ici:
ttps://sites.google.com/kent.edu/face-mask-challenge/face-mask-challenge-home

Next — Page 1 of 8

(French) Partager vos résultats sur les différents moyen de protection pour l'hygiène respiratoire: Devenez un scientifique citoyen!

* Required

Information personnel

#FaceCoverChallenge

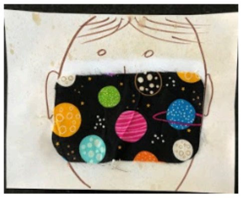

En cliquant le bouton « Oui, j'accepte » cela indique que vous avez bien lu le texte ci-dessus, accepte de participer et que vous ayez 18 ans ou plus. Vous serez dirigé directement au questionnaire.Si vous ne souhaitez pas participer, cliquer le bouton « Non je ne souhaites pas participer à la soumission de résultats » ou fermez simplement la fenêtre : *

- ○ Oui, j'accepte de participer et de partager mes résultats . (Cliquer sur NEXT pour continuer)
- ○ Non, je ne souhaites pas participer (Cliquer sur NEXT pour continuer)

Est-ce votre première soumissions de la Science Citoyenne? *

Choose ▾

Back Next — Page 2 of 8

(French) Partager vos résultats sur les différents moyen de protection pour l'hygiène respiratoire: Devenez un scientifique citoyen!

* Required

Information personnel

Nous souhaitons savoir à propos de vous!

Pays *

Choose

Quel âge a le scientifique citoyen qui a dirigé les expériences? *

Your answer

Quel âge à la personne la plus vieille vivant chez vous? (si vous vivez seule répondez la même réponse que précédemment.) *

Your answer

Quel est le niveau scolaire du scientifique citoyen?

Choose

Back Next Page 3 of 8

(French) Partager vos résultats sur les différents moyen de protection pour l'hygiène respiratoire: Devenez un scientifique citoyen!

Choisissez votre expérience

Choisissez par quoi vous souhaitez commencer.

- (•) Expérience 1
- ( ) Expérience 2
- ( ) Expérience 3
- ( ) Expérience 4

Clear selection

Back | Next | Page 4 of 8

(French) Partager vos résultats sur les différents moyen de protection pour l'hygiène respiratoire: Devenez un scientifique citoyen!

Experiment 1 -How far can droplets travel?

Exp.1-Q1/7. Sans tissus protecteur: Lorsque le flacon vaporisateur était à plat, quel était le numéro du papier le plus éloigné touché ? (0- Absence de gouttelettes vues.)

	0	1	2	3	4	5	6	7	
Le plus proche du flacon	○	○	○	○	○	○	○	○	Le plus loin du flacon

Exp.1-Q2/7. - Sans tissus protecteur: Lorsque le flacon vaporisateur était à plat comment qualifier vous la quantité de gouttelettes sur le papier numéro 3?

- ( ) Faible (0-10)
- ( ) Moyen (11-100)
- ( ) Fort (100-500)
- ( ) Très fort (500+)

Exp.1-Q3/7. - Sans tissus protecteur: Lorsque le flacon vaporisateur était dirigé vers le haut quel était le numéro du papier le plus éloigné touché ? (0- Absence de gouttelettes vues.)

	0	1	2	3	4	5	6	7	
Le plus proche du flacon	○	○	○	○	○	○	○	○	Le plus loin du flacon

Exp.1-Q4/7. - Sans tissus protecteur: Lorsque le flacon vaporisateur était dirigé vers le haut comment qualifier vous la quantité de gouttelettes sur le papier numéro 3?

- ◯ Faible (0-10)
- ◯ Moyen (11-100)
- ◯ Fort (100-500
- ◯ Très fort (500+)

Exp.1-Q5/7. - Sans tissus protecteur: Lorsque le flacon vaporisateur était dirigé vers le bas quel était le numéro du papier le plus éloigné touché ? (0- Absence de gouttelettes vues.)

	0	1	2	3	4	5	6	7	
Le plus proche du flacon	◯	◯	◯	◯	◯	◯	◯	◯	Le plus loin du flacon

Exp.1-Q6/7. - Sans tissus protecteur: Lorsque le flacon vaporisateur était dirigé vers le bas comment qualifier vous la quantité de gouttelettes sur le papier numéro 3?

- ◯ Faible (0-10)
- ◯ Moyen (11-100)
- ◯ Fort (100-500)
- ◯ Très fort (500+)

Exp.1-Q7/7 – Avec quelle position le flacon pouvait-il faire un spray au plus loin?

- ◯ Flacon à plat
- ◯ Flacon dirigé vers le haut
- ◯ Flacon dirigé vers le bas
- ◯ Autre

Voulez vous soumettre des résultats d'une autre expérience ?

- ◯ Expérience 2
- ◯ Expérience 3
- ◯ Expérience 4
- ◯ Terminer et soumettre

Back Next Page 5 of 8

Expérience 2 - Quel est l'efficacité d'une barrière protectrice en tissus contre les gouttelettes ?

Exp.2-Q1/3. – Quel type de tissus avez-vous utilisé ? (Vous pouvez choisir plusieurs options.)

- ☐ Coton
- ☐ Polyester
- ☐ Poly coton
- ☐ Soie
- ☐ Laine
- ☐ Serviette
- ☐ Echarpe
- ☐ T-shirt
- ☐ Matière élastique
- ☐ Autre

Exp.2-Q2/3. – Avec barrière textile : quel était le numéro du papier le plus éloigné touché ? (0- Absence de gouttelettes vues.)

	0	1	2	3	4	5	6	7	
Le plus proche du flacon	○	○	○	○	○	○	○	○	Le plus loin du flacon

Exp.2-Q3/3. - Avec barrière textile: comment qualifier vous la quantité de gouttelettes sur le papier numéro 3 ?

- ○ Faible (0-10)
- ○ Moyen (11-100)
- ○ Fort (100-500)
- ○ Très fort (500+)

Voulez-vous soumettre des résultats d'une autre expérience ?

- ○ Expérience 1
- ◉ Expérience 3
- ○ Expérience 4
- ○ Terminer et soumettre

Clear selection

Back Next Page 6 of 8

Expérience 3 - Quelle quantité de gouttelettes transportant des germes peuvent passer à travers deux-couches de tissus?

Exp.3-Q1/3.- Gel# 0 Sans tissus résultat de la colonne G:

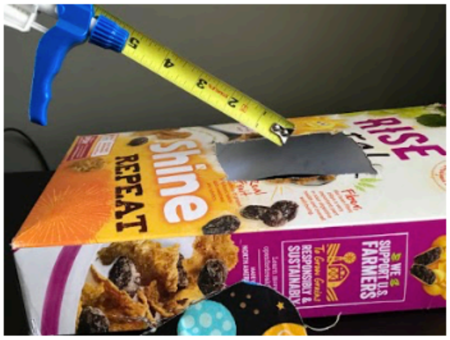

Your answer

Exp.3-Q2/3.- Gel# 1 Une épaisseur de tissus résultat de la colonne G:

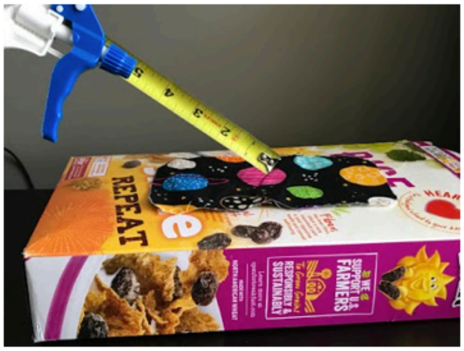

Your answer

Exp.3-Q3/3.- Gel# 2 Deux épaisseurs de tissus résultat de la colonne G:

Your answer

Voulez-vous soumettre des résultats d'une autre expérience?

- ○ Expérience 1
- ○ Expérience 2
- ◉ Expérience 4
- ○ Terminer et soumettre

Clear selection

Back Next Page 7 of 8

Expérience 4 - Quelle quantité de bactérie je transmets lorsque je parle?

Exp.4-Q1/2.- Gel sans masques résultats de la colonne G

Your answer

Exp.4-Q2/2.- Gel avec masques résultats de la colonne G

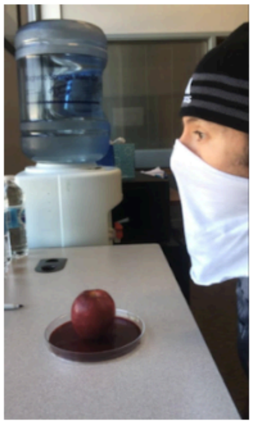

Your answer

Voulez-vous soumettre des résultats d'une autre expérience?

- ○ Expérience 1
- ○ Expérience 2
- ○ Expérience 3
- ◉ Terminer et soumettre

Clear selection

Back | Next | Page 8 of 8

Would you like to go back to another experiment or finish and submit?

- ○ Experiment 1
- ○ Experiment 2
- ○ Experiment 4
- ◉ Submit and Finish

Clear selection

Back | Next | Page 8 of 8

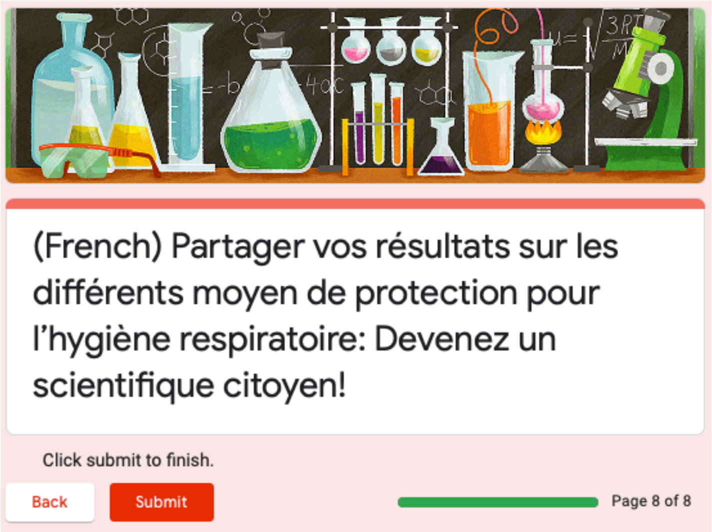
(French) Partager vos résultats sur les différents moyen de protection pour l'hygiène respiratoire: Devenez un scientifique citoyen!
Click submit to finish.
Back
Submit
Page 8 of 8

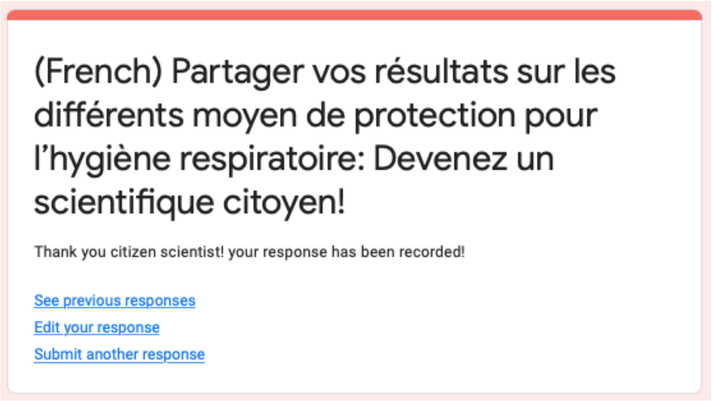
(French) Partager vos résultats sur les différents moyen de protection pour l'hygiène respiratoire: Devenez un scientifique citoyen!
Thank you citizen scientist! your response has been recorded!
See previous responses
Edit your response
Submit another response

COLLECTIVE RESULTS

(below is a mock series or random responses for illustration)
Please only enter real results so we all could see the real findings.
If some results do not resemble your data, discuss with a teacher.
And ask/read the real laboratory experiment results that were obtained in a Medical Research Center.

Original studies are published here:

https://doi.org/10.3389/fmed.2020.00260 https://doi.org/10.3389/fmed.2020.00504

Textile Masks and Surface Covers—A Spray Simulation Method and a "Universal Droplet Reduction Model" Against Respiratory Pandemics. Rodriguez-Palacios A, Cominelli F, Basson AR, Pizarro TT, and Ilic S. (2020) Front. Med. 7:260. doi: 10.3389/fmed.2020.00260

Germ-Free Mice Under Two-Layer Textiles are Fully Protected From Bacteria in Sprayed Microdorplets: A Functional in-vivo Test Strategy of Facemasks and Filtration Materials. Rodriguez-Palacios A, Conger M, and Cominelli F. (2020) Front. Med. 7:504. doi: 10.3389/fmed.2020.00504

A Citizen Science Facemask Experiment and Educational Modules to Increase Coronavirus Safety in Communities and Schools. Eichler SE, Hopperton AP, Alava JJ, Pereira A, Ahmed R, Kozlakidis Z, Ilic S and Rodriguez-Palacios A (2020) Front. Med. 7:486. doi: 10.3389/fmed.2020.00486

https://doi.org/10.3389/fmed.2020.00486

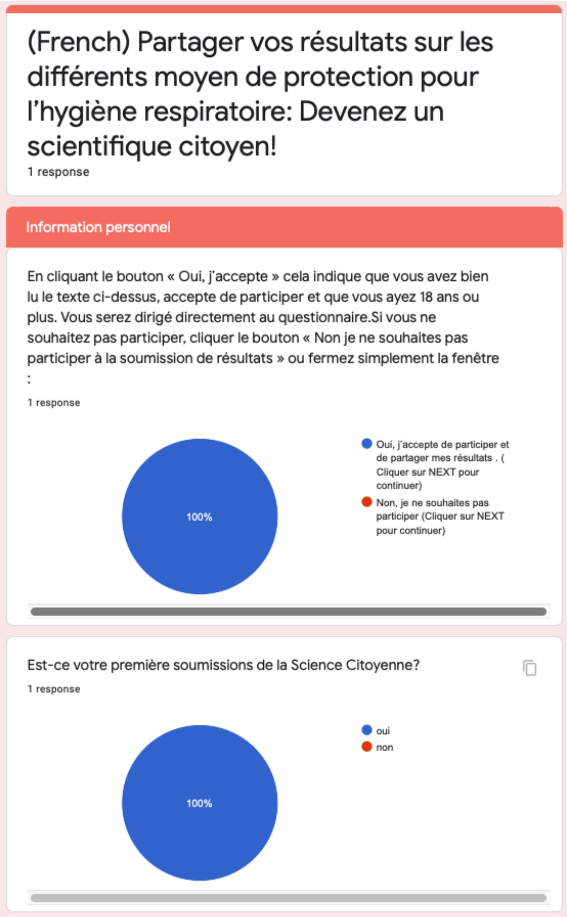

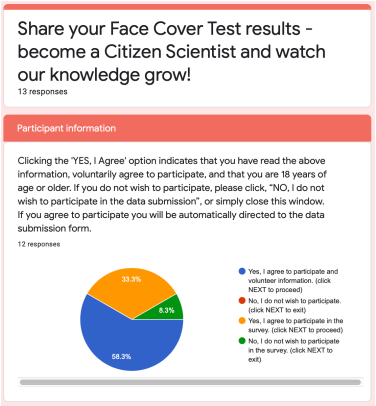

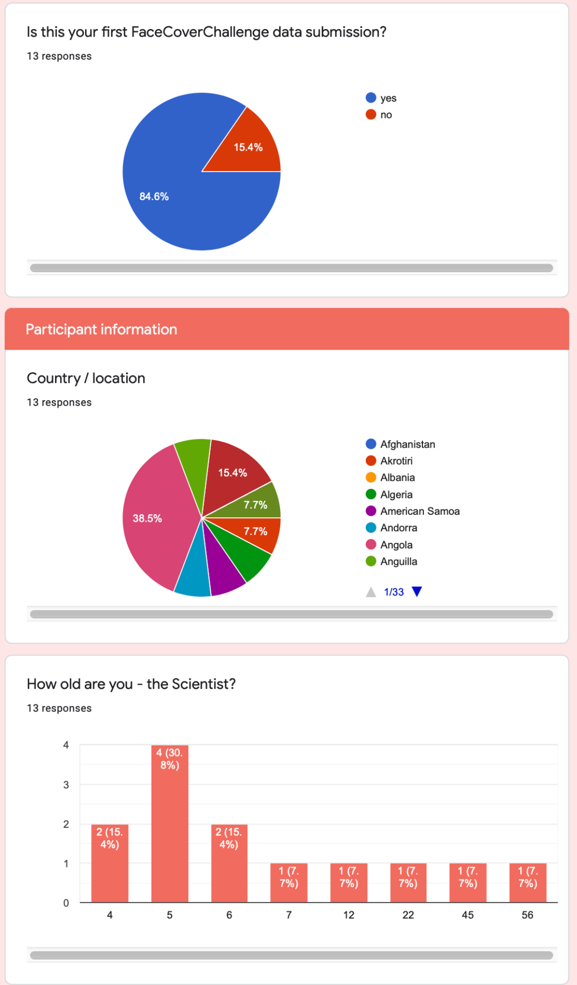

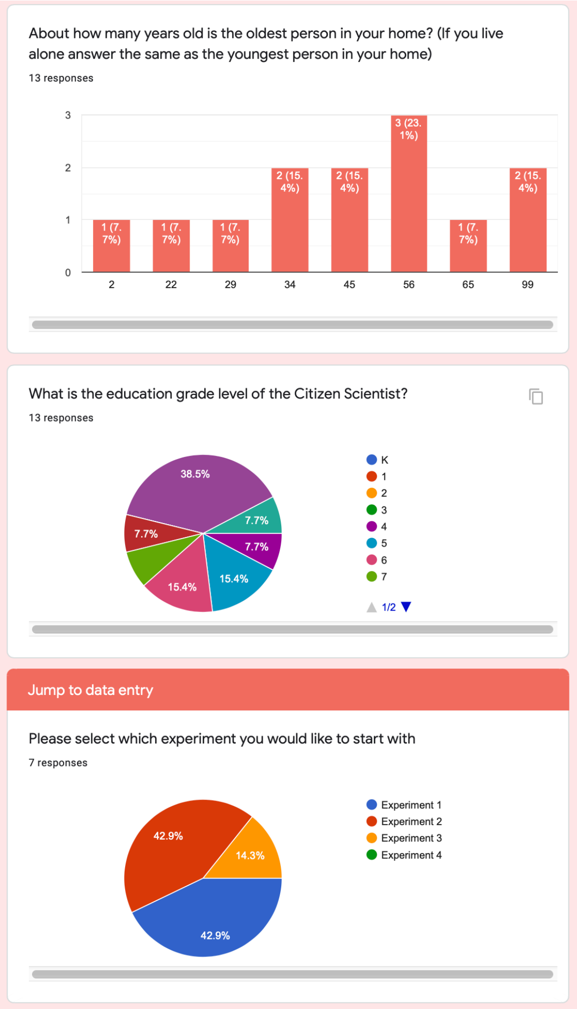

See remaining results in response form link
https://forms.gle/5q7V44nbT8fu6Jvz6

A Citizen Science Facemask Experiment and Educational Modules to Increase Coronavirus Safety in Communities and Schools. Eichler SE, Hopperton AP, Alava JJ, Pereira A, Ahmed R, Kozlakidis Z, Ilic S and Rodriguez-Palacios A (2020) Front. Med. 7:486. doi: 10.3389/fmed.2020.00486
SUPPLEMENTARY MODULE - In English.
Provided by Alex Rodriguez-P. axr503@case.edu.
V1 (August 20, 2020) with the accepted publication.
Results submission form in English:
https://docs.google.com/forms/d/e/1FAIpQLSd9cV7HQzxr49MsC-icHCzxIOnlhX2z7e7iza3cJ-NGzJaFRw/viewform

Partager vos résultats ici en
Anglais: https://bit.ly/facemaskchallengedata, ou en
Français: https://forms.gle/5q7V44nbT8fu6Jvz6
Espanol: https://forms.gle/iWgE8JFeDvezxXTq6
Portugues: https://forms.gle/EJHauCWTdDbny66N8

frontiers in Medicine

OPINION
published: xx July 2020
doi: 10.3389/fmed.2020.00486

Check for updates

A Citizen Science Facemask Experiment and Educational Modules to Improve Coronavirus Safety in Communities and Schools

Sarah E. Eichler[1], *Austin P. Hopperton*[2], *Juan José Alava*[3], *Antonio Pereira Jr.*[4], *Rukhsana Ahmed*[5], *Zisis Kozlakidis*[6], *Sanja Ilic*[7] *and Alexander Rodriguez-Palacios*[2]*

[1] Department of Biological Sciences, Kent State University at Salem, Salem, MA, United States, [2] Division of Gastroenterology and Liver Disease, School of Medicine, Case Western Reserve University, Cleveland, OH, United States, [3] Institute for Oceans and Fisheries, University of British Columbia, Vancouver, BC, Canada, [4] Institute of Technology, Federal University of Pará, Belém, Brazil, [5] Department of Communication, University at Albany, SUNY, Albany, NY, United States, [6] International Agency for Research on Cancer, World Health Organization (France), Lyon, France, [7] Department of Human Sciences, The Ohio State University, Columbus, OH, United States

Keywords: COVID-19, mass media, health communication, prevention, intervention, social behavioral changes, facemask, school education

OPEN ACCESS

SUPPLEMENTARY MATERIALS

Education Module #1 ENGLISH
Education Module #2 SPANISH
Education Module #3 FRENCH
Education Module #4 PORTUGUESE

Education Module

PORTUGUESE

Um Experimento Cientista-Cidadão com Máscaras Faciais

Campanha Educativa e Módulos para Promover Medidas de Proteção ao Coronavírus em Comunidades e Escolas

COVID-19 é a abreviação da Doença do Coronavirus, que é causada pelo vírus SARS-CoV-2. Como outros vírus, o SARS-CoV-2 é muito pequeno para ser visto a olho nu. No entanto, o SARS-CoV-2 espalha a doença COVID-19 principalmente **através de fluidos orais e nasais ou gotículas dispersas no ar**. COVID afeta mais seriamente os idosos e os que já estão doentes, causando falta de ar, febre, tosse, dores musculares, e inflamação sistêmica que pode levar à hospitalização.

Tendo em vista que a COVID-19 se espalha de pessoa para pessoa através de saliva e microgotículas nasais, os profissionais de saúde recomendam manter uma distância de pelo menos 1,8 metros de outras pessoas, limitar a exposição em grupos, lavar as mãos e usar máscaras. Como as máscaras são escassas em alguns lugares, muitas pessoas têm usado outros revestimentos faciais, como lenços e panos. **A principal função das máscaras** e revestimentos faciais não é filtrar o ar que se respira, mas **limitar a propagação de saliva e microgotículas nasais que produzimos e espalhamos naturalmente quando falamos, espirramos e tossimos**.

Esta campanha educativa foi preparada para informar as comunidades sobre a dinâmica de espalhamento de gotículas e testar a utilidade de coberturas faciais. **Em quatro experimentos caseiros simples** você pode quantificar e observar como usar revestimentos faciais pode reduzir a propagação de gotículas para manter sua família e seus amigos seguros.

Este módulo é baseado em estudos científicos realizado em um centro de pesquisa médica universitário. Para mais informações:

https://doi.org/10.3389/fmed.2020.00260

Textile Masks and Surface Covers—A Spray Simulation Method and a "Universal Droplet Reduction Model" Against Respiratory Pandemics. Rodriguez-Palacios A, Cominelli F, Basson AR, Pizarro TT, and Ilic S. (2020) Front. Med. 7:260. doi: 10.3389/fmed.2020.00260

https://doi.org/10.3389/fmed.2020.00504

Germ-Free Mice Under Two-Layer Textiles are Fully Protected From Bacteria in Sprayed Microdroplets: A Functional in-vivo Test Strategy of Facemasks and Filtration Materials. Rodriguez-Palacios A, Conger M, and Cominelli F. (2020) Front. Med. 7:504. doi: 10.3389/fmed.2020.00504

Ao utilizar esse módulo resultante de uma colaboração internacional, por favor mencione estes dois estudos acima, o módulo, e utiliza essa **Citação**:

A Citizen Science Facemask Experiment and Educational Modules to Increase Coronavirus Safety in Communities and Schools. Eichler SE, Hopperton AP, Alava JJ, Pereira A, Ahmed R, Kozlakidis Z, Ilic S and Rodriguez-Palacios A (2020) Front. Med. 7:486. doi: 10.3389/fmed.2020.00486

https://doi.org/10.3389/fmed.2020.00486

Este módulo educacional apresenta uma introdução sobre gotículas respiratórias e a importância de coberturas faciais na prevenção da propagação de micro-organismos respiratórios.

Em uma série de quatro experimentos você vai quantificar como as coberturas faciais ajudam a controlar doenças respiratórias que são transmitidas por microgotas orais e nasais de saliva/ muco que produzimos quando falamos, espirramos ou tossimos.

Experimento 1- Até onde as gotículas líquidas pulverizadas podem viajar?

Experimento 2 - Quão bem uma barreira de pano para de pulverizar gotículas?

Experimento 3 - Quantas gotículas que carregam micro-organismos podem atravessar duas camadas de pano?

Experimento 4 - Quantos micro-organismos saem da minha boca enquanto falo?

Você pode compartilhar seus resultados e ver os resultados de outras pessoas aqui em **Ingles** https://bit.ly/facemaskchallengedata, ou **Portugues** https://forms.gle/EJHauCWTdDbny66N8

Os dados compartilhados são anônimos!
Nenhuma informação pessoal será coletada!

Este também está disponível em **outros idiomas** (p.ex. **Inglês**, **Espanhol**, **Francês**) em la **Citation** em https://bit.ly/facemaskchallenge (website) & aqui **https://github.com/axr503/education**

Se você é professor e gostaria de nos informar que está implementando o módulo em sua escola / sala de aula: https://forms.gle/Sg36k3HceMos1Xpb8

Contact: Alex Rodriguez-P. axr503@case.edu

International collaboration of scientists & educators from

School of Medicine Case Western Reserve University · University Hospitals · Kent State University · The Ohio State University College of Education and Human Ecology · LMIC International Agency for Research on Cancer/WHO · BCNet · University at Albany State University of New York · Universidade Federal do Pará · The University of British Columbia

INTRODUÇÃO À ATIVIDADE DE CIÊNCIA CIDADÃ

Cientistas Cidadãos farão simulações com nuvens de gotículas pulverizadas usando líquidos domésticos seguros. O projeto consiste em experimentos práticos simples usando uma garrafa de pulverizador e outros itens que você encontra em sua casa. Como outros cientistas, você compartilhará seus dados e, provavelmente, confirmará a hipótese de que usar um protetor facial pode reduzir a propagação de micro-organismos e ajudar a proteger sua família e amigos. Quatro experimentos são descritos neste módulo – você pode fazer quantos quiser usando ingredientes alimentares básicos, materiais de cozinha e alguns objetos reciclados, como listado. As atividades consistirão em medir quantas gotas de um líquido pulverizado podem passar através do material da cobertura facial, e quão distante as gotículas líquidas podem se projetar a partir de um espirro simulado.

Os cientistas cidadãos aprenderão em primeira mão sobre como as microgotas podem causar contaminação, como as coberturas faciais funcionam e aprenderão também a importância de usar uma cobertura facial durante uma pandemia. Se você optar por compartilhar seus dados, você fará parte de um projeto científico global para ajudar a entender a transmissão e a prevenção de doenças – um verdadeiro Cientista Cidadão! Compartilhe seus resultados em Ingles, (https://bit.ly/facemaskchallengedata) ou em Portuges https://forms.gle/EJHauCWTdDbny66N8

Público: Recomendado para Professores e Pais de alunos a partir da terceira série do ensino fundamental. Adequado para todas as idades com supervisão, se necessário.

Materiais básicos

- 2 xícaras de líquido de cor escura (bebida esportiva, suco de uva, café, refrigerante).
- 1 frasco pulverizador.
- 28 folhas de papel branco tamanho A4 ou Carta.
- 1 fita métrica ou régua.
- 2 caixas de cereal vazias.
- Pedaço de tecido/pano (pelo menos 25x25 cm) (pode ficar manchado!) tal como toalha, fronha, camiseta, cachecol, bandana, lenço, guarda-roupa, guardanapo de pano etc.
- fita adesiva.
- toalhas de papel para limpeza.
- Opcional: papel quadriculado.

Materiais de placas de incubação
(avançado, prepare-se com antecedência)

- 1 caixa de cereal vazia.
- Recipientes rasos ou forminhas de *cupcake* de alumínio (6-10).
- 1 colher de açúcar.
- 1 cubo de caldo de caldo de carne ou similar.
- 2 pacotes de 7 gramas de gelatina comum (vermelha).
- 1 xícara de água.
- micro-ondas e recipiente de vidro (355 ml), ou panela com tampa e fogão.
- sacos de plástico transparente tipo zip-lock.
- cobertura facial (máscara) de pano (limpa) (deve cobrir o nariz e a boca).

https://www.instructables.com/id/Homemade-Nutrient-Agar/

Assista esse video da Nature sobre dinâmica dos fluidos:
A Física do Espirro
(Maio 2016, YouTube)

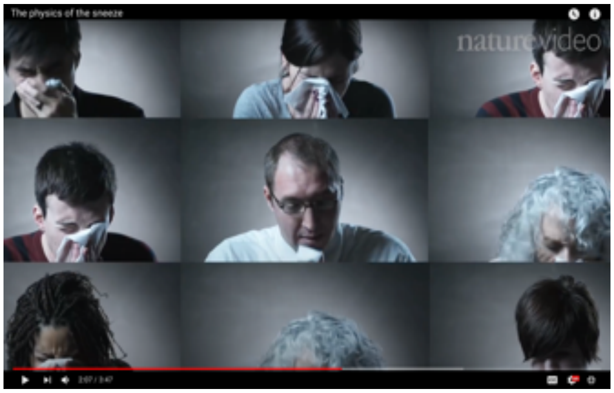

Em preparação para os experimentos, visualize um espirro em câmera lenta (minuto 2:00)
https://youtu.be/bFxgVksID-k?t=107

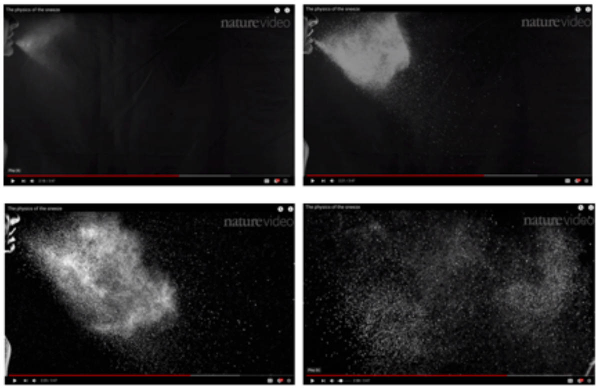

Tente replicar esta nuvem de gotículas com as configurações do seu frasco de spray!

Experimento 1
Até onde as gotículas líquidas podem alcançar?

Conceito principal: Nuvem de gotículas pulverizadas contem vários tamanhos de gotículas: Macro (grande, visível) versus Micro (pequena, difícil de ver).

Grau de Dificuldade: Fácil.

Nova Descoberta: Gotículas grandes podem alcançar mais longe do que a gotículas menores em um "espirro" simulado.

Objetivo de Aprendizagem: Avaliar o espalhamento de gotículas em um cenário simulado de pulverização infecciosa.

Duração: aproximadamente 20 minutos.

Materiais

- 1 frasco pulverizador.
- Líquido de cor escura (refrigerante, café frio, ou bebida esportiva) em quantidade suficiente para encher um frasco pulverizador até a metade.
- 21 folhas de papel branco ou quadriculado tamanho A4 ou carta.
- Um grande espaço vazio em uma mesa ou chão lavável.
- Caneta ou lápis

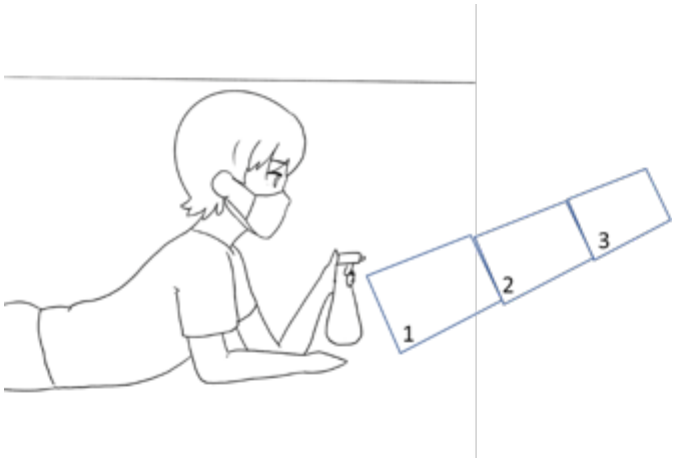

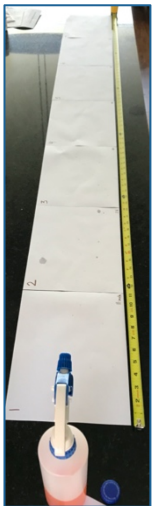

Procedimento

Parte A - Garrafa pulverizadora plana no chão:

1. Coloque 7 folhas de papel no chão, com as bordas maiores alinhadas, numerando-as de 1-7 (1 é mais próximo do frasco pulverizador, 7 é mais distante, como mostrado no diagrama).
2. Encha o frasco pulverizador com qualquer tipo de líquido escuro e seguro, como café, suco de uva ou refrigerante de cola. Teste seu frasco pulverizador sobre uma pia e ajuste-o para que ela produza uma névoa fina com gotículas de tamanho médio (veja vídeo na página 2 como guia). Uma vez que o padrão de pulverização esteja definido, não o altere.
3. Posicione o frasco pulverizador em frente da primeira folha de papel e mire a garrafa para o centro da trilha de papel enquanto a parte inferior da garrafa permanece apoiada no chão.
4. Dê uma pulverizada completa. Aguarde 30 segundos para que as gotículas aterrissem e observe as manchas no papel.
5. Registre seus resultados na **tabela 1** abaixo (dê suas respostas para a condição 'frasco **PLANO**' na próxima página e prossiga para a **PARTE B**).

Experimento 1, continuar

Parte B – Frasco pulverizador em Diferentes Ângulos

1. Inicie um novo experimento substituindo e renumerando as folhas como na parte A.
2. Usando o mesmo frasco pulverizador da Parte A, incline o frasco com o bocal para **cima** (cerca de 10 graus) usando um lápis apoiado sob o frasco. Faça uma pulverização completa e registre seus resultados na **Tabela 1** abaixo (veja as perguntas para '**Ângulo para CIMA**') e prossiga para a próxima etapa.
3. Inicie um novo experimento substituindo e renumerando as folhas como na parte A.
4. Repita o mesmo procedimento, agora com o frasco inclinado para **baixo** (de novo com a ajuda de um lápis). Registre seus resultados na **Tabela 1** abaixo (veja as perguntas para '**Ângulo para BAIXO**').
5. **Você finalizou o experimento 1**! Vá para a **Tabela 1** e **compartilhe seus resultados *online***.

Responda as Seguintes Perguntas na Tabela 1 abaixo para cada experimento

Q1.1 *Como você classificaria o número de gotículas no terceiro papel* ? (Use a escala abaixo)

BAIXO 0-10	MÉDIO 0-10	ALTO 0-10	MUITO ALTO 0-10

Q1.2 *Qual posição do frasco (plano, inclinado para cima ou inclinado para baixo) projetou gotículas mais distantes?* ______________________

Q1.3 *As gotículas mais distantes eram grandes ou pequenas?* ______________________

Tabela 1. Insira abaixo os resultados do experimento 1

Posição do Frasco	Número da folha mais distante que apresenta gotículas (1-7)	Contaminação por gotículas na terceira folha de papel (baixa, média, alta, muito alta)?
Frasco **PLANO**		
Inclinado para **CIMA**		
Inclinado para **BAIXO**		

Compartilhe os resultados online**:** em **Ingles** https://bit.ly/facemaskchallengedata, ou **Portugues** https://forms.gle/EJHauCWTdDbny66N8 ou escaneie o código QR abaixo

Mensagem para levar para casa #FaceMaskChallenge Experimento 1:

- Microgotas orais e nasais se projetam muito longe de nossas bocas!
- Se espirrarmos com o rosto para cima, as gotículas se espalham ainda mais!
- **Tente olhar para baixo** quando espirrar ou tossir!

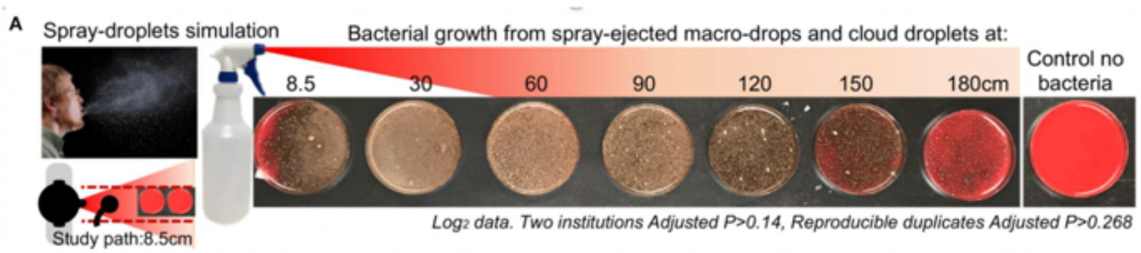

Para Professores: A figura acima e os detalhes científicos dos experimentos 1 a 3 são descritos em: https://doi.org/10.3389/fmed.2020.00260

Experimento 2. Avaliação da eficácia de uma barreira de tecido em bloquear gotículas pulverizadas

Conceito principal: Tecidos podem bloquear a propagação de gotículas líquidas pulverizadas.

Grau de Dificuldade : Fácil.

Nova Descoberta: Uma única camada de tecido doméstico (camiseta de algodão) reduz a distância de propagação de gotículas em >90%.

Objetivo de Aprendizagem: O objetivo do experimento é determinar quantas gotículas macroscópicas (visíveis) passam por uma cobertura facial e atingem uma superfície.

Duração : 30 minutos.

Materiais

- 1 frasco pulverizador.
- Líquido de cor escura (refrigerante, café frio, ou bebida esportiva) em quantidade suficiente para encher um frasco pulverizador até a metade.
- 14 folhas de papel branco ou quadriculado tamanho A4 ou carta.
- Um grande espaço vazio em uma mesa ou chão lavável.
- 1 caixa vazia de cereal.
- Pedaço de tecido/pano (pelo menos 25x25 cm).
- Tesoura
- 7-8 peças de talheres, lata de conserva, ou outros objetos a serem usados como pesos.

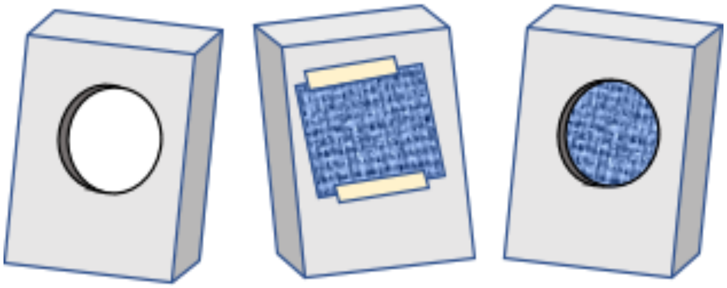

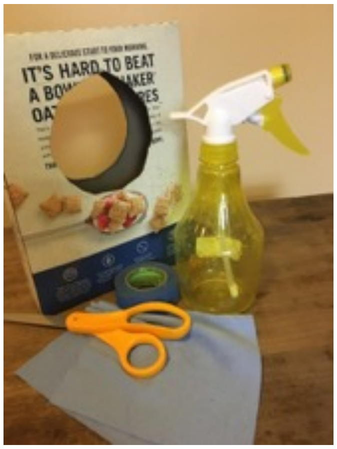

Procedimento

1. Corte duas janelas de 10x10 cm em ambos os lados de uma caixa de cereal, aproximadamente 10 cm da superfície da mesa/piso como mostrado nas imagens acima, de modo que o bocal do frasco de spray esteja no nível das janelas quando colocado no chão. Se o frasco for muito grande, coloque a caixa e as folhas de papel na borda da mesa, como mostrado na figura.
2. Coloque uma única camada de pano/tecido sobre uma das janelas com fita adesiva (ver figura). Ou posicione o tecido dentro da caixa cobrindo a janela, como mostrado na foto.
3. Apoias a caixa na posição vertical, colocando objetos pesados dentro, como talheres, lata de conserva, etc.
4. Coloque 7 folhas de papel no chão ou sobre a mesa de ponta a ponta de acordo com a figura, numerando-os de 1-7 como no **Experimento 1** (1 é mais próximo do frasco).
5. Coloque a caixa diretamente na frente do papel 1 sem espaço entre eles e coloque o frasco pulverizador plano com o bocal apontando para pulverizar através da caixa. Se o frasco for muito grande, basta incliná-lo para que o fluxo esteja o mais próximo possível da horizontal.
6. Dê uma pulverizada completa. Lembre-se de esperar 30 segundos para deixar gotículas caírem. Observe o padrão de pulverização e responda às perguntas abaixo. Guarde a caixa para usar no **Experimento 3**.

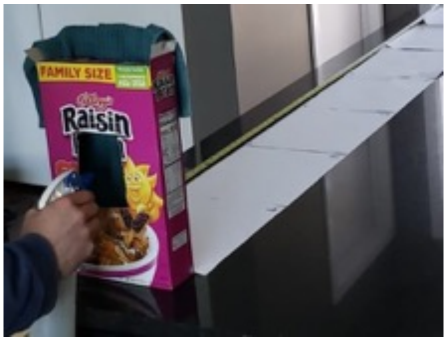

Experimento 2, continuar

Responda as Seguintes Perguntas

Q2.1 Qual era o número do papel mais distante do frasco pulverizador em que você podia ver manchas de gotículas? ___________________________

Q2.2. Como você classificaria o número de gotículas no terceiro papel? _____________________

BAIXO<br>0-10	MÉDIO<br>0-10	ALTO<br>0-10	MUITO ALTO<br>0-10

Compartilhe os resultados online**:** em **Ingles** https://bit.ly/facemaskchallengedata, ou **Portugues** https://forms.gle/EJHauCWTdDbny66N8 ou escaneie o código QR abaixo

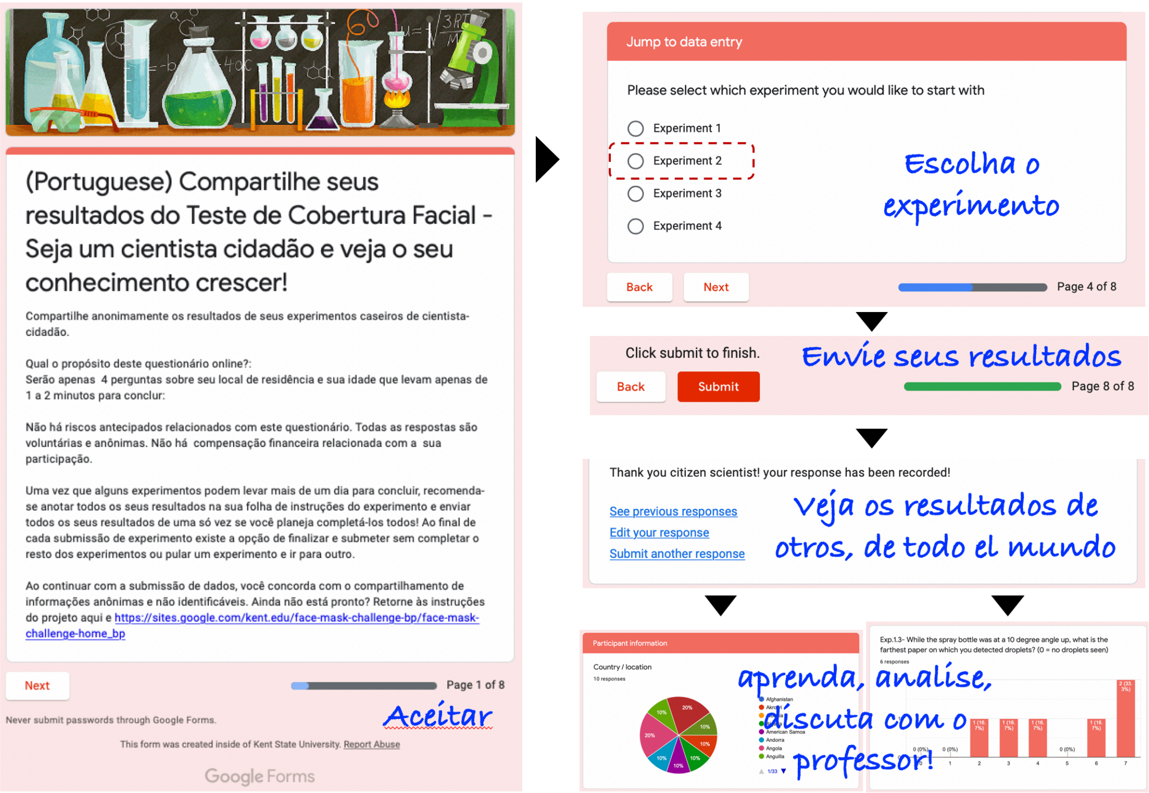

Mensagem para levar para casa #FaceMaskChallenge Experimento 2:

- O espalhamento de gotículas é reduzido por uma barreira de pano
- A maioria das gotículas são bloqueadas pela cobertura facial
- Algumas gotículas atravessam as coberturas faciais e pousam em superfícies, então... **certifique-se de lavar as mãos, usar coberturas faciais e evitar tocar objetos para evitar a propagação de doenças!**

Experimento 3. Quantas gotículas que carregam micro-organismos podem atravessar tecidos?

Conceito Principal: gotículas pulverizadas podem transportar bactérias e vírus longe da fonte.

Grau de Dificuldade: Moderado. Líquidos quentes.

Nova Descoberta: Uma máscara facial diminui o transporte de gotículas carregando bactérias e vírus em >98%.

Objetivo de aprendizagem: Usando um frasco pulverizador contendo uma "solução com micro-organismos" (iogurte ou solo diluído) e capturando os micro-organismos em placas de crescimento com gelatina, o objetivo do experimento é determinar quantas gotículas macroscópicas e microscópicas contendo micro-organismos podem atravessar uma cobertura facial (1 camada versus 2 camadas).

Duração: 48-72 horas.

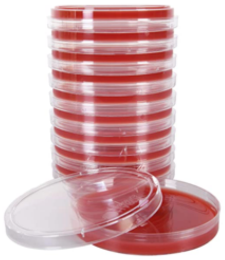

Placas de crescimento de micro-organismos
(avançado, prepare com 1 dia de antecedência)

- 1 frasco pulverizador.
- 30 ml de iogurte, solo, ou outro alimento com micro-organismos para preparar uma solução simulando micro-organismos.
- 2 caixas de cereal vazias (reutilizar caixa do Experimento 2)
- 6 Recipientes rasos de plástico de pelo menos 5 cm de diâmetro ou forminhas de cupcake de alumínio.
- 1 colher de sopa de açúcar.
- 1 cubo de caldo de caldo de carne ou similares (frango, também).
- 2 pacotes de 7 g de gelatina comum.
- 1 xícara de água.
- micro-ondas e recipiente de vidro (350 ml), ou panela com tampa para usar no fogão.
- sacos de plástico trasnparente tipo zip-lock.
- Cobertura facial limpa (deve cobrir nariz e boca) https://www.instructables.com/id/Homemade-Nutrient-Aga

Você também pode comprar placas de cultura prontas para usar em alguns vendedores *online* (p.ex. na amazon.com: Diamante Scientific Chocolate Blood Agar, Tryptic Soy Agar with 5% Lysed Sheep Blood)

Procedimento:

Parte A. No dia anterior - Prepare as placas de ágar para cresimento de micro-organismos:

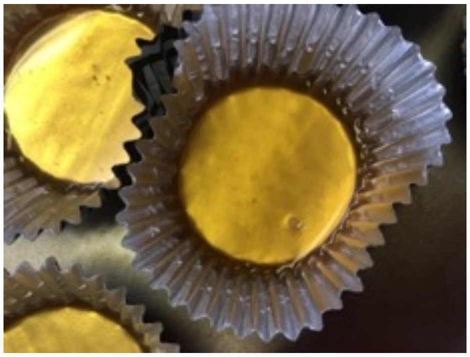

1. Misture 1 xícara de água, 1 colher de chá de açúcar e 1 cubo de caldo de carne em um recipiente de vidro no microondas por dois minutos. Mexa bem quando estiver pronto, depois aqueça por mais dois minutos. Deixe a mistura esfriar dentro do microondas por 5 minutos.
2. Alternativamente, ferva a água, mexendo o açúcar e o caldo em fogo médio-baixo por dois minutos. Cubra com uma tampa e desligue o fogo. Deixe a mistura esfriar por vários minutos.
3. **Coloque a cobertura facial no rosto e lave as mãos** para evitar contaminar suas placas!
4. Adicione lentamente os dois pacotes de gelatina em pó enquanto mexe.
5. Em seguida, despeje cuidadosamente a mistura em recipientes redondos rasos (por exemplo, tampas de recipiente de margarina recicladas) ou formas de cupcake com cerca de 1cm de profundidade. Você deve obter pelo menos 6 placas com os materiais utilizados. Coloque imediatamente as placas de gel em um recipiente coberto ou saco plástico e deixe descobertos para permitir que a umidade escape.
6. Coloque em um local fresco para solidificar durante a noite [um forno desligado funciona bem]. As placas devem estar frias antes do experimento. **NÃO TOQUE a gelatina pronta com seus dedos (isso pode contaminá-las)**.Prepare pelo menos 4 placas de crescimento de gelatina para a parte D. Você pode preparar 2 placas extras para cada tipo adicional de tecido que você deseja testar. Você pode preparar mais 2 placas para o Experimento 4 abaixo. Guarde em um saco plástico do tipo zip-lock selado até o uso.

Parte B. Prepare uma solução com micro-organismos:

1. Adicione 2 colheres de sopa de iogurte ou solo a cerca de 1/2 xícara de água morna e misture delicadamente até dissolver.
2. Coloque esta solução em um frasco pulverizador limpo rotulado com "GERMES". Sua solução contém micro-organismos (germes) inofensivos que servirão como indicadores vivos para mostrar como um protetor de tecido facial evita que gotas microscópicas se espalhem após um espirro.

Parte C. Prepare as caixas de teste:

1. Use a caixa de cereal com janelas do Experimento 2. Retire o tecido do experimento anterior e deixe reservada.
2. Prepare um recipiente coberto pronto para colocar as placas depois de pulverizadas, tal como uma caixa de cereais íntegra. Alternativamente, pode ser utilizado um saco grande de plástico do tipo Zip-lock sobre um recipiente de armazenamento de plástico limpo. Este será seu recipiente de incubação de micro-organismos.

Parte D. O Experimento (para comparar 1 camada versus 2 camadas):

1. Ao fazer o experimento, use uma COBERTURA FACIAL para que você não contamine acidentalmente as placas. **NÃO toque na gelatina com os dedos** (isso pode contaminá-las!)
2. Prepare 4 **placas de gelatina** para testar a eficácia dos protetores faciais de tecido. Marque as placas como #0 ('sem protetor), #1 ('1 camada'), #2 ('2 camadas') e #3 (o protetor facial que você tem usado). Vamos testar 2 camadas de tecido, primeiro.
3. Dobre uma peça de tecido ao meio (uma que ainda não foi usada em outro experimento). Cubra a janela da caixa com as 2 **camadas de tecido** – use fita adesiva para ajudar a mantê-la no lugar.
4. Com a placa #2 sobre uma superfície plana, coloque as janelas da caixa sobre ela. Coloque a caixa na horizontal de maneira que a janela com a cobertura de tecido esteja voltada para cima (ver Figura X).
5. A partir de cerca de 13 cm de distância do tecido um ângulo de 45 graus, pulverize DUAS VEZES na janela. Aguarde 30 segundos para que as microgotas aterrissem cuidadosamente
6. Levante cuidadosamente a caixa e coloque a placa na caixa de incubação. Retire o pano da caixa. Limpe a caixa de teste com toalha de papel embebida em álcool e descarte, mas guarde a caixa para o resto do experimento.
7. **Repetir etapas 2 – 6** usando
   a. 1 camada de tecido (placa #1).
   b. seu próprio protetor facial (placa #3).
   c. sem tampa (placa #0).
8. Feche o recipiente de incubação. Deixe-o em um local morno onde possa permanecer protegido por alguns dias (em cima da geladeira funciona bem).
9. Descarte a caixa, tecido utilizado e papel toalha cuidadosamente. Limpe a área de trabalho com álcool 70% e lave bem as mãos!
10. Verifique as placas de incubação após **24-48 horas**. **Lembre-se de usar o protetor facial limpo e lavar as mãos antes de verificar as placas.** Conte o número de manchas (**unidades formadoras de colônias, UFC**) que se formaram em cada placa. Se possível, deixe as placas no recipiente durante a observação. NÃO toque na superfície das placas! Você pode usar a mesma caixa para o **Experimento 4**.

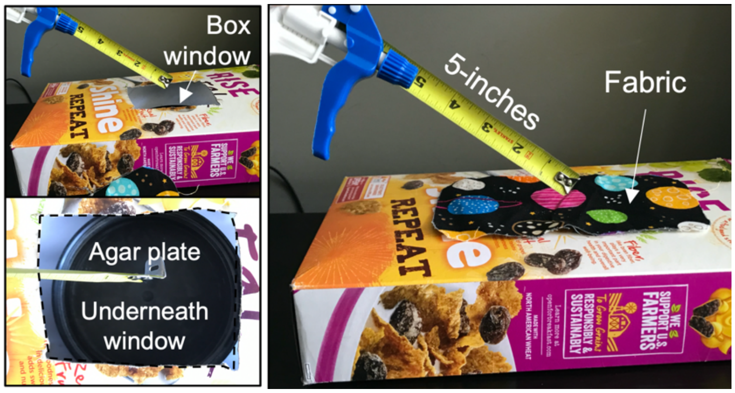

Experimento 3, cont.

Finalizar:

1. Registre seus dados na tabela abaixo.
2. Lave as mãos depois de observar as placas.
3. Repita a observação após **48 horas.** Registre a contagem do número das Unidades Formadoras de Colônias (UFC).
4. Descarte as placas de micro-organismos e a caixa de incubação no lixo, limpe a área completamente com 70% de álcool e, finalmente, lave as mãos.

Tabela 2.

	A	B	C	D	E	F	G
**Placa**	UFC em 0h	UFC em 24h	UFC em 48horas	Diâmetro da placa em cm	Raio da placa (metade da coluna D) em cm	Área da placa = pi x r^2= (3,14 x raio x raio) em cm^2	**UFC/cm^2 (coluna C dividida pela coluna F)**
**0**	0						____=Linha de Base G
**1**	0						
**2**	0						
**3**	0						

Responda as Seguintes Perguntas

Q3.1 O que você observou? ________________

Q3.2 Uma camada de tecido foi eficaz na prevenção da transferência de micro-organismos? ______________

Q3.3 Duas camadas foram iguais ou mais eficazes do que uma camadav? ________________

Q3.4 Uma ou duas camadas de tecido podem bloquear todos os micro-organismos produzidos pelo espirro? ________________

Q3.5 Seu protetor facial é melhor do que nenhuma proteção para impedir a transferência de micro-organismos? ________________

Compartilhe seus dados: insira os resultados da Coluna G nesse formulário:
em **Ingles** https://bit.ly/facemaskchallengedata, ou **Portugues** https://forms.gle/EJHauCWTdDbny66N8
Ou escaneie o código QR abaixo

Mensagem para levar para casa #FaceMaskChallenge Experimento 3:

- Uma camada de tecido ajuda a bloquear micro-organismos, mas não é a melhor opção
- Duas camadas de tecido são ainda melhores!
- Mesmo com 2 camadas de tecido nem todas as gotas são capturadas pela proteção facial, **por isso é muito importante lavar as mãos e praticar uma boa higiene!**
- **Uma vez que os micro-organismos em microgotículas estão presos na sua proteção facial é importante que você lave regularmente a cobertura facial!**

Experimento 4
Quantos micro-organismos saem da minha boca enquanto falo?

Conceito Principal: Todos nós produzimos gotículas com micro-organismos quando falamos.

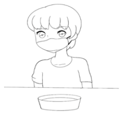

Grau de Dificuldade: Moderado.

Nova Descoberta: Uma cobertura facial impede que nossas gotículas de saliva contaminem o ambiente.

Objetivo de aprendizagem: Ao falar com ou sem máscara facial e capturar os micro-organismos em placas de crescimento de gelatina, o objetivo do experimento é determinar como efetivamente as barreiras de tecido impedem a propagação de gotículas orais.

Duração: 48-72 horas.

Materiais de placas de incubação
(avançado, prepare-se com antecedência)

- 2 Placas de incubação de micro-organismos (veja Experimento 3).
- 1 caixa de cereal vazia para incubar placas de crescimento de micro-organismos.
- Máscara facial de tecido ou cobertura facial com duas camadas têxteis que cubram tanto nariz quanto a boca).
- Ou pedaço de tecido preferido.

Procedimento:

Parte A. No dia anterior - Prepare as placas de ágar de crescimento de micro-organismos **- ver Experimento 3 Parte A**

Parte B. O Experimento (para determinar quantas gotículas nós produzimos ao falar em tom elevado):

1. Baixe o aplicativo gratuito CDC Noise APP no seu smartphone. Abra o aplicativo e ajuste o volume da sua fala para 70 decibéis.
2. Rotular 2 placas de gelatina como "#falalivre" e "#falacoberta ”.
3. Prepare um recipiente coberto pronto para colocar as placas depois de pulverizadas, como descrito no experimento 3. Uma caixa de cereal sem cortes é ideal. Alternativamente, você por usar um saco plástico grande do tipo Zip-locksobre um recipiente de armazenamento de plástico limpo pode. Este será seu recipiente de incubação.
4. Sente-se em uma mesa e coloque uma placa de gelatina rotulada como "#falalivre" na mesa a cerca de 20 cm de distância da sua boca.
5. Tome um gole de água e leia o seguinte texto lentamente e claramente como se estivesse falando com alguém do outro lado da sala (70 dB ao comprimento do seu braço).

"Sou um cientista cidadão. Vou ler isso em voz alta como um experimento para testar a importância das proteções faciais. Eu quero ver quantas gotículas de saliva eu produzo que contaminam o ambiente quando eu conto de 1 a 100. [... contar em voz alta até 100]. Obrigado e boa saúde!”

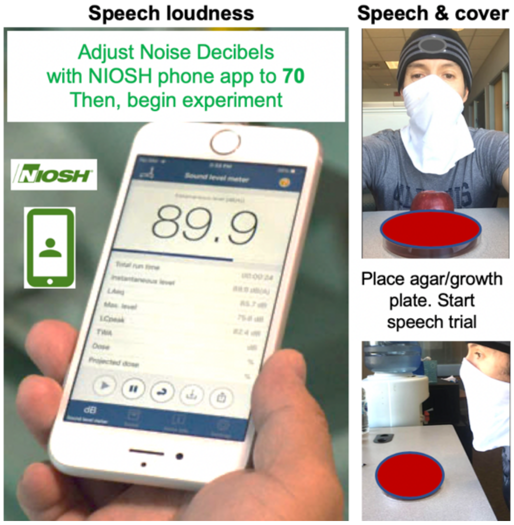

6. Transfira cuidadosamente a placa #falalivre para o recipiente de incubação.
7. **Cubra seu rosto e nariz com uma dupla camada de tecido**.
8. Sente-se em uma mesa e coloque uma placa marcada como "#falacoberta" na mesa a cerca de 20 cm de distância da sua boca.
9. **Repita** a fala no passo 5 e transfira cuidadosamente a placa #5 falacoberta para o recipiente de incubação.
10. Feche o recipiente de incubação. Deixe-o em um local morno onde possa permanecer protegido sendo observado por 2 dias (em cima da geladeira funciona bem).
11. Verifique as placas de incubação após 24 horas. **Lembre-se de usar o protetor facial limpo e lavar as mãos antes de verificar as placas.** Conte o número de manchas (unidades formadoras de colônias, UFC) que se formaram em cada placa. Se possível, deixe as placas no recipiente durante a observação. NÃO toque na superfície das placas!

Experimento 4, cont.

Finilizar:

1. Registre seus dados na tabela abaixo.
2. Lave as mãos depois de observar as placas.
3. Repita a observação após 48 horas. Registre a contagem das UFC.
4. Descarte as placas de germe e a caixa de incubação no lixo, limpe a área completamente com álcool 70% e, finalmente, lave as mãos.
5. Compartilhe seus dados: insira os resultados da Coluna G no seguinte formulário : https://bit.ly/facemaskchallengedata

Table 3.

	A	B	C	D	E	F	G
**Placa**	UFC em 0h	UFC em 24h	UFC em 48horas	Diâmetro da placa em cm	Raio da placa (metade da coluna D) em cm	Área da placa = pi x r^2= (3,14 x raio x raio) em cm^2	**CFU/cm^2 (coluna C dividida pela coluna F)**
**4**	0						____=Linha de Base G
**5**	0						

Responda as Seguintes Perguntas de Tabela 3

Q4.1 *O que você observou?* ____________________

Q4.2 *Um protetor facial de duas camadas foi eficaz na prevenção da propagação de micro-organismos?* ____

Q4.3 *O tecido conteve todos os micro-organismos?* _____________________

Q4.4 *Que outro tipo de higiene é necessário para impedir a propagação efetiva de micro-organismos ?* ___________________________________

Compartilhe seus dados: insira os resultados da Coluna G nesse formulário:
em **Ingles** https://bit.ly/facemaskchallengedata, ou **Portugues** https://forms.gle/EJHauCWTdDbny66N8
Ou escaneie o código QR abaixo

Mensagem para levar para casa **#FaceMaskChallenge Experimento 4**:

- Nós espalhamos muitos micro-organismos invisíveis simplesmente falando – tenha isso em mente quando estiver praticando o distanciamento social.
- Uma parte da solução para COVID-19 ou outras infecções espalhadas por gotícula é usar um protetor facial e realizar lavagem cuidadosa das mãos. Espalhe a notícia! Para imprimir e postar sinais de aviso https://figshare.com/articles/Door_Signs_to_Promote_Public_Droplet_Safety_Amidst_COVID-19/12202808/1

Para professores: O aplicativo de smartphone mostrado na figura da página 10 e os detalhes científicos do Experimento 4 são descritos em: https://doi.org/10.3389/fmed.2020.00504

Glossário

Vírus: Uma partícula tão pequena quanto 20 nanômetros que pode entrar e infectar células. Ao entrar, os vírus se multiplicam criando milhares de novas partículas que explodem as células e então escapam em gotículas líquidas para infectar as pessoas
Gotícula: Uma gota muito pequena de líquido
Aerossol: Uma substância armazenada sob pressão que é então liberada como uma nuvem fina
Epidemia: Ocorrência generalizada de uma doença infecciosa emu ma comunidade em um determinado período
Pandemia: Prevalente em todo um país ou todo o mundo
Prevenção: Ação de fazer algo parar de acontecer ou surgir
Máscaras médicas: Tipo de equipamento de proteção individual feita para profissionais de saúde que reduzem o espalhamento de infecções respiratórias
Cobertura facial: qualquer pedaço de tecido colocado sobre a boca e a face para prevenir o espalhamento de infecção respiratória

Links

Face Mask Challenge Citizen Science Module: https://sites.google.com/kent.edu/face-mask-challenge/face-mask-challenge-home
Encyclopedia Britannica COVID-19: https://www.britannica.com/explore/savingearth/covid-19/
The Scale of Things: https://www.nano.gov/nanotech-101/what/nano-size
Research on face covers and germ transmission: https://doi.org/10.3389/fmed.2020.00260
Face Mask Challenge Citizen Science Data sharing form https://forms.gle/EJHauCWTdDbny66N8
Printable Door sign reminders to encourage wearing face masks
https://figshare.com/articles/Door_Signs_to_Promote_Public_Droplet_Safety_Amidst_COVID-19/12202808/1
Versões em quatro linguagens adicionais fazem parte da submissão inicial e podem ser acessadas em https://sites.google.com/kent.edu/face-mask-challenge/face-mask-challenge-home

---

Authors: Sarah E. Eichler[1], Austin P. Hopperton[2], Juan Jose Alava[3], Antonio Jr. Pereira[4], Rukhsana Ahmed[5], Zisis Kozlakidis[6], Sanja Ilic[7], Alex Rodriguez-Palacios[2,8]

Cartoonist: Maddie Rosemark

Affiliations: [1]Department of Biological Sciences, Kent State University, USA; [2]Division of Gastroenterology and Liver Disease, Case Western Reserve University School of Medicine, USA; [3]Institute for the Oceans and Fisheries, The University of British Columbia, Canada; [4]Institute of Technology, Federal University of Pará, Brazil; [5] Department of Communication, University at Albany, SUNY, USA; [6]International Agency for Research on Cancer, World Health Organization, Lyon, France; [7]Department of Human Sciences Human Nutrition, The Ohio State University, USA; [8]University Hospitals Research and Education Institute, University Hospitals Cleveland Medical Center, Cleveland, OH, USA.

Translations: Juan Jose Alava, Diana M. Rodriguez, Diego A. Pulido, Zisis Kozlakidis, Antonio Jr. Pereira, Alex Rodriguez-P & Maissa Zeghidi.

Open CC BY license http://creativecommons.org/licenses/by/4.0/

Quatro versões em idiomas adicionais (espanhol, inglês, francês, português) fazem parte da apresentação inicial e estão vinculadas por https://sites.google.com/kent.edu/face-mask-challenge/face-mask-challenge-home

Arquivo - Versões atualizadas do módulo e contato com os autores com sugestões / dúvidas. Um repositório foi criado para o Arquivo das últimas versões do módulo aprovadas pelos autores após a publicação e para contribuições feitas pela comunidade em: **https://github.com/axr503/education**. Alex Rodriguez-Palacios, Assistant Professor, School of Medicine, axr503@case.edu.

International collaboration of scientists & educators from

SCHOOL OF MEDICINE CASE WESTERN RESERVE UNIVERSITY · University Hospitals · Kent State University · UNIVERSIDADE FEDERAL DO PARÁ · THE OHIO STATE UNIVERSITY COLLEGE OF EDUCATION AND HUMAN ECOLOGY · LMIC BCNET · International Agency for Research on Cancer/WHO · UNIVERSITY AT ALBANY State University of New York · UBC THE UNIVERSITY OF BRITISH COLUMBIA

Module version 1 (August 20/2020)

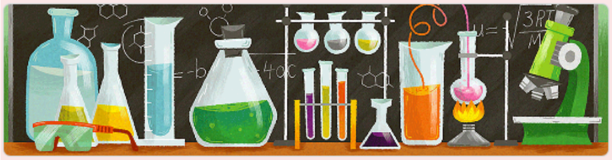

(Portuguese) Compartilhe seus resultados do Teste de Cobertura Facial - Seja um cientista cidadão e veja o seu conhecimento crescer!

Compartilhe anonimamente os resultados de seus experimentos caseiros de cientista-cidadão.

Qual o propósito deste questionário online?:
Serão apenas 4 perguntas sobre seu local de residência e sua idade que levam apenas de 1 a 2 minutos para conclur:

Não há riscos antecipados relacionados com este questionário. Todas as respostas são voluntárias e anônimas. Não há compensação financeira relacionada com a sua participação.

Uma vez que alguns experimentos podem levar mais de um dia para concluir, recomenda-se anotar todos os seus resultados na sua folha de instruções do experimento e enviar todos os seus resultados de uma só vez se você planeja completá-los todos! Ao final de cada submissão de experimento existe a opção de finalizar e submeter sem completar o resto dos experimentos ou pular um experimento e ir para outro.

Ao continuar com a submissão de dados, você concorda com o compartilhamento de informações anônimas e não identificáveis. Ainda não está pronto? Retorne às instruções do projeto aqui e https://sites.google.com/kent.edu/face-mask-challenge-bp/face-mask-challenge-home_bp

Next Page 1 of 8

Informação Pessoal

#FaceCoverChallenge

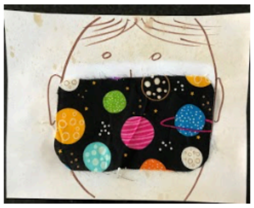

Ao clicar na opção <<SIM, Eu Concordo>>, você indica que você leu o texto acima, concorda em participar voluntariamente e que você tem 18 anos ou mais de idade. Se você não quiser participar, clique na opção <<NÃO, eu não quero participar da submissão de dados>> ou simplesmente feche esta janela. Se você concordar em participar, você será direcionado automaticamente para o formulário de submissão de dados. *

- (●) Sim, eu concordo em participar e compartilhar meus resultados. (Clique em SEGUIR para continuar)
- ( ) Não, eu não quero participar (Clique SEGUIR para continuar)

É a sua primeira submissão de dados no Ciência Cidadã: desafio do teste da máscara facial? *

sim ▾

Back Next Page 2 of 8

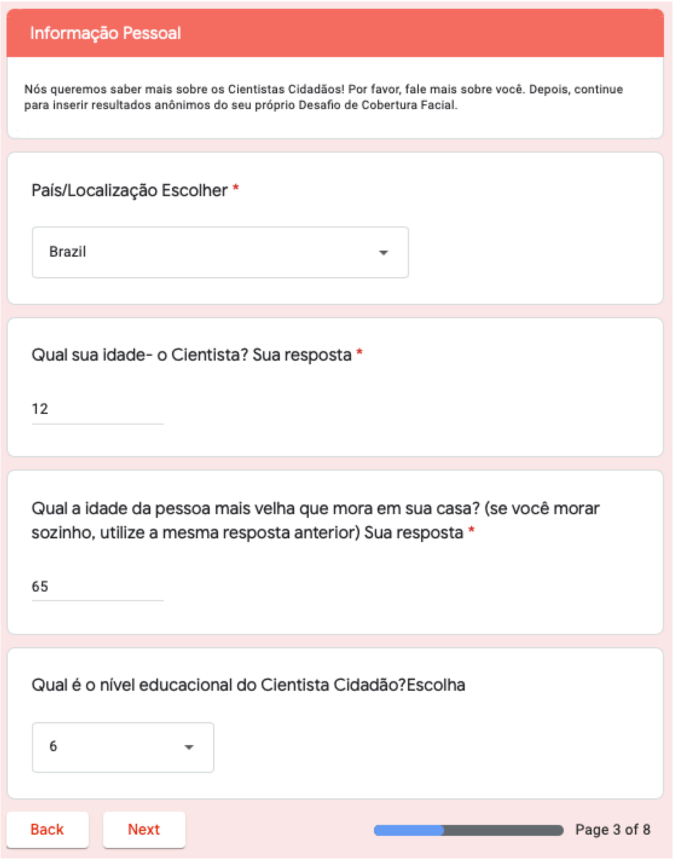

Informação Pessoal

Nós queremos saber mais sobre os Cientistas Cidadãos! Por favor, fale mais sobre você. Depois, continue para inserir resultados anônimos do seu próprio Desafio de Cobertura Facial.

País/Localização Escolher *

Brazil

Qual sua idade- o Cientista? Sua resposta *

12

Qual a idade da pessoa mais velha que mora em sua casa? (se você morar sozinho, utilize a mesma resposta anterior) Sua resposta *

65

Qual é o nível educacional do Cientista Cidadão?Escolha

6

Back Next Page 3 of 8

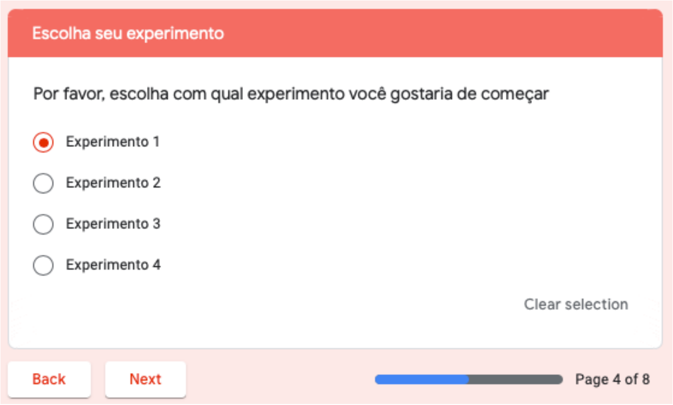

Escolha seu experimento

Por favor, escolha com qual experimento você gostaria de começar

- Experimento 1
- Experimento 2
- Experimento 3
- Experimento 4

Clear selection

Back Next Page 4 of 8

Experimento 1 - Até onde as gotículas viajam?

Exp.1.1 SEM PROTEÇÃO TÊXTIL: Quando o borrifador estava plano, qual o número do papel mais distante no qual você detectou gotículas? (0 = Sem gotículas) [While the spray bottle was flat what is the farthest paper on which you detected droplets? (0 = no droplets seen)]

	0	1	2	3	4	5	6	7	
Mais próximo do borrifador [Closest to Spray Bottle]	○	○	○	○	○	○	○	○	mais distante do borrifador [Furthest from Spray Bottle]

Exp.1-Q2/7. - SEM PROTEÇÃO TÊXTIL: Quando o borrifador estava plano, como você descreveria a quantidade de gotículas contaminando o papel número 3?

- ○ Baixa (0-10)
- ○ Média (11-100)
- ○ Alta (100-500)
- ○ Muito Alta (500+)

Exp.1-Q3/7. - SEM PROTEÇÃO TÊXTIL: Quando o borrifador estava em um ângulo de 10 graus para cima, qual foi o número do papel mais distante no qual você detectou gotículas? (0- Sem gotículas)

	0	1	2	3	4	5	6	7	
Mais próximo do borrifador	○	○	○	○	○	○	○	○	Mais distante do borrifador

Exp.1-Q4/7. - SEM PROTEÇÃO TÊXTIL: Quando o borrifador estava em um ângulo de 10 graus para cima, como você descreveria a quantidade de gotículas contaminando o papel número 3?

- ○ Baxia (0-10)
- ○ Média (11-100)
- ○ Alta (100-500)
- ○ Muito Alta (500+)

Exp.1-Q5/7. - SEM PROTEÇÃO TÊXTIL: Quando o borrifador estava em um ângulo de 10 graus para baixo, qual foi o número do papel mais distante no qual você detectou gotículas? (0- Sem gotículas)

	0	1	2	3	4	5	6	7	
Mais próximo do borrifador	○	○	○	○	○	○	○	○	mais distante do borrifador

Exp.1-Q6/7. - SEM PROTEÇÃO TÊXTIL: Quando o borrifador estava em um ângulo de 10 graus para baixo, como você descreveria a quantidade de gotículas contaminando o papel número 3?

- ○ Baixa (0-10)
- ○ Média (11-100)
- ○ Alta (100-500)
- ○ Muito Alta (500+)

Exp.1-Q7/7 - Que posição do borrifador pulverizou mais longe?

- ○ Frasco plano
- ○ Frasco inclinado 10 graus para cima
- ○ Frasco inclinado 10 graus para baixo
- ○ Outro

Você gostaria de realizar outro experimento ou finalizar e submeter seus dados?

- ◉ Experimento 2
- ○ Experimento 3
- ○ Experimento 4
- ○ Finalizar e Submeter Dados

Clear selection

Back   Next   Page 5 of 8

Experimento 2 - Quão eficaz é uma barreira de tecido protetor contra gotículas?

Exp.2-Q1/3. Que tipo de tecido/barreira têxtil você usou? (Você pode escolher várias opções.)

- ☐ Algodão
- ☐ Poliéster
- ☐ Algodão/poliéster
- ☐ Seda
- ☐ Lã
- ☐ Toalha
- ☐ Lycra
- ☐ Camiseta
- ☐ Cachecol
- ☐ Outro

Exp.2-Q2/3. – COM BARREIRA TÊXTIL: qual foi o número do papel mais distante do borrifador no qual você detectou gotículas? (0- Sem gotículas)

	0	1	2	3	4	5	6	7	
Mais próximo do frasco	○	○	○	○	○	○	○	○	Mais distante do frasco

Exp.2-Q3/3. – COM BARREIRA TÊXTIL: Como você descreveria a quantidade de gotículas contaminando o papel número 3?

- ○ Baixa (0-10)
- ○ Média (11-100)
- ○ Alta (100-500)
- ○ Muito Alta (500+)

Você gostaria de realizar outro experimento ou finalizar e submeter seus dados?

- ○ Experimento 1
- ◉ Experimento 3
- ○ Experimento 4
- ○ Finalizar e Submeter Dados

Clear selection

Back Next Page 6 of 8

Experimento 3 – Qual a quantidade de gotículas transportando germes que podem passar por duas camadas de tecido?

Exp.3-Q1/3.- Placa # 0 Sem tecido/cobertura têxtil (coluna G: colônias/cm2) Sua resposta abaixo

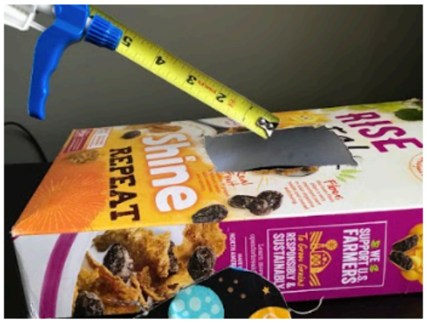

Your answer

Exp.3-Q2/3.- Placa # 1 com uma camada de tecido/cobertura têxtil (coluna G: colônias/cm2) Sua resposta abaxio

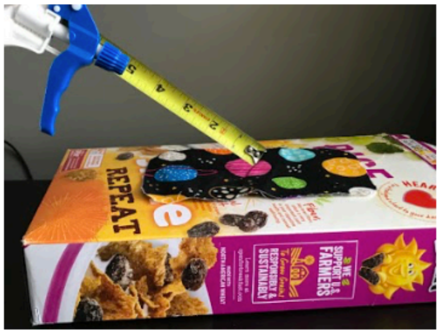

Your answer

Exp.3-Q3/3.- Placa # 2 com duas camadas de tecido/cobertura têxtil (coluna G: colônias/cm2) Sua resposta abaxio

Your answer

Você gostaria de realizar outro experimento ou finalizar e submeter seus dados?

- ◯ Experimento 1
- ◯ Experimento 2
- ◉ Experimento 4
- ◯ Finalizar e Submeter Dados

Clear selection

Back Next Page 7 of 8

Experimento 4 – Quantos germes saem da minha boca quando eu falo?

Exp.4-Q1/2.- SEM COBERTURA FACIAL: placa de fala descoberta (coluna G: colônias/cm2) Sua resposta abaxio

Your answer

Exp.4-Q2/2.- COM COBERTURA FACIAL: placa de fala coberta (coluna G: colônias/cm2) Sua resposta abaxio

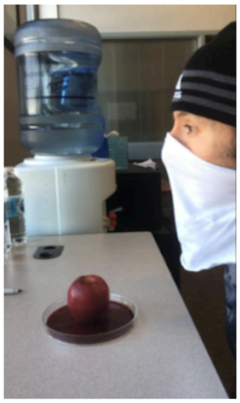

Your answer

Você gostaria de realizar outro experimento ou finalizar e submeter seus dados?

- ○ Experimento 1
- ○ Experimento 2
- ○ Experimento 3
- ◉ Finalizar e Submeter Dados

Clear selection

Back Next Page 8 of 8

(Portuguese) Compartilhe seus resultados do Teste de Cobertura Facial - Seja um cientista cidadão e veja o seu conhecimento crescer!

Click submit to finish.

Back Submit Page 8 of 8

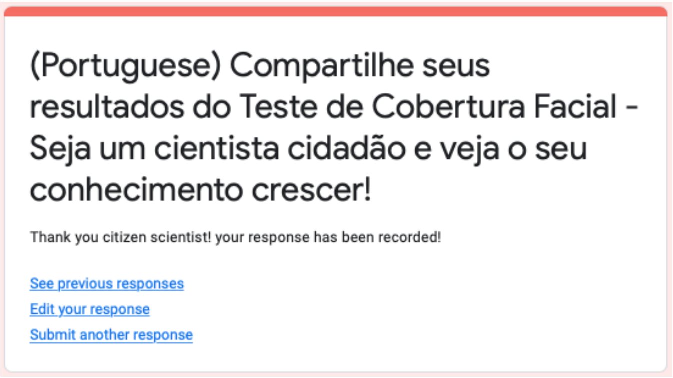

Compartilhe os RESULTADOS

(abaixo está uma série de simulação ou respostas aleatórias para ilustração)
Insira apenas resultados reais para que todos possamos ver as descobertas reais.
Se alguns resultados não forem semelhantes aos seus dados, discuta com um professor.
E pergunte / leia os resultados reais de experimentos de laboratório que foram obtidos em um Centro de Pesquisa Médica.
Os estudos originais são publicados aqui:

https://doi.org/10.3389/fmed.2020.00260 https://doi.org/10.3389/fmed.2020.00504

Textile Masks and Surface Covers—A Spray Simulation Method and a "Universal Droplet Reduction Model" Against Respiratory Pandemics. Rodriguez-Palacios A, Cominelli F, Basson AR, Pizarro TT, and Ilic S. (2020) Front. Med. 7:260. doi: 10.3389/fmed.2020.00260

Germ-Free Mice Under Two-Layer Textiles are Fully Protected From Bacteria in Sprayed Microdroplets: A Functional in-vivo Test Strategy of Facemasks and Filtration Materials. Rodriguez-Palacios A, Conger M, and Cominelli F. (2020) Front. Med. 7:504. doi: 10.3389/fmed.2020.00504

A Citizen Science Facemask Experiment and Educational Modules to Increase Coronavirus Safety in Communities and Schools. Eichler SE, Hopperton AP, Alava JJ, Pereira A, Ahmed R, Kozlakidis Z, Ilic S and Rodriguez-Palacios A (2020) Front. Med. 7:486. doi: 10.3389/fmed.2020.00486
https://doi.org/10.3389/fmed.2020.00486

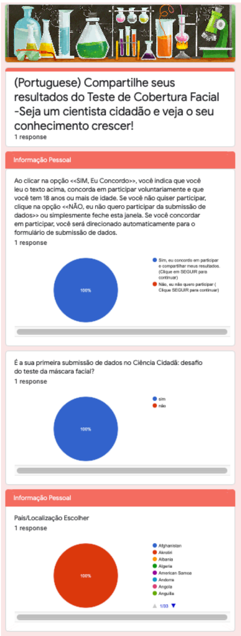

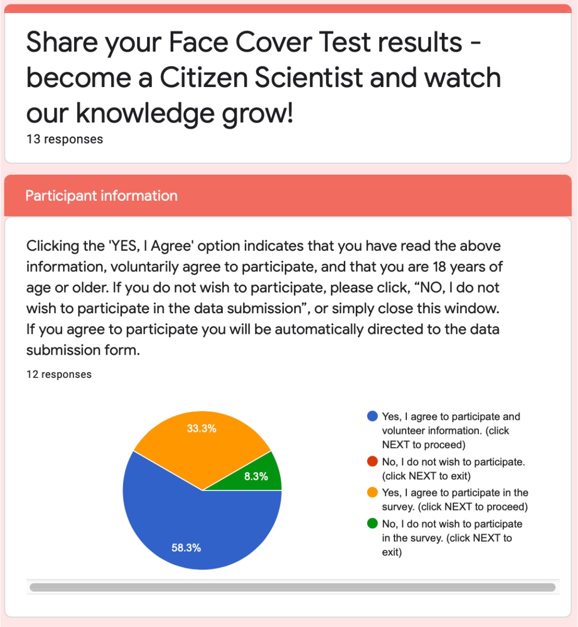

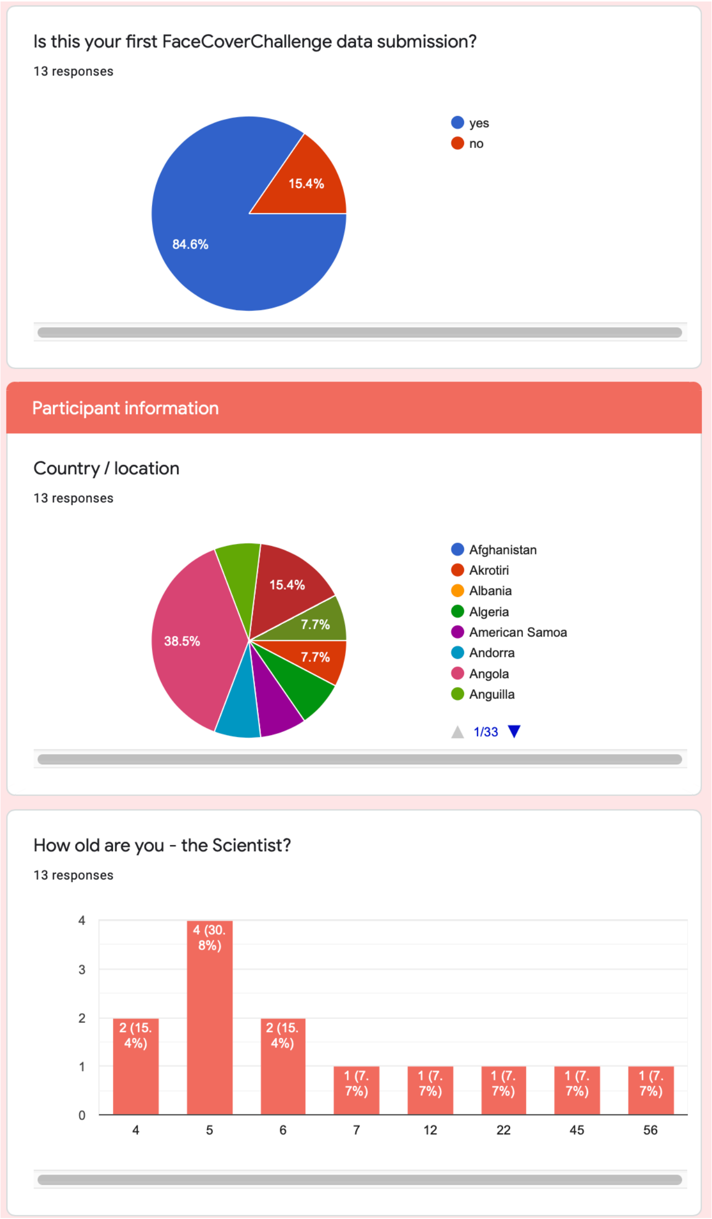
Is this your first FaceCoverChallenge data submission?
13 responses
yes
no
15.4%
84.6%
Participant information
Country / location
13 responses
Afghanistan
Akrotiri
Albania
Algeria
American Samoa
Andorra
Angola
Anguilla
1/33
15.4%
7.7%
7.7%
38.5%
How old are you - the Scientist?
13 responses
4 (30.8%)
2 (15.4%)
2 (15.4%)
1 (7.7%)
1 (7.7%)
1 (7.7%)
1 (7.7%)
1 (7.7%)
0
1
2
3
4
4
5
6
7
12
22
45
56

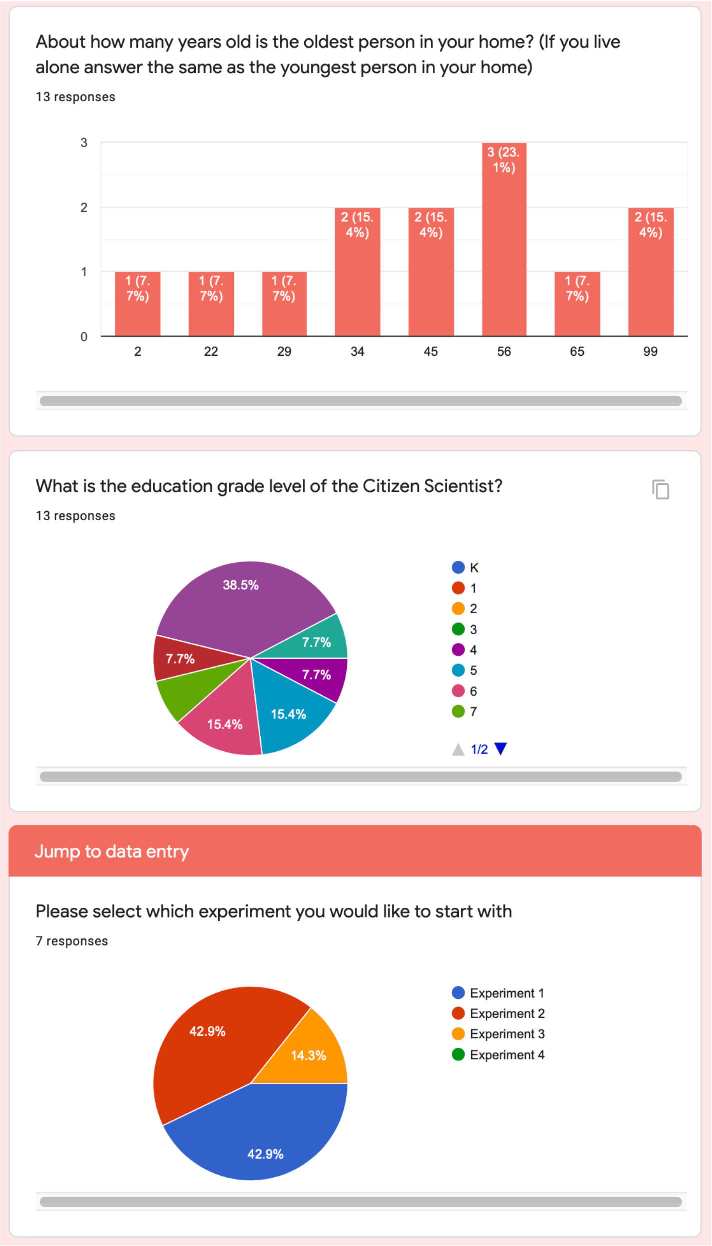

Veja os resultados restantes no link do formulário de resposta
https://docs.google.com/forms/d/e/1FAIpQLSd9cV7HQzxr49MsC-icHCzxIOnlhX2z7e7iza3cJ-NGzJaFRw/viewform

A Citizen Science Facemask Experiment and Educational Modules to Increase Coronavirus Safety in Communities and Schools.
Eichler SE, Hopperton AP, Alava JJ, Pereira A, Ahmed R, Kozlakidis Z, Ilic S and Rodriguez-Palacios A (2020) Front. Med. 7:486.
doi: 10.3389/fmed.2020.00486
SUPPLEMENTARY MODULE - In English.
Provided by Alex Rodriguez-P. axr503@case.edu.
V1 (August 20, 2020) with the accepted publication.
Results submission form in English:
https://docs.google.com/forms/d/e/1FAIpQLSd9cV7HQzxr49MsC-icHCzxIOnlhX2z7e7iza3cJ-NGzJaFRw/viewform

DATA SUBMISSION FORMS in:
English: https://bit.ly/facemaskchallengedata
Français: https://forms.gle/5q7V44nbT8fu6Jvz6
Espanol: https://forms.gle/iWgE8JFeDvezxXTq6
Portugues: https://forms.gle/EJHauCWTdDbny66N8

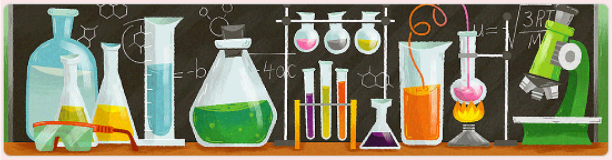

(Portuguese) Compartilhe seus resultados do Teste de Cobertura Facial - Seja um cientista cidadão e veja o seu conhecimento crescer!

Compartilhe anonimamente os resultados de seus experimentos caseiros de cientista-cidadão.

Qual o propósito deste questionário online?:
Serão apenas 4 perguntas sobre seu local de residência e sua idade que levam apenas de 1 a 2 minutos para conclur:

Não há riscos antecipados relacionados com este questionário. Todas as respostas são voluntárias e anônimas. Não há compensação financeira relacionada com a sua participação.

Uma vez que alguns experimentos podem levar mais de um dia para concluir, recomenda-se anotar todos os seus resultados na sua folha de instruções do experimento e enviar todos os seus resultados de uma só vez se você planeja completá-los todos! Ao final de cada submissão de experimento existe a opção de finalizar e submeter sem completar o resto dos experimentos ou pular um experimento e ir para outro.

Ao continuar com a submissão de dados, você concorda com o compartilhamento de informações anônimas e não identificáveis. Ainda não está pronto? Retorne às instruções do projeto aqui e https://sites.google.com/kent.edu/face-mask-challenge-bp/face-mask-challenge-home_bp

Next

Page 1 of 8

Informação Pessoal

#FaceCoverChallenge

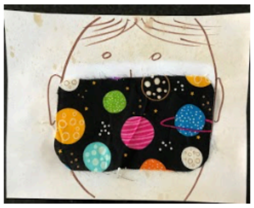

Ao clicar na opção <<SIM, Eu Concordo>>, você indica que você leu o texto acima, concorda em participar voluntariamente e que você tem 18 anos ou mais de idade. Se você não quiser participar, clique na opção <<NÃO, eu não quero participar da submissão de dados>> ou simplesmente feche esta janela. Se você concordar em participar, você será direcionado automaticamente para o formulário de submissão de dados. *

- (●) Sim, eu concordo em participar e compartilhar meus resultados. (Clique em SEGUIR para continuar)
- ( ) Não, eu não quero participar (Clique SEGUIR para continuar)

É a sua primeira submissão de dados no Ciência Cidadã: desafio do teste da máscara facial? *

sim

Back Next

Page 2 of 8

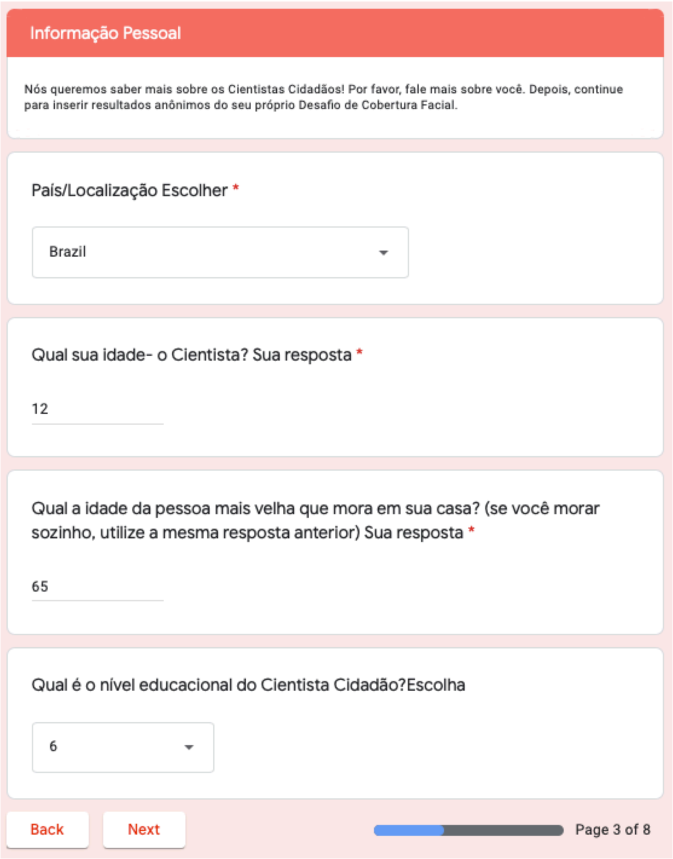
Informação Pessoal

Nós queremos saber mais sobre os Cientistas Cidadãos! Por favor, fale mais sobre você. Depois, continue para inserir resultados anônimos do seu próprio Desafio de Cobertura Facial.

País/Localização Escolher *

Brazil

Qual sua idade- o Cientista? Sua resposta *

12

Qual a idade da pessoa mais velha que mora em sua casa? (se você morar sozinho, utilize a mesma resposta anterior) Sua resposta *

65

Qual é o nível educacional do Cientista Cidadão?Escolha

6

Back Next Page 3 of 8

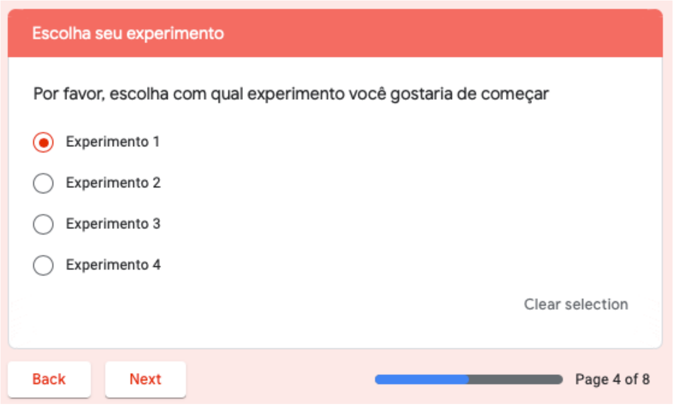
Escolha seu experimento

Por favor, escolha com qual experimento você gostaria de começar

- Experimento 1
- Experimento 2
- Experimento 3
- Experimento 4

Clear selection

Back Next Page 4 of 8

Experimento 1 - Até onde as gotículas viajam?

Exp.1.1 SEM PROTEÇÃO TÊXTIL: Quando o borrifador estava plano, qual o número do papel mais distante no qual você detectou gotículas? (0 = Sem gotículas) [While the spray bottle was flat what is the farthest paper on which you detected droplets? (0 = no droplets seen)]

	0	1	2	3	4	5	6	7	
Mais próximo do borrifador [Closest to Spray Bottle]	○	○	○	○	○	○	○	○	mais distante do borrifador [Furthest from Spray Bottle]

Exp.1-Q2/7. - SEM PROTEÇÃO TÊXTIL: Quando o borrifador estava plano, como você descreveria a quantidade de gotículas contaminando o papel número 3?

- ○ Baixa (0-10)
- ○ Média (11-100)
- ○ Alta (100-500)
- ○ Muito Alta (500+)

Exp.1-Q3/7. - SEM PROTEÇÃO TÊXTIL: Quando o borrifador estava em um ângulo de 10 graus para cima, qual foi o número do papel mais distante no qual você detectou gotículas? (0- Sem gotículas)

	0	1	2	3	4	5	6	7	
Mais próximo do borrifador	○	○	○	○	○	○	○	○	Mais distante do borrifador

Exp.1-Q4/7. - SEM PROTEÇÃO TÊXTIL: Quando o borrifador estava em um ângulo de 10 graus para cima, como você descreveria a quantidade de gotículas contaminando o papel número 3?

- ○ Baxia (0-10)
- ○ Média (11-100)
- ○ Alta (100-500)
- ○ Muito Alta (500+)

Exp.1-Q5/7. - SEM PROTEÇÃO TÊXTIL: Quando o borrifador estava em um ângulo de 10 graus para baixo, qual foi o número do papel mais distante no qual você detectou gotículas? (0- Sem gotículas)

	0	1	2	3	4	5	6	7	
Mais próximo do borrifador	◯	◯	◯	◯	◯	◯	◯	◯	mais distante do borrifador

Exp.1-Q6/7. - SEM PROTEÇÃO TÊXTIL: Quando o borrifador estava em um ângulo de 10 graus para baixo, como você descreveria a quantidade de gotículas contaminando o papel número 3?

- ◯ Baixa (0-10)
- ◯ Média (11-100)
- ◯ Alta (100-500)
- ◯ Muito Alta (500+)

Exp.1-Q7/7 - Que posição do borrifador pulverizou mais longe?

- ◯ Frasco plano
- ◯ Frasco inclinado 10 graus para cima
- ◯ Frasco inclinado 10 graus para baixo
- ◯ Outro

Você gostaria de realizar outro experimento ou finalizar e submeter seus dados?

- ◉ Experimento 2
- ◯ Experimento 3
- ◯ Experimento 4
- ◯ Finalizar e Submeter Dados

Clear selection

Back Next Page 5 of 8

Experimento 2 - Quão eficaz é uma barreira de tecido protetor contra gotículas?

Exp.2-Q1/3. Que tipo de tecido/barreira têxtil você usou? (Você pode escolher várias opções.)

- [ ] Algodão
- [ ] Poliéster
- [ ] Algodão/poliéster
- [ ] Seda
- [ ] Lã
- [ ] Toalha
- [ ] Lycra
- [ ] Camiseta
- [ ] Cachecol
- [ ] Outro

Exp.2-Q2/3. – COM BARREIRA TÊXTIL: qual foi o número do papel mais distante do borrifador no qual você detectou gotículas? (0- Sem gotículas)

	0	1	2	3	4	5	6	7	
Mais próximo do frasco	○	○	○	○	○	○	○	○	Mais distante do frasco

Exp.2-Q3/3. – COM BARREIRA TÊXTIL: Como você descreveria a quantidade de gotículas contaminando o papel número 3?

- ○ Baixa (0-10)
- ○ Média (11-100)
- ○ Alta (100-500)
- ○ Muito Alta (500+)

Você gostaria de realizar outro experimento ou finalizar e submeter seus dados?

- ○ Experimento 1
- ◉ Experimento 3
- ○ Experimento 4
- ○ Finalizar e Submeter Dados

Clear selection

Back Next Page 6 of 8

Experimento 3 – Qual a quantidade de gotículas transportando germes que podem passar por duas camadas de tecido?

Exp.3-Q1/3.- Placa # 0 Sem tecido/cobertura têxtil (coluna G: colônias/cm2) Sua resposta abaixo

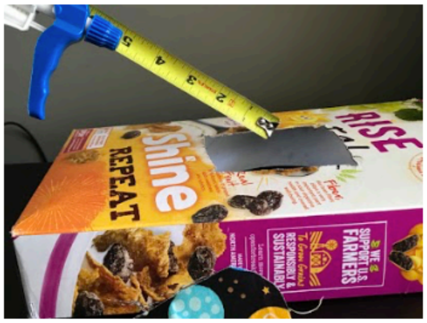

Your answer

Exp.3-Q2/3.- Placa # 1 com uma camada de tecido/cobertura têxtil (coluna G: colônias/cm2) Sua resposta abaxio

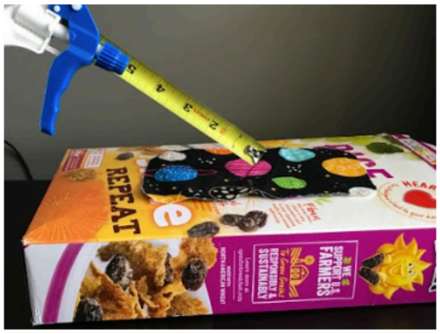

Your answer

Exp.3-Q3/3.- Placa # 2 com duas camadas de tecido/cobertura têxtil (coluna G: colônias/cm2) Sua resposta abaxio

Your answer

Você gostaria de realizar outro experimento ou finalizar e submeter seus dados?

- ○ Experimento 1
- ○ Experimento 2
- ◉ Experimento 4
- ○ Finalizar e Submeter Dados

Clear selection

Back Next Page 7 of 8

Experimento 4 – Quantos germes saem da minha boca quando eu falo?

Exp.4-Q1/2.- SEM COBERTURA FACIAL: placa de fala descoberta (coluna G: colônias/cm2) Sua resposta abaxio

Your answer

Exp.4-Q2/2.- COM COBERTURA FACIAL: placa de fala coberta (coluna G: colônias/cm2) Sua resposta abaxio

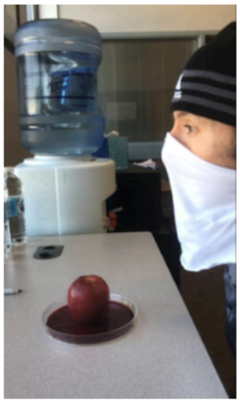

Your answer

Você gostaria de realizar outro experimento ou finalizar e submeter seus dados?

- ○ Experimento 1
- ○ Experimento 2
- ○ Experimento 3
- ◉ Finalizar e Submeter Dados

Clear selection

Back | Next | Page 8 of 8

(Portuguese) Compartilhe seus resultados do Teste de Cobertura Facial - Seja um cientista cidadão e veja o seu conhecimento crescer!

Click submit to finish.

Back | Submit | Page 8 of 8

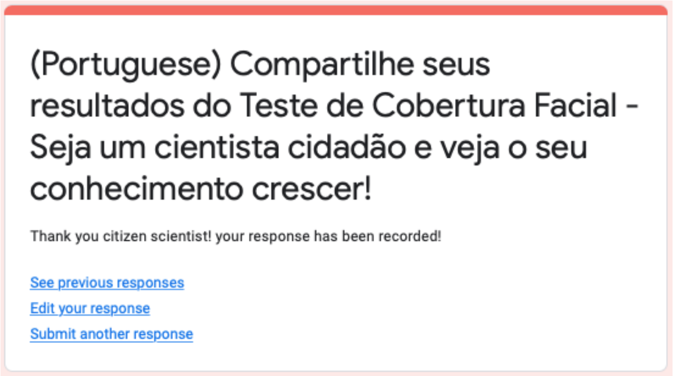

Compartilhe os RESULTADOS

(abaixo está uma série de simulação ou respostas aleatórias para ilustração)
Insira apenas resultados reais para que todos possamos ver as descobertas reais.
Se alguns resultados não forem semelhantes aos seus dados, discuta com um professor.
E pergunte / leia os resultados reais de experimentos de laboratório que foram obtidos em um Centro de Pesquisa Médica.
Os estudos originais são publicados aqui:

https://doi.org/10.3389/fmed.2020.00260

Textile Masks and Surface Covers—A Spray Simulation Method and a "Universal Droplet Reduction Model" Against Respiratory Pandemics. Rodriguez-Palacios A, Cominelli F, Basson AR, Pizarro TT, and Ilic S. (2020) Front. Med. 7:260. doi: 10.3389/fmed.2020.00260

https://doi.org/10.3389/fmed.2020.00504

Germ-Free Mice Under Two-Layer Textiles are Fully Protected From Bacteria in Sprayed Microdroplets: A Functional in-vivo Test Strategy of Facemasks and Filtration Materials. Rodriguez-Palacios A, Conger M, and Cominelli F. (2020) Front. Med. 7:504. doi: 10.3389/fmed.2020.00504

A Citizen Science Facemask Experiment and Educational Modules to Increase Coronavirus Safety in Communities and Schools. Eichler SE, Hopperton AP, Alava JJ, Pereira A, Ahmed R, Kozlakidis Z, Ilic S and Rodriguez-Palacios A (2020) Front. Med. 7:486. doi: 10.3389/fmed.2020.00486
https://doi.org/10.3389/fmed.2020.00486

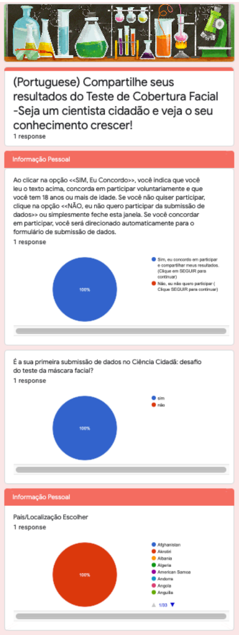

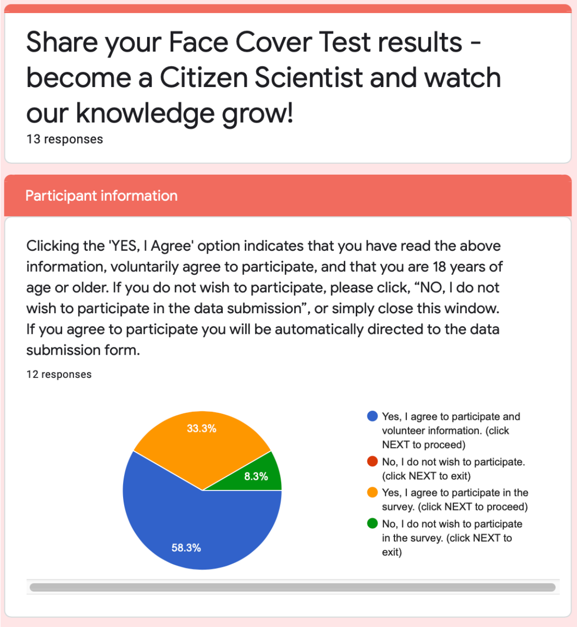

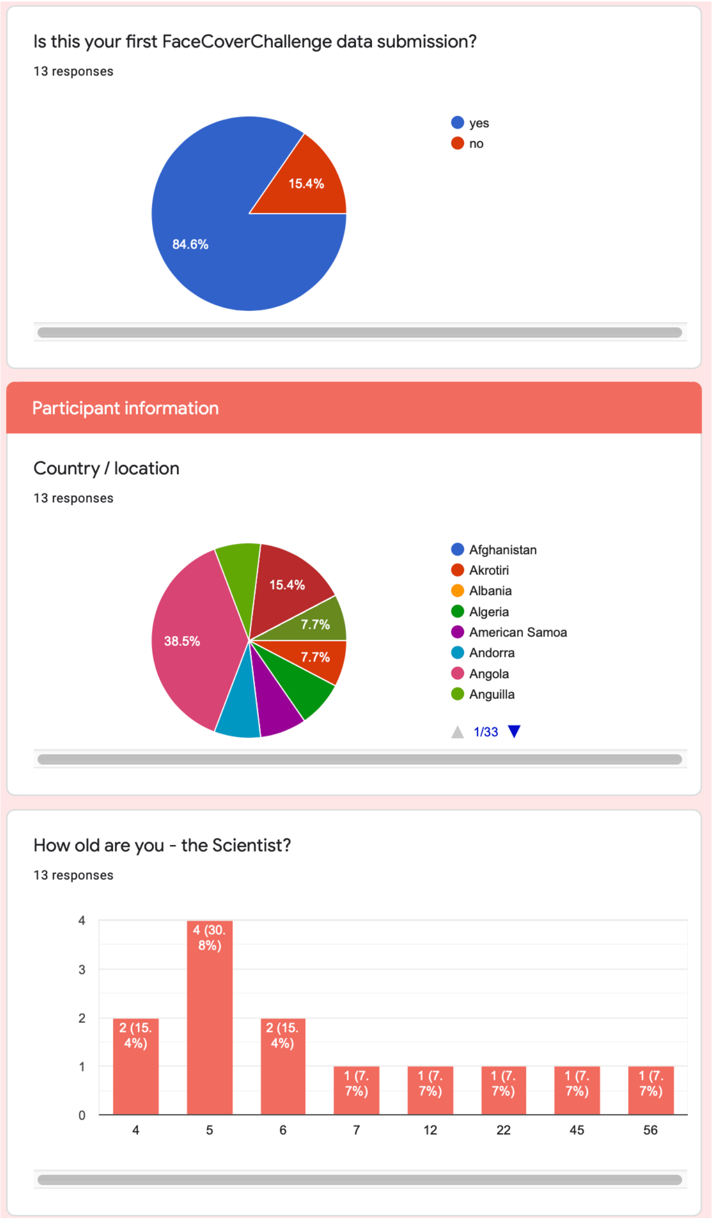
Is this your first FaceCoverChallenge data submission?
13 responses
yes
no
15.4%
84.6%
Participant information
Country / location
13 responses
15.4%
7.7%
38.5%
7.7%
Afghanistan
Akrotiri
Albania
Algeria
American Samoa
Andorra
Angola
Anguilla
1/33
How old are you - the Scientist?
13 responses
4
3
2
1
0
2 (15.4%)
4 (30.8%)
2 (15.4%)
1 (7.7%)
1 (7.7%)
1 (7.7%)
1 (7.7%)
1 (7.7%)
4
5
6
7
12
22
45
56

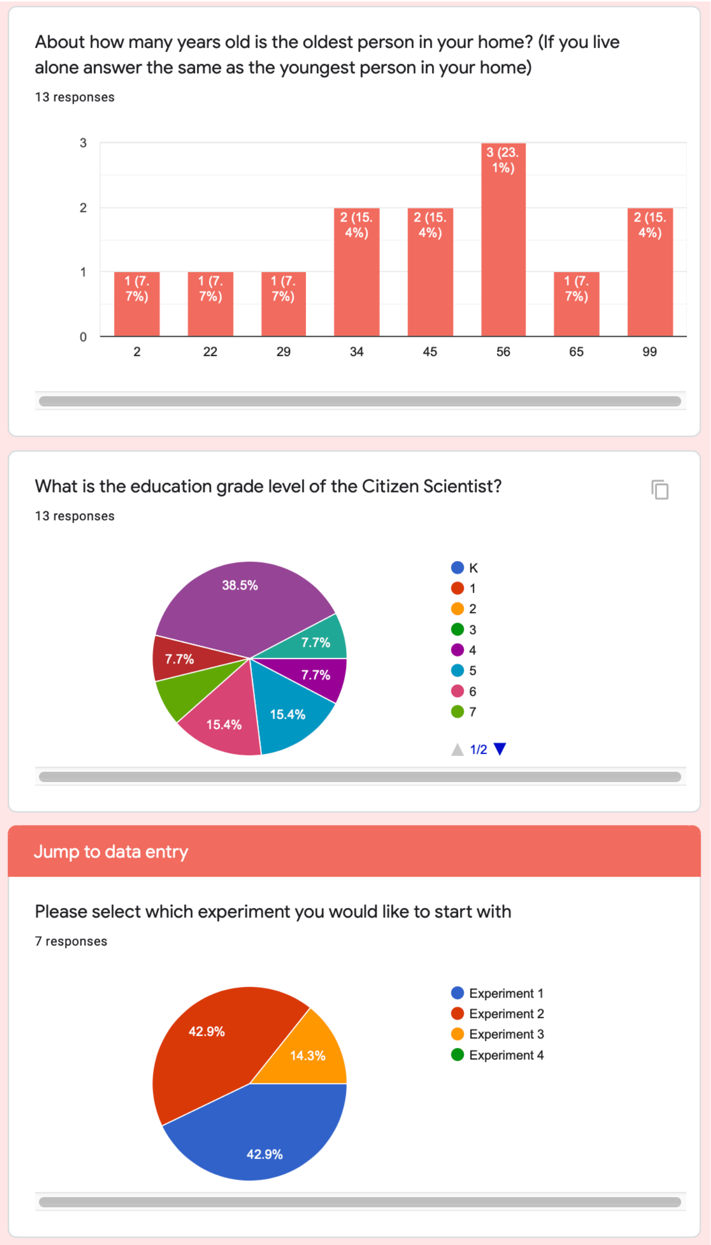

Veja os resultados restantes no link do formulário de resposta
https://docs.google.com/forms/d/e/1FAIpQLSd9cV7HQzxr49MsC-icHCzxIOnlhX2z7e7iza3cJ-NGzJaFRw/viewform
A Citizen Science Facemask Experiment and Educational Modules to Increase Coronavirus Safety in Communities and Schools.
Eichler SE, Hopperton AP, Alava JJ, Pereira A, Ahmed R, Kozlakidis Z, Ilic S and Rodriguez-Palacios A (2020) Front. Med. 7:486.
doi: 10.3389/fmed.2020.00486
SUPPLEMENTARY MODULE - In English.
Provided by Alex Rodriguez-P. axr503@case.edu.
V1 (August 20, 2020) with the accepted publication.
Results submission form in English:
https://docs.google.com/forms/d/e/1FAIpQLSd9cV7HQzxr49MsC-icHCzxIOnlhX2z7e7iza3cJ-NGzJaFRw/viewform

DATA SUBMISSION FORMS in:
English: https://bit.ly/facemaskchallengedata
Français: https://forms.gle/5q7V44nbT8fu6Jvz6
Espanol: https://forms.gle/iWgE8JFeDvezxXTq6
Portugues: https://forms.gle/EJHauCWTdDbny66N8
